# Evolution of cytosolic and organellar invertases empowered the colonization and thriving of land plants

**DOI:** 10.1093/plphys/kiad401

**Published:** 2023-07-11

**Authors:** Hongjian Wan, Youjun Zhang, Limin Wu, Guozhi Zhou, Luzhao Pan, Alisdair R Fernie, Yong-Ling Ruan

**Affiliations:** State Key Laboratory for Managing Biotic and Chemical Threats to the Quality and Safety of Agro-Products, Institute of Vegetables, Zhejiang Academy of Agricultural Sciences, Hangzhou 310021, China; Center for Plant Systems Biology and Biotechnology, Plovdiv 4000, Bulgaria; Department of Molecular Physiology, Max Planck Institute of Molecular Plant Physiology, Potsdam-Golm 14476, Germany; Food and Agriculture, CSIRO, ACT, Canberra 2601, Australia; State Key Laboratory for Managing Biotic and Chemical Threats to the Quality and Safety of Agro-Products, Institute of Vegetables, Zhejiang Academy of Agricultural Sciences, Hangzhou 310021, China; State Key Laboratory for Managing Biotic and Chemical Threats to the Quality and Safety of Agro-Products, Institute of Vegetables, Zhejiang Academy of Agricultural Sciences, Hangzhou 310021, China; Center for Plant Systems Biology and Biotechnology, Plovdiv 4000, Bulgaria; Department of Molecular Physiology, Max Planck Institute of Molecular Plant Physiology, Potsdam-Golm 14476, Germany; State Key Laboratory of Crop Stress Biology in Arid Areas and College of Horticulture, Northwest A&F University, Xianyang 712100, China; Division of Plant Sciences, Research School of Biology, The Australian National University, Canberra, ACT 2601, Australia

## Abstract

The molecular innovation underpinning efficient carbon and energy metabolism during evolution of land plants remains largely unknown. Invertase-mediated sucrose cleavage into hexoses is central to fuel growth. Why some cytoplasmic invertases (CINs) function in the cytosol, whereas others operate in chloroplasts and mitochondria, is puzzling. We attempted to shed light on this question from an evolutionary perspective. Our analyses indicated that plant CINs originated from a putatively orthologous ancestral gene in cyanobacteria and formed the plastidic CIN (α1 clade) through endosymbiotic gene transfer, while its duplication in algae with a loss of its signal peptide produced the β clade CINs in the cytosol. The mitochondrial CINs (α2) were derived from duplication of the plastidic CINs and coevolved with vascular plants. Importantly, the copy number of mitochondrial and plastidic CINs increased upon the emergence of seed plants, corresponding with the rise of respiratory, photosynthetic, and growth rates. The cytosolic CIN (β subfamily) kept expanding from algae to gymnosperm, indicating its role in supporting the increase in carbon use efficiency during evolution. Affinity purification mass spectrometry identified a cohort of proteins interacting with α1 and 2 CINs, which points to their roles in plastid and mitochondrial glycolysis, oxidative stress tolerance, and the maintenance of subcellular sugar homeostasis. Collectively, the findings indicate evolutionary roles of α1 and α2 CINs in chloroplasts and mitochondria for achieving high photosynthetic and respiratory rates, respectively, which, together with the expanding of cytosolic CINs, likely underpin the colonization of land plants through fueling rapid growth and biomass production.

## Teaser

The diversification and expansion of cytosolic and organellar invertases represent a key molecular innovation underpinning efficient carbon and energy metabolism for the evolution of land plants.

## Introduction

The colonization of the planet by land plants represents a key step towards the formation of the terrestrial ecosystem. The evolution of flowering plants from nonvascular land plants and their subsequent domestication underpins modern agriculture. These evolutionary milestones were achieved through corresponding changes or adaptations at the molecular and metabolic levels to optimize resource distribution and energy production (Fernie et al. 2020; Shen et al 2023). To this end, sucrose (Suc) is the most important carbon nutrient and energy currency originating from photosynthesis in most plant species. Thus, understanding the dynamics of Suc metabolism and utilization during evolution may hold the key to deciphering the secret of colonization and subsequent thriving of land plants.

Suc is translocated from source leaves to the heterotrophic sink organs, such as root, fruit, and seeds where the disaccharide is hydrolyzed by invertase (EC 3.2.1.26) into glucose (Glc) and fructose (Fru) or degraded by sucrose synthase (EC 2.4.1.13) into UDP-Glc and Fru (Stitt and Sonnewald 1995). According to their optimum pH, invertases are classified into neutral/alkaline invertases with an optimal pH of 7.0 to 7.8 and the structurally unrelated acid invertases (β-fructofuranosidases) with an optimum pH of 4.5 to 5.5 (Ruan et al. 2010; Wang and Ruan 2016; Li et al 2017). The latter reside in the cell wall or vacuole, hence, named as CWIN for cell wall invertase and VIN for vacuolar invertase, while the neutral/alkaline invertases usually operate in the cytoplasm, hence also called CIN for cytoplasmic invertase (Wan et al. 2018). CIN, CWIN, and VIN are all encoded by the nuclear genomes.

In contrast to the acid invertases that belong to the glycoside hydrolase family (GH) 32 in the carbohydrate-active enzymes (CAZy) database, the CINs are not β-fructofuranosidases and not glycosylated, and they fall in the GH100 family in the CAZy database (Lombard et al. 2014). Phylogenetically, CINs are divided into α and β clades (Ji et al. 2005; Wan et al. 2018). The β clade CINs reside in the cytosol (Roitsch and González 2004), whereas the α clade CINs are localized to mitochondria (Xiang et al. 2011; Martín et al. 2013), chloroplasts (Vargas et al. 2008), or both (Murayama and Handa 2007). The 2 classes of *CIN* genes are different in their intron–exon composition and their encoded amino acid residues (Ji et al. 2005). The diverse gene structure and subcellular location of the encoded proteins, together with the finding that CINs have undergone stronger purifying selection than CWINs and VINs during evolution (Wan et al. 2018), indicate complex roles that CINs may play at the metabolic and cellular levels. In this context, knockout of β clade CINs disrupt reproductive and root development in Arabidopsis (*Arabidopsis thaliana*, Barratt et al. 2009) and a model legume, *Lotus* (*Lotus japonicus*, Welham et al. 2009), while deletion of α clade mitochondria CINs elicits oxidative stress in Arabidopsis (Xiang et al. 2011) and reduced respiration, which was found to severely compromise the energy-demanding processes of seed germination and flowering (Martín et al. 2013). On the other hand, a plastidic α clade CIN has been shown to be required for starch accumulation (Vargas et al. 2008) and the development of the photosynthetic apparatus in Arabidopsis (Tamoi et al. 2010). Despite these observations, the potential importance of CINs, especially that of plastidic and mitochondrial CINs, in the evolution of plant energy metabolism remains unknown.

Taking advantage of the vast genomic and functional genomic data available, we aimed here to examine the global scale evolution and functional relation of CINs by exploring the phylogenetic relationships and evolutionary patterns of α and β clade CINs from algae to seed plants and linking their evolution with the energy metabolism and growth of land plants and their terrestrial colonization.

## Results

### Plant CINs emerged early in streptophytes after endosymbiotic origin

**Figure 1.**
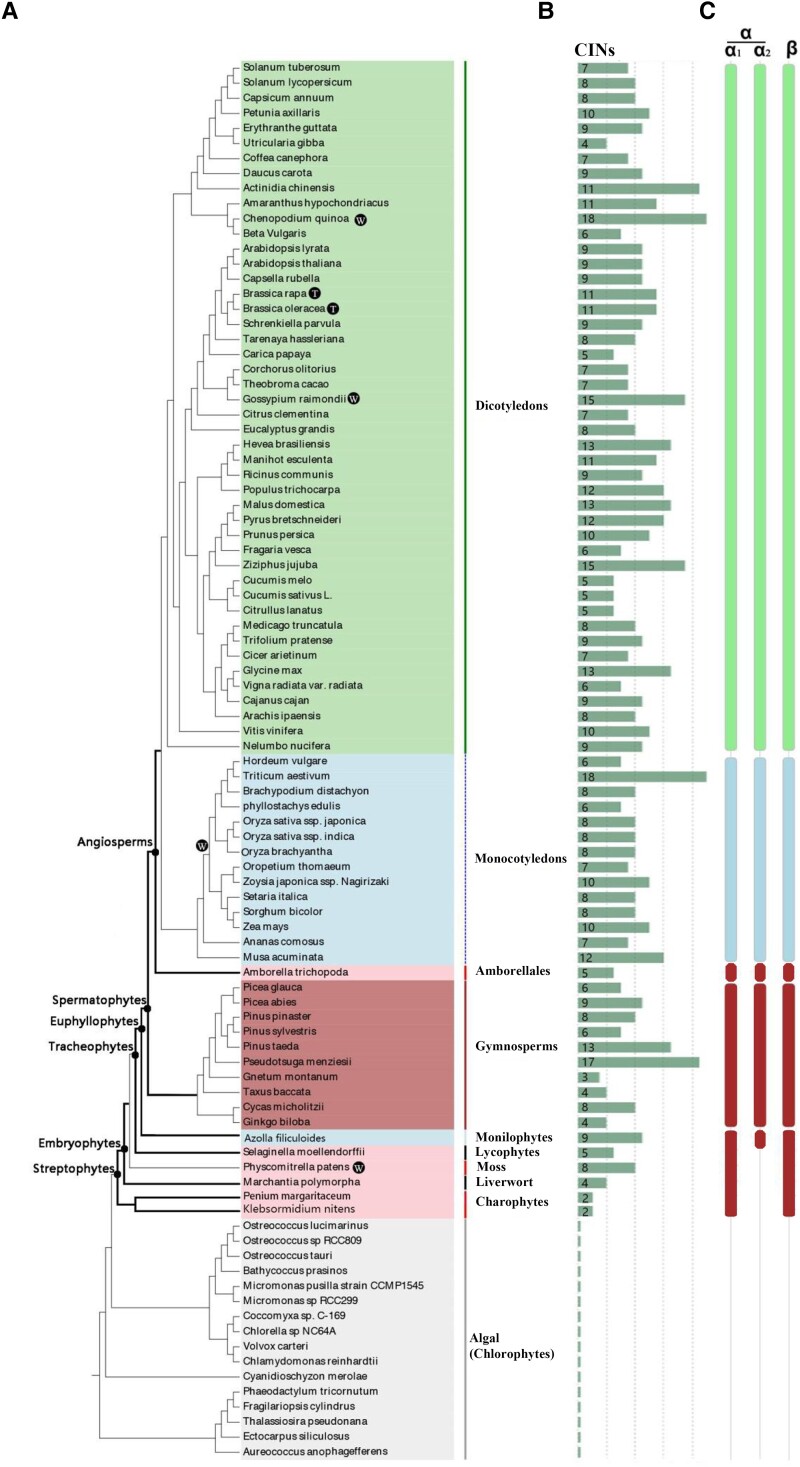
Expansion of CIN proteins in the green lineage. A simplified phylogeny of the green lineage was constructed. The numbers of CIN proteins were identified from different species with their genomes sequenced, which included chlorophytes, charophytes, mosses, lycophytes, monilophytes, gymnosperms, monocots, and dicots. **A)** A total of 93 plant species were selected. W, whole genome duplication; T, triplication. **B)** Number of CINs in different plant species. **C)** The CINs from different plant species belonging to α (α1 and α2) and β subfamilies.

To identify putative CIN homologs, we searched 93 sequenced plant genomes. Through 2 rounds of analyses using *blastp*, we identified 665 predicted CIN homologous sequences from 77 species including charophytes, nonvascular, and vascular plants (Fig. 1B and Supplemental Table S1). No CIN was found from the 16 green algae chlorophytes and distal from the evolution of land plants. Interestingly, 2 CINs were identified from each of the 2 charophytes species, *Klebsormidium nitens* and *Penium margaritaceum* (Fig. 1 and Supplemental Table S1), living in fresh water (Hori et al. 2014). The analysis indicates that CIN proteins were absent from the chlorophyte species examined but appeared in charophyte just prior to the emergence of land plants and persisted in all the embryophytes examined (Fig. 1 and Supplemental Table S1). Consistently, the genome of the charophyte species, *P. margaritaceum*, indeed carries molecular hallmarks of the origins of land plants including those involved in carbon metabolism and protection against water loss and UV (Jiao et al. 2020). All the CIN proteins were divided into α and β subfamilies (Fig. 1C) with the former to be targeted to plastids and/or mitochondria and the latter to be cytosolic as previously reported (Wan et al. 2018).

**Figure 2.**
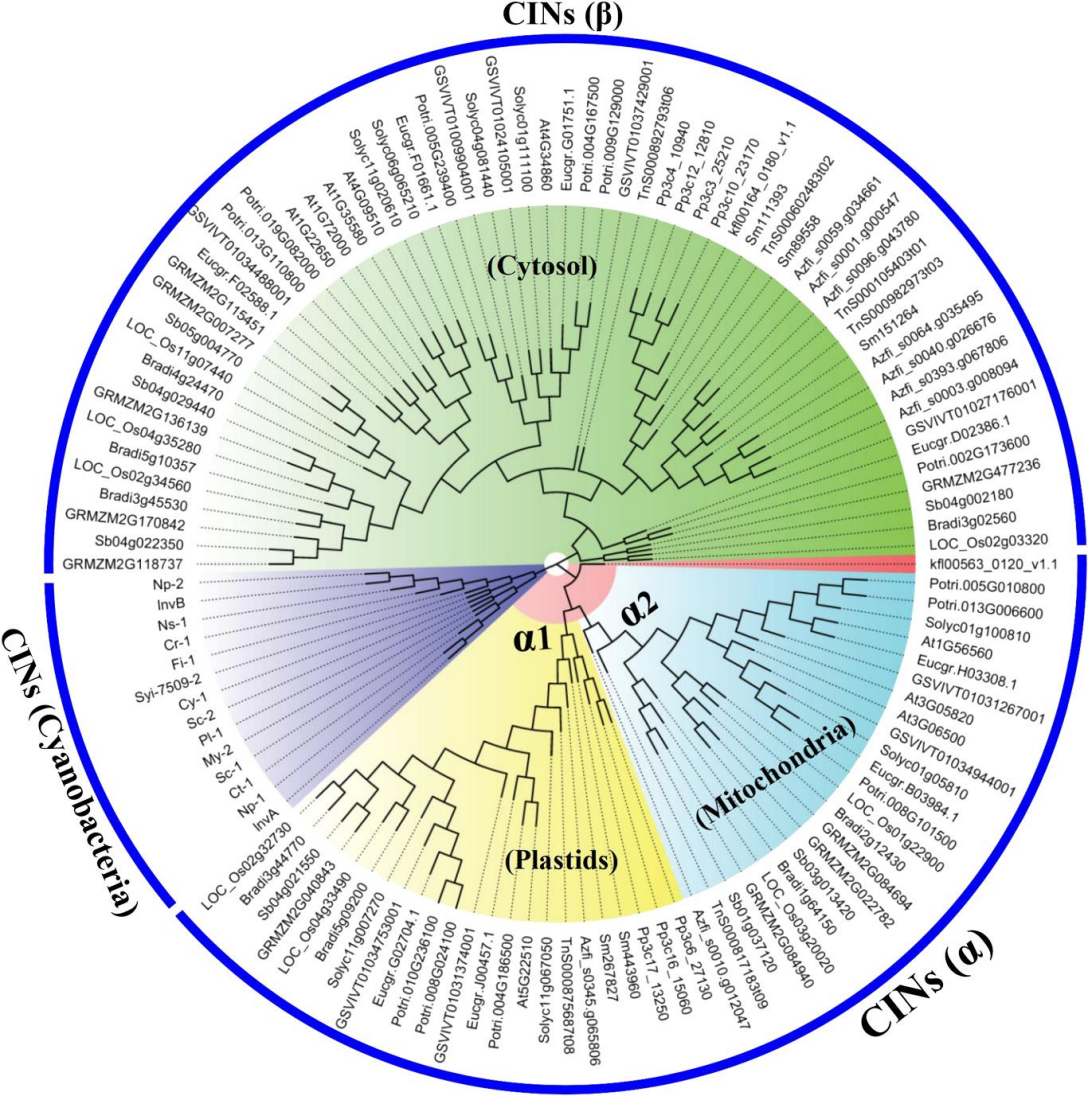
Evolutionary relationship of CIN genes from algae to vascular plants. The ML method was adopted to construct the phylogenetic tree. Bootstrap consensus tree inferred from ML analysis, based on the JTT matrix-based model. Bootstrap confidence values were obtained by 1,000 replicates. Branches corresponding to partitions reproduced in less than 50% bootstrap replicates are collapsed. A discrete gamma distribution was used to model evolutionary rate differences among sites (5 categories) (+G, parameter = 0.9923). The analysis involved 121 amino acid sequences, with a total of 2,184 positions. Evolutionary analyses were conducted using MEGA7.0 and RAxML v8.2. The sequences of CINs are from the following species: a charophyte, *K. nitens*, a moss, *P. patens*, and a lycophyte, *S. moellendorffii* and 7 vascular plant species: rice (*O. sativa*), *Brachypodium distachyon*, sorghum (*S. bicolor*), maize (*Z. mays*), Arabidopsis (*A. thaliana*), grape (*V. vinifera*), *poplar* (*P. trichocarpa*), *E. grandis*, and tomato (*S. lycopersicum*), *Anabaena* sp. PCC 7120 (InvA, WP_010995690.1; InvB, CAC85155.1), *C. raciborskii* CS-505 (Cr-1, ZP_06306902.1), *C. thermalis* (Ct-1, WP_015156189.1), *Cyanothece* sp. PCC 7822 (Cy-1, WP_013325329.1), *Fischerella* sp. JSC-11 (Fi-1, ZP_08987807.1), *Myxosarcina* sp. GI1 (My-2, WP_036476871.1), *N. punctiforme* ATCC 29133 (Np-1, CAD37134.1; Np-2, CAD37133.1), *N. spumigena* CCY9414 (Ns-1, ZP_01631199.1), *Pleurocapsa* sp. PCC 7319 (Pl-1, WP_019507642.1), *S. cyanosphaera* (Sc-1, WP_041619725.1; Sc-2, WP_015195523.1), and *Synechocystis* sp. (PCC 6803, Syi-6803-1). The CIN protein sequences used are listed in Supplemental Table S1.

We further identified many CIN homologs from the genomes of cyanobacteria and proteobacteria, but not from those of noncyanobacterial and nonproteobacterial prokaryotes (Supplemental Table S1). Phylogenetic analyses of CIN proteins from the sequenced genomes of cyanobacteria and plant species from charophytes to angiosperms (Fig. 2) revealed that the CINs from cyanobacteria were species-specific and placed basally with respect to plant species, whereas the plant CIN family members were divided into α and β families (Fig. 2). The presence of CIN homologs in those 2 prokaryote species and streptophytes (Supplemental Table S1), phylogenetically located at the base of the cyanobacterial radiation (Fig. 2) (Palenik and Haselkorn 1992; Urbach et al. 1992), shows that CINs most likely originated from an ancestral CIN-like gene in cyanobacterial genomes and modern plant CINs may have originated from an orthologous ancestral gene in charophytes after the endosymbiotic origin of chloroplasts.

### CIN family expanded through 2 rounds of lineage-specific duplication characterized by loss of the N-terminal transit peptide or an alteration in its charge status

Further phylogenetic analyses revealed that the α group was separated into 2 subgroups (α1 and α2) supported with high bootstrap value (Figs. 2 and S1), indicating the likelihood that 2 rounds of lineage-specific duplication had occurred during evolution. Importantly, the CINs from charophyte algae are placed basally with respect to land plants in the α and β subfamilies (Fig. 2), which suggests that this group originated before the diversification of the streptophytes (charophyte algae and land plants).

**Figure 3.**
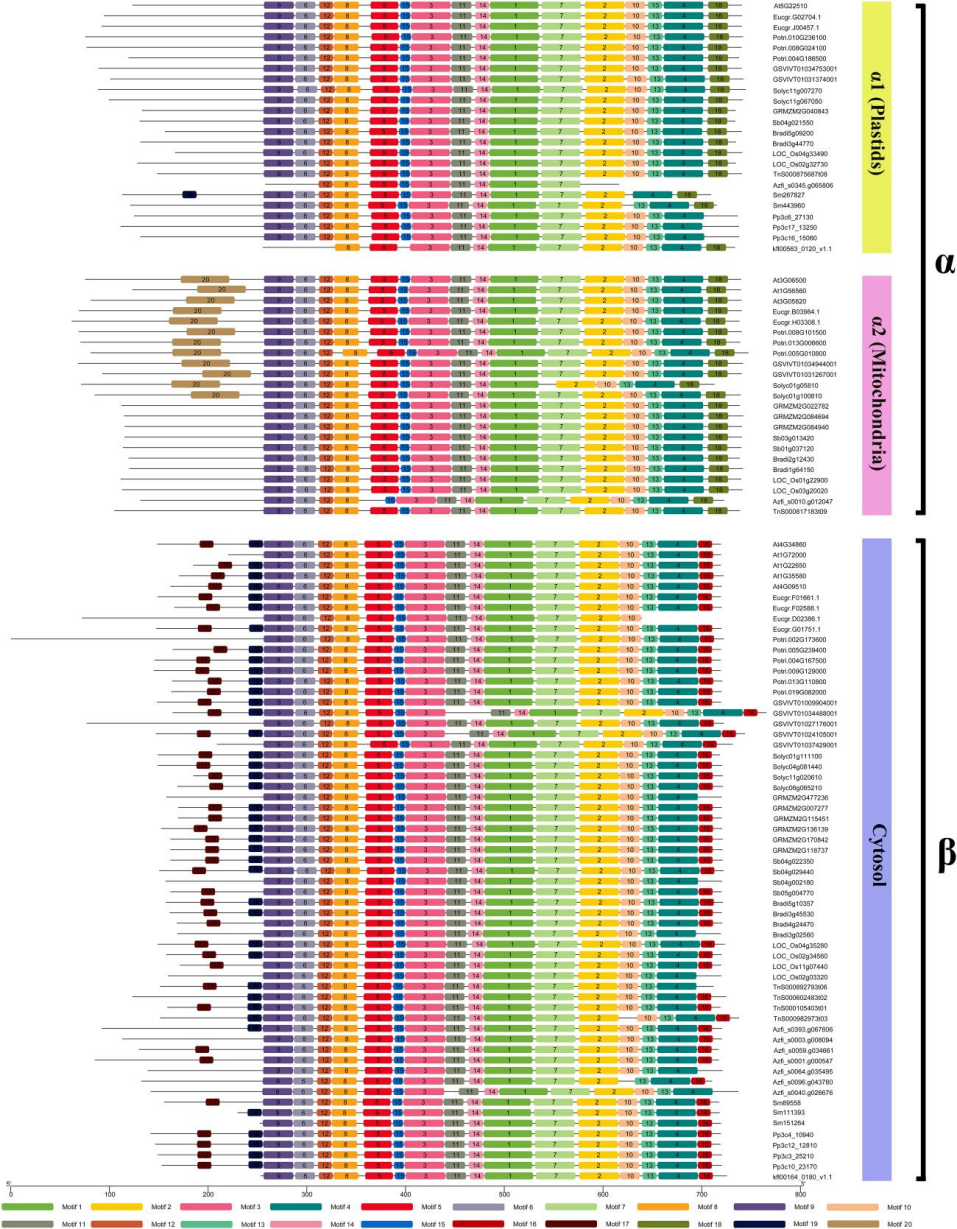
Motif patterns of the CINs across streptophytes. Motif patterns of the CINs were analyzed using online tools (https://meme-suite.org/meme/). Twenty distinct MEME motifs are displayed in different boxes. The sequence information for each MEME motif is provided in Supplemental Table S4. The length of the protein can be estimated by using the scale at the bottom. Motif 18 and motif 16, located on the C-terminus of CINs, belonged to α and β subfamilies.

**Figure 4.**
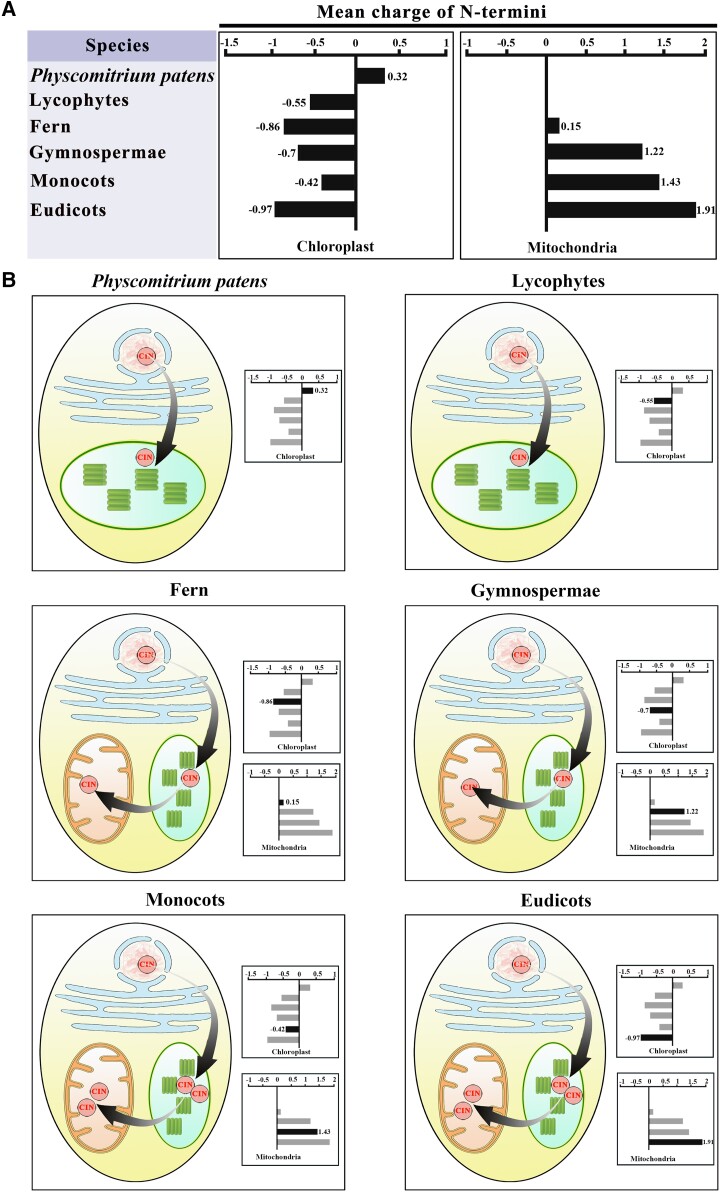
Average charge of N-terminal sequences (signal peptide) of plant CINs targeting mitochondria and plastids. **A)** Bar diagram showing the mean charges of the different N-terminal sequences (NTSs) described the text. The CIN proteins were selected from bryophytes to vascular plant. **B)** Plastid acquisition through endosymbiosis requires the cells to discriminate nuclear-encoded mitochondrial proteins from nuclear-encoded plastid proteins, by means of positive charges in the NTS. Some nuclear-encoded proteins are targeted to both organelles simultaneously, and their NTSs carry a charge on average ranging between those of proteins targeted exclusively to mitochondria or plastids. This relaxes the selection pressure on their NTS. The gradual arrows indicate the flow of CIN targeting from nuclei to plastid and mitochondria. Square boxes indicate the approximate positive charge of the NTS, while the blowups show the protein import machineries of the respective organelles.

In view of the substantial evidence on the cyanobacterial origin of plant chloroplasts and the fact that genes could be transferred from the chloroplast to the nuclear genome (Douglas 1994; Martin and Herrmann 1998), it seems likely that plants acquired CIN genes during the endosymbiotic origin of the chloroplast. Thus, we further investigated the evolutionary relationship of CINs located in different organelles in plant species. Among these 2 groups of CINs, 1 is targeted to the cytosol (β) and the other (α1 and α2) to plastids and mitochondria (Supplemental Table S2). Here, we obtained further evidence that supports this view, that the α1 subfamily CINs carry transit peptides at their N-terminus, which target them to the plastids, while the cytosolic CINs lack these targeting sequences (Figs. 3 and 4 and Supplemental Table S2). Thus, a gene encoding a cytosolic CIN could, in theory, evolve from a gene encoding a plastidic CIN with the transit peptide being lost. To test this possibility, we compared the N-terminal CIN sequences among the 15 selected plant species ranging from algae, nonvascular, to vascular plant species (Supplemental Table S2 and Fig. 3). The analyses revealed that the N-terminal sequences of α1 CIN (plastidic) were, on average, 100 bp longer than that of the β (cytosolic) proteins. *In silico* subcellular localization was determined using the SignalP tools, which predicted that α1 CINs were localized in the chloroplast whereas the CINs without targeting sequence at N-terminus were mapped to the cytosol (Supplemental Table S2). These data indicate that the cytosolic CIN gene evolved from a duplicate of an ancestral gene encoding a plastidial CIN subsequently losing the transit peptide sequence during the charophyte to land plant transition. It remains to be experimentally confirmed if deletion of the transit peptide would shift the localization of α1 CIN from plastids to cytosol.

It is intriguing that vascular plants also carry α2 CINs localized to mitochondria in addition to the plastidic α1 CIN (Xiang et al. 2011; Martín et al. 2013). Understanding the evolutionary relationship between α1 and α2 CINs may provide clues on their functional roles. Generally, genes encoding proteins imported to plastids and mitochondria are of independent origin with a high degree of similarity in N-terminal targeting sequences (Garg and Gould 2016). It is, therefore, challenging to examine the evolutionary relationship between α1 and α2 CINs based on N-terminal sequence. To circumvent this difficulty, we examined the charge status of their targeting sequences because those proteins from the mitochondrial matrix, but not plastidic lumen, are positively charged with an enrichment of basic amino acids (Garg and Gould 2016). Our analyses revealed that the α1 subfamily of plastidic CINs, except that from *Physcomitrium patens*, has a charge value of less than 0 (mean charge of −0.04), whereas the charges of the α2 subfamily located in mitochondria carry charge greater than 0 (mean charge of 1.18, Fig. 4 and Supplemental Table S3).

### Variation in motif composition of CINs may contribute to subcellular targeting and optimum pH for catalytic activity

To better understand the functional conservation and divergence of CIN genes, we examined the sequence features of the CIN proteins from different plant lineages. It was found that the composition and organization of conserved motifs differed substantially among α and β families (Fig. 3 and Supplemental Table S4). As expected, the most closely related CIN proteins from the same orthology group share common motifs, suggestive of functional similarities. However, obvious differences in motif distribution were observed among these 2 subfamilies. For example, 3 motifs (motifs 16, 17, and 19) belonged to the β subfamily, whereas motifs 18 and 20 belong to the α subfamily (Fig. 3). Among these 5 motifs, motifs 17, 19, and 20 were mapped at the N-terminus, probably related to different subcellular localization of the α and β CINs, while motifs 16 and 18 were located at the C-terminus. Interestingly, among 6 Arabidopsis LIM (Linl-1, Isl-1 and Mec-3) family members, the C-terminal (Ct) domain of PLIM2c, expressed in pollens, is able to inhibit LIM actin-bundling activity in a pH-dependent manner (Moes et al. 2015). Therefore, we proposed that the C-terminal motifs 16 and 18 in β and α CINs, respectively, could be important for CIN-catalyzed Suc hydrolysis in a pH-dependent manner. Consistent with this view is the finding that the pH of plant cytosol is nearly neutral (7.3), while the mitochondrial matrix and plastidial stroma have alkaline pH (Shen et al. 2013). Thus, the comparisons are in keeping with early proposals that CINs in the cytoplasm and organelles (mitochondrial matrix and plastids) represent neutral and alkaline CINs, respectively.

### Structural evolution of CIN genes: differential patterns of intron loss and conservation of amino acid residues

**Figure 5.**
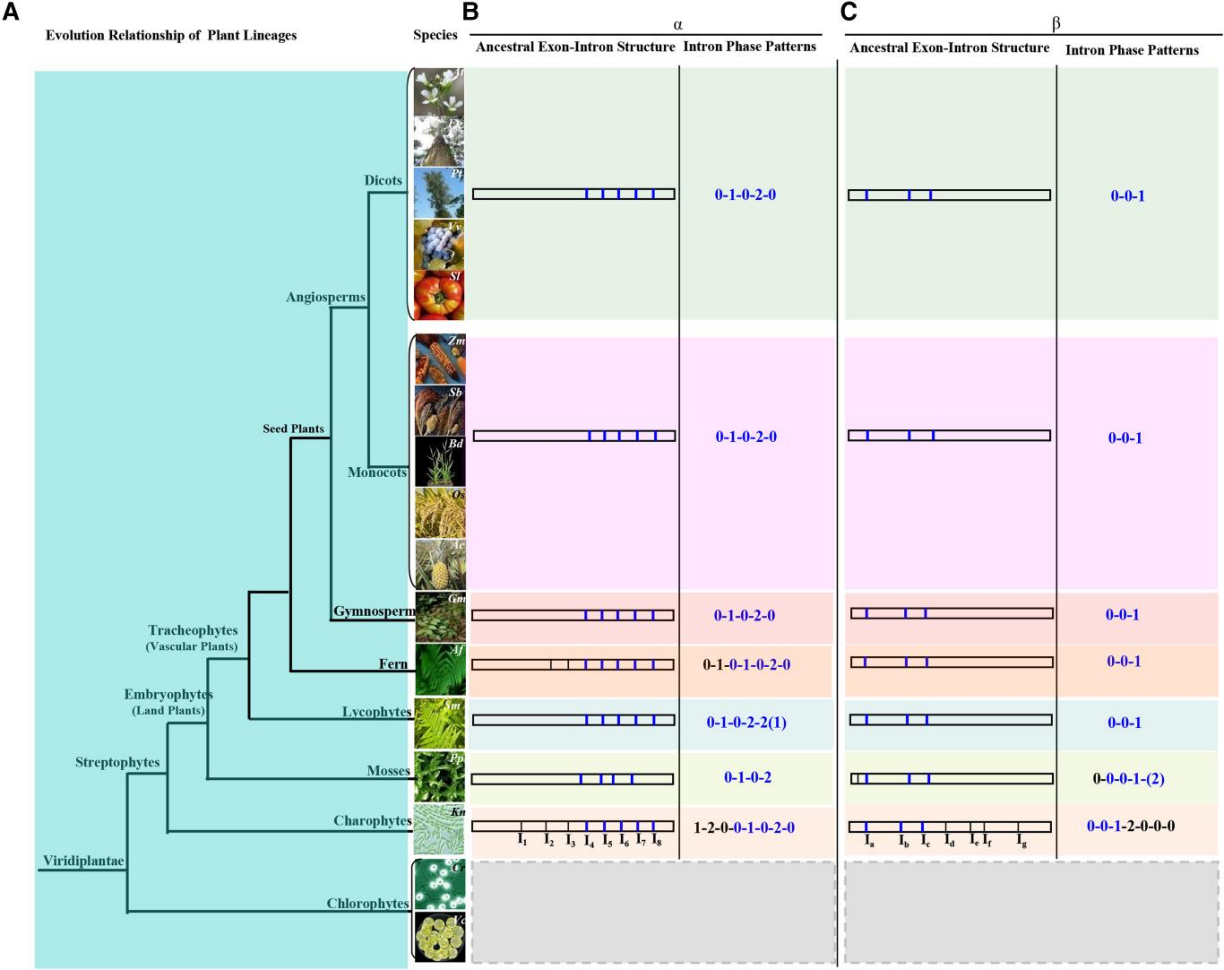
An evolutionary model for the structural changes of CINs in plants. **A)** Evolutionary relationship of all plant lineages. The phylogenetic tree was constructed based on information from Phytozome (http://www.phytozome.net). See Fig. 1A legend for species name abbreviations and lineage. Two additional chlorophytes were added, including *Chlamydomonas reinhardtii* (Cr) and *Volvox carteri* (Vc). **B)** Proposed exon–intron structure of the ancestral α clade of CINs in each plant lineage. **C)** Proposed exon–intron structure of the ancestral β clade of CINs in each plant lineage. I, intron. The introns labeled in blue are present in the streptophytes in **B)** and **C)**. Note: no CINs were identified from chlorophytes (**B** and **C**).

The functional conservation and divergence of paralogous genes is reflected not only in coding sequences but also in the exon–intron structure of the genomic DNA (Xu et al. 2012). Indeed, structural divergence is common in duplicated genes and could lead to functionally divergent paralogs (Xu et al. 2012). We thus further examined the exon/intron organizations of α and β subfamilies of CIN genes. Although most members within each orthology group exhibit a similar exon/intron organization in terms of exon length, intron number, and intron phase, this structure organization varied among different orthology groups (Fig. 5). In the α subfamily, the green algae *K. nitens* has 8 introns (I_1_ to I_8_), while only 5 introns (I_4_ to I_8_) were retained in gymnosperms (e.g. *Gnetum montanum*) and angiosperms (e.g. *Oryza sativa* and *A. thaliana*). The corresponding intron phases also changed from 1-2-0-0-1-0-2-0 to 0-1-0-2-0. This suggests that intron loss events have occurred during evolution. Similarly, intron loss events also were observed for β CIN subfamilies *K. nitens* (I_a_ to I_g_) and tracheophytes (I_a_ to I_c_) with the corresponding intron phases being 0-0-1-2-0-0-0 and 0-0-1, respectively. Although the event of intron loss was observed in both α and β CINs, it displayed clear differences in intron phases and numbers, as well as the number and location of intron losses (Fig. 5). For example, the α CINs lost the intron at the N-terminus whereas in the β subfamily, it occurred at the C-terminus.

It is well known that catalytic amino acid residues play key roles in enzymatic reactions. Multiple sequence alignment of CIN amino acid sequences showed that 3 amino acid residues (D188, E414, and Arg430), associated with catalytic activity (Xie et al. 2016), were highly conserved in both α and β subfamily, whereas amino acid residues Ser547 and T294 were present in β and α subfamily, respectively (Supplemental Fig. S2).

### Coevolution of α2 CINs with vascular plants and differential expansion of CIN subfamilies

In contrast to α1 and β subfamilies, no α2 CINs were identified from algae (*K. nitens* and *P. margaritaceum*), a nonvascular plant species, *P. patens* (a moss), and a member of a basal vascular plant lineage, *Selaginella moellendorffii* (Fig. 2). The reason for the observed absence of the member of α2 subfamily could be due to either poor annotation of their genomes or the lack of the CIN genes from α2 subfamily in these species. To distinguish these 2 possibilities, we firstly repeated our in silico analysis of these 4 genomes (*K. nitens*, *P. margaritaceum*, *P. patens*, and *S. moellendorffii*) and their transcriptomes using the AT1G56560, AT3G05820, and AT3G06500 protein sequences as queries from *A. thaliana*, which belong to the α2 subfamily. Basic local alignment search tool (BLAST) was used to retrieve best reciprocal BLAST hits for CINs. The result showed that at least 17 members of CINs were identified from these 4 species. Further analysis clearly revealed (i) that these candidate CIN gene products were not predicted to localize in mitochondria by using the SignalP tools (Supplemental Table S2) and (ii) phylogenetic tree of CINs from other sequenced charophytes, moss, and lycophytes species also did not show α2 CINs (Fig. 2). Given these findings, we discounted the possibility that the absence of α2 CINs in nonvascular plant species was due to poor annotation. Thus, we concluded that members of the α2 subfamily of CINs were lacking in nonvascular plant species and coevolved in vascular plants.

To investigate copy number variation of *CINs* during evolution, we conducted a comprehensive search for CIN genes across plant lineages, from charophytes, Bryophyta, Pteridophyta, Gymnospermae to eudicots and monocots (Supplemental Table S2). Our results revealed that the CIN gene family expanded dramatically during plant evolution, with the number of CIN genes ranging from 2 in charophytes (*K. nitens*) to 12 in the eudicot, *Populus trichocarpa*, with most angiosperms having at least 8 members of CIN gene family (Supplemental Table S2). This expansion of the CIN family matches the rapid increase in photosynthetic and respiration rates during evolution (Supplemental Table S6). Notably, copy number of mitochondrial and plastidic CINs (α clade) collectively doubled from ferns to angiosperms, whereas that of cytosolic CIN (β clade) increased from 1 in charophytes to 4 in gymnosperms and remained steady thereafter following the appearance of α2 CINs (Supplemental Table S2 and Supplemental Fig. S3).

### Identification of CIN-interacting proteins by affinity purification mass spectrometry

**Figure 6.**
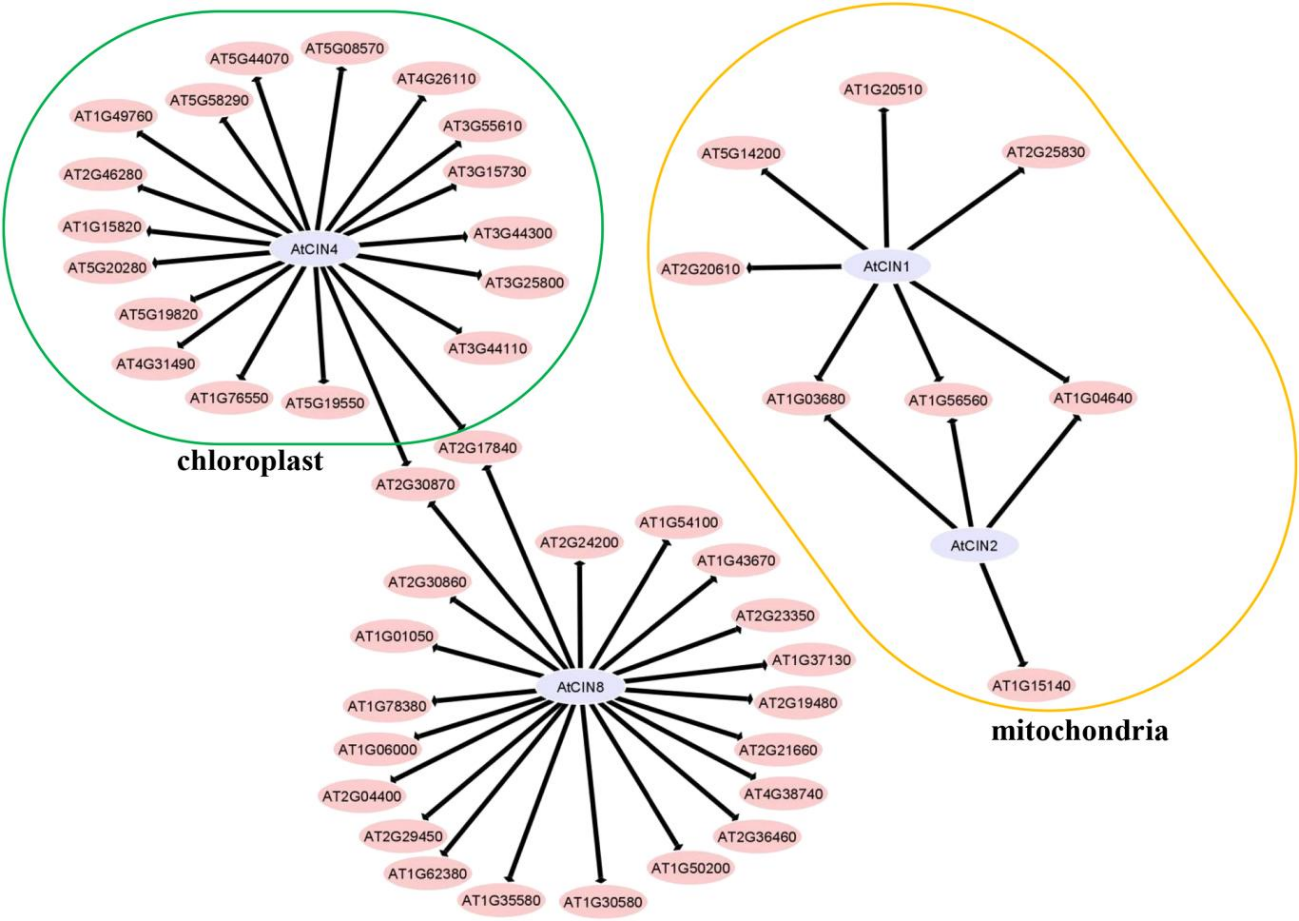
Identification of protein interaction network with mitochondrial AtCIN1 and ATCIN2, chloroplast AtCIN4, and cytosolic AtCIN8 in Arabidopsis leaves by using affinity purification mass spectrometry. Annotations of interacting proteins highlighted correspond to those in the Arabidopsis TAIR database (https://www.arabidopsis.org/index.jsp) and supplement data in Supplemental Table S7.

To gain functional insights into the organellar and cytosolic CINs, we transformed Arabidopsis leaves with GFP fusion constructs linked with mitochondrial AtCIN1 or 2, plastidic AtCIN4, and cytosolic AtCIN8 to discover potential CIN-interacting proteins. Both GFP and AtCIN-GFP could be expressed to a high level in the Arabidopsis leaves. Given that affinity purification mass spectrometry (AP-MS) experiments have been widely used to generate meaningful interaction networks (Puig et al. 2001; Bürckstümmer et al. 2006; Morris et al. 2014; Zhang et al. 2017, 2018, 2019), it follows that they could be used to produce information-rich data concerning both within pathway and extra-pathway protein–protein interactions. Such interactions would aid in the characterization of interacting protein function, provide detailed catalogs of proteins involved in forming protein complexes and reveal networks of biological processes at local and proteome-wide scales (Morris et al. 2014). In the subsequent data analysis, normalized signal intensities were processed to determine fold change abundance (FC-A) scores by the use of the SAINT algorithm embedded within the CRAPome software (Choi et al. 2012; Morris et al. 2014). A total of 331 protein–protein interactions were obtained, displaying in excess of 4-time changes (Supplemental Table S5). Screening of the SUBA4 database (Hooper et al. 2016), a total of 51 interactions were revealed between different subcellularly localized AtCINs and their respective target proteins, comprising the interaction network (Fig. 6 and Supplemental Table S7). Interestingly, both a lipoyltransferase and an m-type thioredoxin as well as a NAD(P) oxidoreductase interacted with AtCIN1 and AtCIN2 in the mitochondria. AtCIN8 interacted with 21 cytosol proteins, and AtCIN4 interacted with 24 chloroplast proteins. The cytosolic proteins included phospholipase D, glutathione transferases, the important hormone biosynthesis enzyme nitrilase, a proline biosynthesis enzyme, pyruvate kinase and phosphofructokinase, sucrose phosphate synthase, aspartate amino transferase, ATPase, the DNA J chaperone, and a senescence-associated gene-related protein. The chloroplast-based interactors with AtCIN4 included glutathione transferases, a senescence-associated protein, aldolases, alanyl-tRNA synthase, an ACC oxidase that catalyzes the final step of ethylene biosynthesis, and the essential N assimilation enzyme, nitrate reductase. While the function of the cytosolic interacting proteins is often related to energy metabolism, that of the mitochondria and plastid seems to be highly enriched in redox components, suggesting that the organellar invertases may have a function in regulating the subcellular redox poise.

### A model for the evolutionary history of the CIN gene family

**Figure 7.**
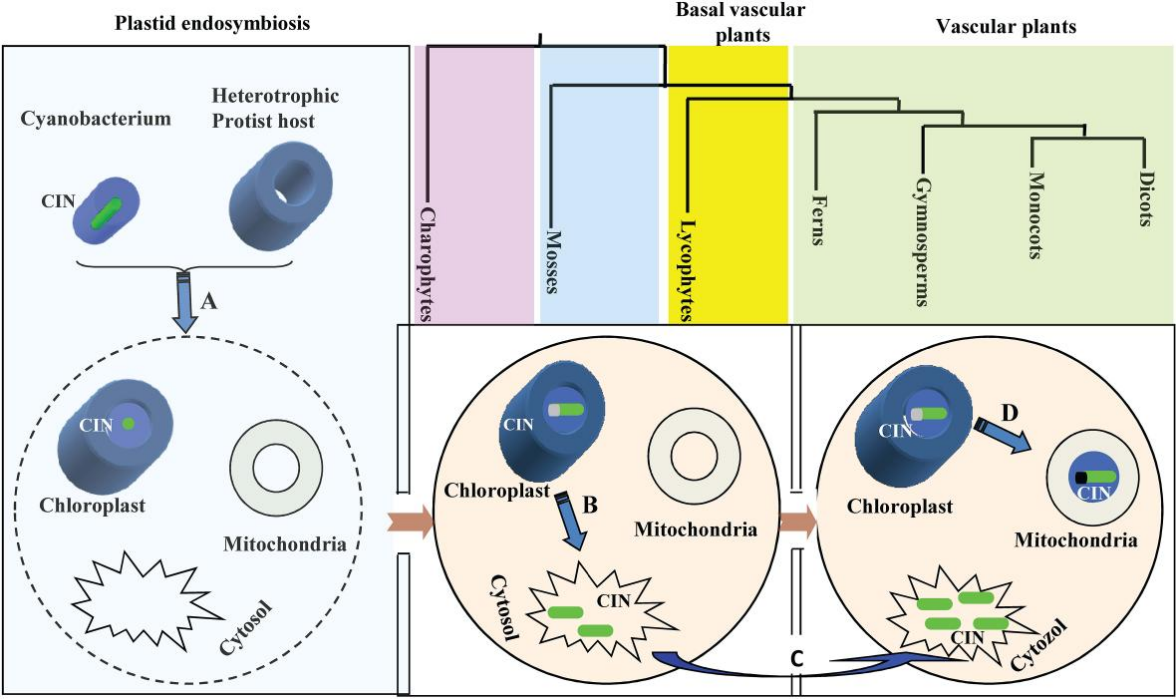
Origin and evolution of CIN gene family. Plant CINs originated from an orthologous ancestral gene after the endosymbiotic origin of chloroplasts. In charophytes, mosses and basal vascular plants, duplication of CINs (α1 clade) from chloroplast produced CINs in the cytosol with a loss of signal peptides from N-terminals (β clade). No α2 CINs were identified in Selaginella moellendorff, a basal vascular plant. In vascular plants, duplication of CINs (α1) from chloroplast produced CINs in the mitochondria (α2). **A)** Endosymbiotic gene transfer. **B)** The first gene duplication event resulted in the loss of signal peptides, hence the appearance of β CIN in the cytosol. **C)** Whole genome duplication. **D)** The second gene duplication event leads to the creation of CINs in the mitochondria (α2).

Based on the above analyses, we propose a plausible scenario of the evolutionary history of the CIN gene family (Fig. 7). In this model, plant CIN genes originated through endosymbiotic gene transfer and evolved from charophytes. These CIN genes originated prior to the diversification of extant streptophytes. The duplication of an ancient CIN gene before the divergence of charophytes and land plants gave rise to 2 lineages of CIN genes, α and β. In charophytes, α and β were maintained as single-copy genes before their separation from streptophytes. In the land plant lineage, the α subfamily experienced additional lineage-specific duplication events, producing the α1 and α2 clades. Of them, coevolution of the mitochondrial α2 CINs with vascular plants is of particular interest. Moreover, the β CIN subfamily expanded steadily from charophytes to gymnosperm and remained steady during the evolution from gymnosperm to angiosperm, whereas the α subfamily was expanded further from gymnosperm to angiosperm (Figs. 7 and S3).

## Discussion

The evolutionary and molecular innovations that enabled plants to colonize the terrestrial planet are not well understood. While the genome of a charophyte, *P. margaritaceum*, displays some hallmarks of the origins of land plants, including genes for cell wall remodeling and adaptation to nonaquatic environment (Jiao et al. 2020), it remains largely unknown regarding the key features underpinning efficient carbon and energy metabolism during evolution, essential for the rapid growth and colonization of vascular plants (Gago et al. 2019). Invertase is biochemically well known for its central role in energy metabolism. Given that it is generally accepted that Suc degradation into hexoses for glycolysis occurs in cytosol (Ruan 2014), it remains puzzling why there are CINs operating in mitochondria and plastids in addition to that in the cytosol. Here, we addressed this unresolved question from genomic and evolutionary perspective. The analyses provided several important insights into the phylogenetic and functional relation among different classes of CINs, which indicates that the evolution and diversification of cytosolic and organellar invertases empowered the colonization and the subsequent thriving of land plants.

### Independent evolution of CIN gene family after endosymbiotic origin signifies functional divergence in sucrose metabolism

Phylogenetic analyses show that the plant CINs apparently were separated into 2 distinct subgroups with strong bootstrap support (Figs. 1 and 2), indicating an independent evolution of the CINs in plant species. All CIN genes located to chloroplasts and mitochondria form a single α gene clade and are monophyletic with the cytosolic β clade CIN genes (Figs. 1 and 2). This suggests that the CINs may have originated from an ancestor before the divergence of charophytes, but diverged later and evolved independently.

The analyses on the structure of CIN genes revealed several substantial differences between α and β subfamilies. This includes variation in intron number and patterns of intron phase as well as the positions of intron loss, which occurred in the N-terminal of the α subfamily, but at the C-terminal of the β subfamily (Fig. 5). These structural differences support the independent evolution of CIN genes from α and β subfamilies.

Furthermore, 3 amino acid residues (D188, E414, and Arg430) known to be associated with catalytic activity (Xie et al. 2016) were highly conserved in all α and β subfamilies of CINs (Supplemental Tables S1 and S2). However, the amino acid residue (Ser547), which was involved in the regulation of CIN activity through phosphorylation (Gao et al. 2014), was conserved only in the β subfamilies, while Cys 294, its substitution with Tyr inhibited chloroplast development (Tamoi et al. 2010), was conserved only in the α subfamily. In addition, among the 20 Multiple Expectation Maximization for Motif Elicitation (MEME) motifs identified, 2 (motif 18 and motif 20) and 3 (motif 16, motif 17, and motif 19) belong to α and β subfamily, respectively (Fig. 3). These findings indicate independent functional divergence of α and β CINs.

### A stepwise duplication of *CIN* copy number corresponds increased photosynthetic and respiration rates that underpin colonization of the land by vascular plants

Photosynthesis and respiration comprise the core pathways of primary carbon and energy metabolism in plants, providing ATP, reducing power [NAD(P)H] and carbon intermediates essential for growth and development. It was reported that gene duplications that altered copy number were found to modify phenotypic diversity in plants (Lye and Purugganan 2019). For example, in the vegetable and oilseed allopolyploid *Brassica juncea*, copy number variations of *MYB28* are responsible for aspects of glucosinolate biosynthesis, which determines seed oil quality (Yang et al. 2021). Similarly, variation in gene copy number involved in synthesis of benzylisoquinoline alkaloid matches with differences in alkaloid production in the opium poppy (Li et al. 2020). It is thus plausible that increases in CIN gene copy number from algae to vascular plants (Supplemental Fig. S3) are likely driven by increase in photosynthetic and respiration rates as reported in mosses, ferns, gymnosperm, and angiosperms. To this end, the net photosynthetic rates were only ∼0.0006 CO_2_  *μ*mol m^−2^ s^−1^, in mosses (*Distichophyllum freycinetii* and *Leucobryum* cf. *seemannii*), but increased to 8.7 CO_2_  *μ*mol m^−2^ s^−1^ and 10.3 CO_2_  *μ*mol m^−2^ s^−1^ in the angiosperms of *Populus tremuloides* and *Betula papyrifera*, respectively (Supplemental Table S6). A similar trend has been observed for respiration rates. Here, weak respiration (0.000102 CO_2_  *μ*mol m^−2^ s^−1^ and 0.000196 CO_2_  *μ*mol m^−2^ s^−1^) was observed in the mosses, which contrasts higher respiration rates (0.72 CO_2_  *μ*mol m^−2^ s^−1^ and 0.65 CO_2_  *μ*mol m^−2^ s^−1^) in the angiosperms (Supplemental Tables S6).

The subfamily of cytosolic CIN (β clade) expanded steadily in evolution until the emergency of early seed plants, the gymnosperm (Figs. 1 and S3 and Supplemental Tables S2 and S6), which corresponds the elevated rates of photosynthesis and respiration (Kragh et al. 2017, Supplemental Table S6), underpinning the increase in energy demand and growth rates (Brodribb et al. 2005). Cytosolic CINs are key enzymes for glycolysis and maintenance of sugar homeostasis (Stitt and Sonnewald 1995; Ruan et al. 2010; Wan et al. 2018), indicated from the severe growth phenotype observed in β CIN mutants (Barratt et al. 2009; Welham et al. 2009; Huang et al. 2022). Interestingly, our AP-MS analyses revealed that β clade CINs interacted with a cohort of proteins including sucrose phosphate synthase, proline biosynthesis enzyme, pyruvate kinase, phosphofructokinase, and aspartate aminotransferase (Fig. 6 and Supplemental Table S5), suggesting complex roles of cytosolic CINs in C and N metabolism. It is, thus, likely that the expansion of the β clade CINs have contributed to the enhancement in energy generation and use efficiency during evolution via modulating a range of C and N metabolic pathways.

It is noteworthy that following the duplication of plastidic CINs (α1) to form mitochondrial CINs (α2) in fern, the copy number of both α1 and α2 CINs doubled during the transition from gymnosperm to angiosperm characterized with about 30% to 36% rise in photosynthetic and respiration rates, whereas that of the cytosolic CINs remained unchanged (Supplemental Table S2 and Fig. S3). We propose that the increased number of α1 and 2 CINs in chloroplasts and mitochondria, respectively, may be important for ensuring high photosynthetic and respiration rates, which underpin high growth rate and biomass production in vascular plants, especially in angiosperm, as elaborated below.

### The mitochondria CINs were coevolved with vascular plants and may be important for aerobic respiration in plants

The transition from the aquatic to aerial environment marked the beginning of terrestrial colonization by plants and was underpinned by a dramatic increase in the efficiency of energy metabolism through a shift from anaerobic (e.g. those in cyanobacteria and algae) to aerobic respirations, which took place mainly in cytosol and mitochondria. It is estimated that a complete aerobic oxidation of 1 sucrose molecule through glycolysis in cytosol and TCA cycle in mitochondria produces 60 ATPs, which is 30-fold of that produced in anaerobic respiration. The coevolution of mitochondria CINs (α2) with vascular plants (Figs. 1, 2, and S3), coupled with evidence on their roles in mitochondrial function as shown through analyses of α2 CIN mutants (Xiang et al. 2011; Martín et al. 2013; Battaglia et al. 2017) indicate roles of α2 CINs in aerobic respirations. Interestingly, following the emergency of α2 CINs in fern, the cytosolic CIN (β clade) appeared stabilized in its copy number (Supplemental Fig. S3), which suggests a closer involvement of α2 CIN, than β CIN, in aerobic respiration.

The α2 CINs may contribute to mitochondrial function in several ways. Firstly, α2 CINs could enhance energy metabolism by providing glucose directly to mitochondrial hexokinase (HXK). A complete set of enzymes required for glycolysis have been found to be associated with mitochondrial membranes (Giegé et al. 2013). Having CINs in such a proximity with HXK in mitochondria could represent an efficient route from Suc to ATP generation, alternative to or in parallel with cytosolic glycolysis. Importantly, the proportion of mitochondria-associated glycolysis enzymes relative to that in cytosol increased in response to higher demand for respiration (Graham et al. 2007). The coevolution of mitochondria CINs with vascular plants may thus be part of the metabolic framework along with the mitochondria-associated glycolysis to support high respiration required for rapid growth in vascular and, in particular, seed plants (Supplemental Table S6). Secondly, having α2 CINs may also contribute to metabolic redundancy to ensure cellular energy security in case of malfunction in cytosolic Suc catabolism and glycolysis. Thirdly, α2 CINs may contribute to protection of mitochondria from oxidative damage. In this context, a knockout mitochondrial CIN, AT1G56560, induced expression of oxidative stress defense genes such as those encoding ascorbate peroxidase, leading to more severe growth phenotype than that of a knockout mutant for cytosolic CIN At1g355 (Xiang et al. 2011; Martín et al. 2013). It is likely that α2 CINs generate Glc to mitochondria-associated HXK, thereby contributing to the maintenance of ROS hemostasis (Xiang et al. 2011). Indeed, it has been shown that the loss of α2 CIN At3g05820 prevented ROS formation in roots (Battaglia et al. 2017). Consistent with these hypotheses, our affinity purification MS identified the lipoyltransferase 2 and thioredoxin M-type 1 interacted with α2 CINs, AtCIN1 (AT3G06500), and AtCIN2 (AT1G56560, Fig. 6). Along these lines, the doubling of α2 CIN copy number during the evolution of angiosperm from gymnosperm (Supplemental Fig. S3) may be required to keep ROS hemostasis as the former typically grow much faster than the latter and thus could generate excessive ROS through rapid metabolism.

### Plastidic CINs play roles in intracellular sugar partitioning and development

Although Suc biosynthesis and degradation pathways are biochemically well characterized, relatively little is known how Suc is partitioned among different subcellular compartments. The operation of CINs in mitochondria and plastids based on evidence of their evolution (Figs. 1 to 6), subcellular localization, and functional assay (Vargas et al. 2008; Tamoi et al. 2010; Xiang et al. 2011; Martín et al. 2013), strongly indicates the availability of Suc in these organelles. This view is further supported by data from, for example, nonaqueous fractionation assays on Arabidopsis leaves (Nägele and Heyer 2013) and the capacity of chloroplast and amyloplast from *Nicotiana benthamiana* and potato tuber, respectively, to synthesize fructan using Suc as a substrate and the reduction of total Suc level once a yeast invertase was introduced to the amyloplasts of potato tubers (Gerrits et al. 2001). Finally, the identification of plastidic sugar transporter (pSuT) from Arabidopsis that is located on the inner envelope membrane would support the movement of Suc and Glc from chloroplasts to cytosol as a H^+^-sugar antiporter (Patzke et al. 2019). Given the presence of Suc in the plastids and evident growth phenotypes of the plastidic CIN mutants (Vargas et al. 2008; Tamoi et al. 2010; Patzke et al. 2019), we propose that plastidic CIN (α1) may play major roles in maintaining Suc and hexose homeostasis within plastids and between plastids and cytosol. Such an intracellular balance would be vital for multiple cellular functions including the operation of plastidic oxidative pentose phosphate pathway, and in some species the synthesis of fructan that, together with Suc, protects cells from injuries caused by abiotic stress (Gerrits et al. 2001; Patzke et al. 2019). The maintenance on cytosolic and plastidic Suc and Glc levels is indeed essential for a wide range of physiological and developmental processes. For example, blockage of Suc and Glc export from chloroplasts to cytosol shown in the *pSuT* knockout mutant increased and decreased Suc level in the chloroplast and cytosol, respectively, which reduced branching and freezing tolerance and delayed flowering, probably through suppression of the expression of FLOWERING LOCUS T (FT) gene (Patzke et al. 2019). As such, it is plausible that the plastidic CIN and the pSuT may act in tandem as key players in modulating intracellular sugar homeostasis for metabolism, development, and adaption to stresses. Looking at the interacting proteins of the plastidic and the cytosolic CINs (Fig. 6), a couple of intriguing observations can be made. For example, the interactions with senescence-associated proteins and those involved in hormone and energy metabolism would suggest functions in these processes. Furthermore, given the prominence of Suc as a signaling molecule mediating changes in gene expression (Ruan 2014; Fernie et al. 2020), it is possible that the plastidial CIN, in concert with the pSuT, act(s) as a sugar buffer by sequestrating and recycling Suc.

In summary, our study indicates that the diversification of CIN gene family is likely a major molecular innovation underpinning efficient carbon and energy metabolism during evolution, essential for the rapid growth and colonization of land plants. Evidence supporting this conclusion includes (i) the steady expansion of cytosolic CIN (β clade) from algae to bryophyte and seed plants, (ii) the coevolution of the mitochondrial CIN (α2 clade), duplicated from the plastidial CIN (α1 clade), with vascular plants, and (iii) the expansion of mitochondrial and the plastidial CINs from gymnosperm to angiosperm species, which collectively matches the increase in energy metabolism efficiency and growth rates during evolution (e.g. Figs. 1, 7, and S3 and Supplemental Table S6). Further, the identification of CIN-interacting proteins support the role of α and β CINs in glycolysis, oxidative stress tolerance, and the maintenance of sugar homeostasis (Supplemental Table S5 and Fig. 6), which is consistent with the mitochondrial, plastidial, and growth phenotypes reported from studies on respective CIN mutants.

## Materials and methods

### Database searches and analyses

Hidden Markov model (HMM) profile of Glyco_hydro_100 domains (PF12899), which were conserved CIN motifs, downloaded from Pfam (http://pfam.sanger.ac.uk/) were employed to identify the putative neutral/alkaline invertase genes from different species from algae to seed plants (Supplemental Table S1). Genome assembly and annotation of these plant species were from JGI genome database. The BlastP search was performed using the HMM profile in the JGI genome database (http://www.phytozome.net/), followed by removal of redundant sequences. Sequences with an *E*-value over 10^−10^ and query cover 50% were chosen as candidate CINs. All redundant putative CIN sequences were excluded. The remaining CIN sequences were examined for Glyco_hydro_100 domains by the Pfam server. The corresponding DNA and protein sequences of CIN genes were downloaded.

### Sequence alignment and phylogenetic analysis

Multiple sequence alignment of all neutral/alkaline invertase protein sequences was conducted with the online version of MAFFT (v7.310; http://mafft.cbrc.jp/alignment/server/, last accessed December 21, 2021) (Katoh et al. 2002). The gap regions were deleted by TrimAl (1.2rev59) software (gap threshold set 0.7) (Capella-Gutiérrez et al. 2009), and the remaining 2,184 positions were used for molecular phylogenetic analysis of neutral/alkaline invertase proteins (Fig. 2). Systematic phylogenetic analysis of the CIN family was performed using ML method. First, phylogenies were reconstructed by Bayesian inference using MrBayes 3.1.2 with maximum likelihood (ML) using Phyml 3.0 and distance methods using MEGA X (www.megasoftware.net) (Kumar et al. 2018). A bootstrap test of 1,000 replicates was used to evaluate the reliability of internal branches. Second, the software ProtTest (version 2.4) was used for model selection, crucial for ML and Bayesian analysis (Abascal et al. 2005). The ML trees were constructed with the RAxML v8.2 (raxmlHPC-AVX-v8) software (Kozlov et al. 2019) with the Whelan and Goldman amino acid substitution model, g-distribution, and 100 nonparametric bootstrap replicates (Guindon and Gascuel 2003; Stamatakis 2006).

The sequences of CINs are from the following species: a charophyte, *K. nitens*, a moss, *P. patens*, and a lycophyte, *S. moellendorffii*, and 7 vascular plant species including rice (*O. sativa*), *lis*, sorghum (*Sorghum bicolor*), maize (*Zea mays*), Arabidopsis, grape (*Vitis vinifera*), *P. trichocarpa*, *Eucalyptus grandis*, and tomato (*Solanum lycopersicum*), *Anabaena* sp. PCC 7120 (InvA, WP_010995690.1; InvB, CAC85155.1), *Acidithiobacillus thiooxidans* ATCC19377 (Act-1, WP_010639088.1), *Acidihalobacter ferrooxidans* (Af-1, WP_076837606.1), *Acidihalobacter prosperus* (Ap-1, WP_065089328.1), *Cylindrospermopsis raciborskii* CS-505 (Cr-1, ZP_06306902.1), *Chroococcidiopsis thermalis* (Ct-1, WP_015156189.1), *Cyanothece* sp. PCC 7822 (Cy-1, WP_013325329.1), *Ectothiorhodospira* sp. PHS-1 (Ec-1, WP_083838783.1), *Ectothiorhodospira haloalkaliphila* (Eh-1, WP_026623645.1), *Fischerella* sp. JSC-11 (Fi-1, ZP_08987807.1), *Halothiobacillus* sp. LS2 (Ha-1, WP_066101158.1), *Halothiobacillus neapolitanus* c2 (Hn-1, WP_012823125.1), *Magnetofaba australis* IT-1 (Ma-1, WP_085442439.1), *Myxosarcina* sp. GI1 (My-1, WP_036482288.1; My-2, WP_036476871.1), *Nitrosomonas cryotolerans* (Nc-1, SFQ17342.1), *Nostoc punctiforme* ATCC 29133 (Np-1, CAD37134.1; Np-2, CAD37133.1), *Nodularia spumigena* CCY9414 (Ns-1, ZP_01631199.1), *Pleurocapsa* sp. PCC 7319 (Pl-1, WP_019507642.1), *Prochlorococcus marinus* (Pm-1, WP_011131014.1; Pm-2, WP_036892687.1), *Stanieria cyanosphaera* (Sc-1, WP_041619725.1; Sc-2, WP_015195523.1), *Synechococcus* sp. WH 8020 (Sy-8020-1, WP_048348470.1), *Synechococcus* sp. TMED187 (Sy-TMED187-2, OUW48797.1), *Synechocystis* sp. (PCC 6803, Syi-6803-1, CAD33848.1; PCC 7509, Syi-7509-2, WP_009632367.1; PCC 6714, Syi-6714-3, WP_028948477.1), *Thiohalorhabdus denitrificans* (Td-1, WP_054965954.1), *Thiohalospira halophila* DSM 15071 (Th-1, WP_093427069.1), *Thiohalomonas denitrificans* (Tmd-1, WP_092994335.1), *Thioalkalivibrio nitratireducens* (Tn-1, WP_043739311.1), *Thioalkalivibrio paradoxus* (Tp-1, WP_006747994), and *Thioalkalivibrio sulfidophilus* (Ts-1, WP_012637287.1; Ts-2, WP_026289890.1).

### Prediction of conserved motif structures and signal peptides

To investigate the diversity and structure of CIN genes in plants, their predicted amino acid sequences were subjected to domain and motif analyses. These genes were predicted individually using the MEME/Motif Alignment and Search Tool system (https://meme-suite.org/meme/tools/meme). Conservation of each motif among the CIN genes was characterized using WebLogo version 2.8.2 (http://weblogo.berkeley.edu/) using the default settings. The SignalP 6.0 server predicts the presence of signal peptides of these CIN proteins (https://services.healthtech.dtu.dk/services/SignalP-6.0/).

### Net charge of CIN proteins

The net charge of signal peptides of CIN proteins located in chloroplasts and mitochondria was calculated using online tools Prot pi (https://www.protpi.ch/Calculator/ProteinTool#Results).

### Affinity purification mass spectrometry

Arabidopsis plants were grown in a greenhouse for 2 to 3 wk under 12 h light and 12 h dark with around 150 *µ*mol m^−2^ light intensity for the agro-infiltration. AP-MS was conducted by expressing target proteins fused with a C-terminal GFP tag in Arabidopsis leaves using the established transformation protocol (Zhang et al 2020). Tandem GFP was used as a negative control. Expression and localization of the tagged proteins were evaluated by visualizing GFP fluorescence using confocal microscopy as previously described (Zhang et al. 2019). The transformed plant materials were collected 6 d following their transfer to the greenhouse. After grinding into a fine powder using a ball mill (MM301, Retch, Haan, Germany), proteins were extracted by mixing 2 g of material with 2 mL extraction buffer (25 mM Tris-HCl [pH7.5], 15 mM MgCl_2_, 5 mM EGTA, 1 mM dithiothreitol, and 1 mM phenylmethylsulfonyl fluoride). Following removal of cell debris by repeated centrifugation at 22,000 × *g*, 4 °C for 5 min, the supernatant was mixed with 25 *µ*L of GFP-Trap_A slurry (ChromoTek, Martinsried, Germany) equilibrated with extraction buffer and incubated for 1 h at 4 °C with rotation. The beads were collected by centrifugation at 3,000 × *g*, 4 °C for 3 min, and washed 3 times each with extraction buffer containing different concentrations of NaCl (0, 250, and 500 mM) (Zhang et al. 2019).

The proteins remaining on the beads were subsequently subjected to proteomics via in-solution digestion with LysC and trypsin, and the resulting peptides were purified (Wiśniewski et al. 2009). LC-MS/MS analysis was performed on a Q Exactive Plus (Thermo Fisher Scientific) machine. Quantitative analysis of MS/MS measurements was performed with the Progenesis IQ software (Nonlinear Dynamics, Newcastle, UK). Proteins were identified from spectra using Mascot (Matrix Science, London, UK). Mascot search parameters were set as follows: TAIR10 protein annotation, requirement for tryptic ends, 1 missed cleavage allowed, fixed modification: carbamidomethylation (cysteine), variable modification: oxidation (methionine), peptide mass tolerance = ±10 ppm, MS/MS tolerance = ±0.6 Da, allowed peptide charges of +2 and +3. A decoy database search was used to limit false discovery rates to 1% on the protein level. Peptide identifications below rank 1 or with a Mascot ion score below 25 were excluded. Mascot results were imported into Progenesis QI, quantitative peak area information extracted, and the results were exported for data plotting and statistical analysis. These intensities were filtered against experiment control and normalized using the spectral index using the algorithms embedded in the CRAPome website (Mellacheruvu et al. 2013). Finally, the possible interactions were scored as FC-A calculated by the SAINT algorithm (Choi et al. 2012; Mellacheruvu et al. 2013). All of the FC-A scores of the detected peptides are presented in Supplemental Table S2. Finally, the interaction pairs with an FC score above 4 were selected and analyzed by SUBA4 (Hooper et al. 2016) in order to restrict defined interactors to those proteins that are colocalized to the cytosol (Zhang et al. 2019).

### Accession numbers

Sequence data from this article can be found in the GenBank/EMBL data libraries under accession numbers listed in Supplemental Tables S1 to S3, S5, and S7 as well as in Supplemental Figs. S1 and S2.

## Supplementary Material

kiad401_Supplementary_Data

## Supplemental data

The following materials are available in the online version of this article.

**Supplemental Figure S1**. Evolutionary relationship of the CINs.

**Supplemental Figure S2**. Multiple sequence alignment of CIN proteins from α1 and β family in plant species tested.

**Supplemental Figure S3**. Variation of numbers of CINs in different plant species.

**Supplemental Table S1**. CIN proteins identified from 93 plant species.

**Supplemental Table S2**. CIN proteins identified from 14 plant species.

**Supplemental Table S3**. The charges of NTSs from different CIN proteins tested.

**Supplemental Table S4**. Major MEME motifs in predicted CIN proteins.

**Supplemental Table S5**. List of the interaction by affinity purification with fold change.

**Supplemental Table S6**. Net photosynthesis and dark respiration rates of diverse species from moss to angiosperm.

**Supplemental Table S7**. Protein interaction network annotation of Fig. 6.
