# Correction to: Evolution of cytosolic and organellar invertases empowered the colonization and thriving of land plants

**DOI:** 10.1093/plphys/kiae043

**Published:** 2024-02-19

This is a correction to: Hongjian Wan, Youjun Zhang, Limin Wu, Guozhi Zhou, Luzhao Pan, Alisdair R Fernie, Yong-Ling Ruan, Evolution of cytosolic and organellar invertases empowered the colonization and thriving of land plants, *Plant Physiology*, Volume 193, Issue 2, October 2023, Pages 1227–1243, https://doi.org/10.1093/plphys/kiad401

The authors wish to make the following amendments to the originally published version. The correction does not affect the interpretation of the data and the conclusion drawn in the paper.

1. In Figure 5, part of figure legend, “……. four additional chlorophytes were added, including *Chlamydomonas reinhardtii* (Cr), *Volvox carteri* (Vc), *Coccomyxa subellipsoidea* (Csu), and *Micromonas pusilla* (Mp)”, should be changed into “……two additional chlorophytes were added, including *Chlamydomonas reinhardtii* (Cr) and *Volvox carteri* (Vc).”
2. In Figure 7, Lycophytes were incorrectly listed under non-vascular plants. Now, we have re-labelled these as basal vascular plants, and made the corresponding change in the section on page 1234 “Coevolution of α2 CINs with vascular plants and differential expansion of CIN subfamilies”, where the sentence “….. a member of an ancient vascular plant lineage….. “ should be changed into “….. a member of a basal vascular plant lineage……”.
  The charophytes were incorrectly joined with moss and grouped as non-vascular plants in Figure 7. Both errors have been corrected in the revised Figure 7.
  The revised Figure 7 legend is : Origin and evolution of CIN gene family. Plant CINs originated from an orthologous ancestral gene after the endosymbiotic origin of chloroplasts. In charophytes, mosses and basal vascular plants, duplication of CINs (α1 clade) from chloroplast produced CINs in the cytosol with a loss of signal peptides from N-terminals (β clade). No α2 CINs were identified in *Selaginella moellendorff*, a basal vascular plant. In vascular plants, duplication of CINs (α1) from chloroplast produced CINs in the mitochondria (α2). (A) Endosymbiotic gene transfer. (B) The first gene duplication event resulted in the loss of signal peptides, hence the appearance of β CIN in the cytosol. C) Whole genome duplication. D) The second gene duplication event leads to the creation of CINs in the mitochondria (α2).
3. In supplemental Figure S3, *Selaginella modlendorff* was incorrectly grouped as ferns. In this correction, the word “Ferns” has been deleted from the legends on the X-axis. The legend of Supplemental Figure S3 should be changed as below:
  “Variation of numbers of CINs in different plant species. No α2 subfamily members were identified in *Selaginella moellendorff*, a basal vascular plant, however, one member of α2 CIN appeared in Pteridophyta”. The corrected Suppl Figure S3 is provided.

These errors have been corrected online.

