# Supplementary material for: Evolution of cytosolic and organellar invertases empowered the colonization and thriving of land plants
Source: Plant Physiol. 2023 Jul 11;193(2):1227–43. doi: 10.1093/plphys/kiad401 (PMC10661998; doi:10.1093/plphys/kiad401)
Supplement: kiad401_Supplementary_Data [file kiad401_supplementary_data.zip › Supplemental Figure S1S3.pdf]

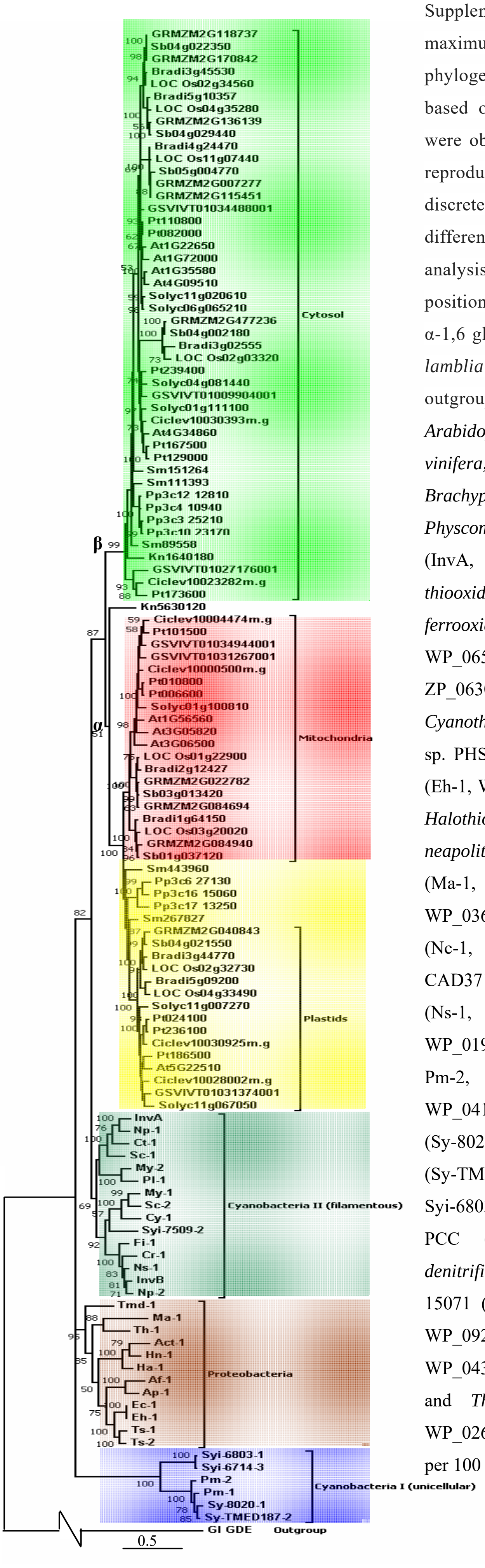

Supplemental Figure S1 Evolutionary relationship of the CINs. The maximum likelihood (ML) method was adopted to construct the phylogenetic tree. Bootstrap consensus tree inferred from ML analysis, based on the JTT matrix-based model. Bootstrap confidence values were obtained by 500 replicates. Branches corresponding to partitions reproduced in less than 50% bootstrap replicates are collapsed. A discrete Gamma distribution was used to model evolutionary rate differences among sites (5 categories (+G, parameter = 0.9923)). The analysis involved 121 amino acid sequences, with a total of 2184 positions. Evolutionary analyses were conducted in MEGA7.0. The α-1,6 glucosidase domain of glycogen debranching enzyme of *Giardia lamblia* ATCC50803 (Gl-GDE, XP_001705733.1) was used as the outgroup. The sequences of CINs are from the following species: *Arabidopsis thaliana, Citrus clementina, Populus trichocarpa, Vitis vinifera, Solanum lycopersicum, Zea mays, Sorghum bicolor, Brachypodium distachyon, Oryza sativa, Selaginella moellendorffii, Physcomitrium patens, Klebsormidium nitens, Anabaena* sp. PCC 7120 (InvA, WP_010995690.1; InvB, CAC85155.1), *Acidithiobacillus thiooxidans* ATCC19377 (Act-1, WP_010639088.1), *Acidihalobacter ferrooxidans* (Af-1, WP_076837606.1), *Acidihalobacter prosperus* (Ap-1, WP_065089328.1), *Cylindrospermopsis raciborskii* CS-505 (Cr-1, ZP_06306902.1), *Chroococcidiopsis thermalis* (Ct-1, WP_015156189.1), *Cyanothece* sp. PCC 7822 (Cy-1, WP_013325329.1), *Ectothiorhodospira* sp. PHS-1 (Ec-1, WP_083838783.1), *Ectothiorhodospira haloalkaliphila* (Eh-1, WP_026623645.1), *Fischerella* sp. JSC-11 (Fi-1, ZP_08987807.1), *Halothiobacillus* sp. LS2 (Ha-1, WP_066101158.1), *Halothiobacillus neapolitanus* c2 (Hn-1, WP_012823125.1), *Magnetofaba australis* IT-1 (Ma-1, WP_085442439.1), *Myxosarcina* sp. GI1 (My-1, WP_036482288.1; My-2,WP_036476871.1), *Nitrosomonas cryotolerans* (Nc-1, SFQ17342.1), *Nostoc punctiforme* ATCC 29133 (Np-1, CAD37134.1; Np-2, CAD37133.1), *Nodularia spumigena* CCY9414 (Ns-1, ZP_01631199.1), *Pleurocapsa* sp. PCC 7319 (Pl-1, WP_019507642.1), *Prochlorococcus marinus* (Pm-1, WP_011131014.1; Pm-2, WP_036892687.1), *Stanieria cyanosphaera* (Sc-1, WP_041619725.1; Sc-2, WP_015195523.1), *Synechococcus* sp. WH 8020 (Sy-8020-1, WP_048348470.1), *Synechococcus* sp. TMED187 (Sy-TMED187-2, OUW48797.1), *Synechocystis* sp. (PCC 6803, Syi-6803-1, CAD33848.1; PCC 7509, Syi-7509-2, WP_009632367.1; PCC 6714, Syi-6714-3, WP_028948477.1), *Thiohalorhabdus denitrificans* (Td-1, WP_054965954.1), *Thiohalospira halophila* DSM 15071 (Th-1, WP_093427069.1), *Thiohalomonas denitrificans* (Tmd-1, WP_092994335.1), *Thioalkalivibrio nitratireducens* (Tn-1, WP_043739311.1), *Thioalkalivibrio paradoxus* (Tp-1, WP_006747994) and *Thioalkalivibrio sulfidophilus* (Ts-1, WP_012637287.1; Ts-2, WP_026289890.1). The scale bar corresponds to a distance of 50 changes per 100 amino acid positions.

```
---------------------------------------------MSS--LDGDVSQN--------------------- Potri.004G167500
---------------------------------------------MSS--INVDVSLK--------------------- Potri.009G129000
---------------------------------------------MDVQ-RKGSVTEN--------------------- Eucgr.G01751.1
---------------------------------------------MSSFNLSVDVNQN--------------------- At4G34860
---------------------------------------------MSE--LSPKLGQN--------------------- GSVIVT01024105001
---------------------------------------------MST--HSGDVSNND-------------------- Solyc01g111100
---------------------------------------------MSSI-AT-DVSMN--------------------- Eucgr.F01661.1
---------------------------------------------MSPI-AAMDVCQN--------------------- Potri.005G239400
---------------------------------------------MSPI-PM-DVYSN--------------------- GSVIVT01009904001
---------------------------------------------MP---SPVDVSQN--------------------- Solyc04g081440
---------------------------------------------------MEG------------------------- At1G35580
---------------------------------------------------MEEGHKEP-------------------- At4G09510
----------------------------------------------------MEGP----------------------- Eucgr.F02588.1
----------------------------------------------------MDGSKEF-------------------- GSVIVT01034488001
----------------------------------------------------MDGTKEM-------------------- Potri.013G110800
----------------------------------------------------MDATKET-------------------- Potri.019G082000
------------------------------------------------------------------------------- Solyc11g020610
----------------------------------------------------MEET----------------------- Solyc06g065210
------------------------------------------------------------------------------- At1G22650
----------------------------------------------------MELAVG--------------------- GRMZM2G118737
----------------------------------------------------MELAVG--------------------- Sb04g022350
----------------------------------------------------MELAVG--------------------- GRMZM2G170842
------------------------------------------------------MELGA-------------------- Bradi3g45530
----------------------------------------------------MELAVGA-------------------- LOC_Os02g34560
--------------------------------------------------------MEA-------------------- GRMZM2G136139
--------------------------------------------------------MEA-------------------- Sb04g029440
--------------------------------------------------------MEV-------------------- LOC_Os04g35280
--------------------------------------------------------MEA-------------------- Bradi5g10357
------------------------------------------------------------------------------- At1G72000
------------------------------------------------------------------------------- GRMZM2G007277
------------------------------------------------------------------------------- Sb05g004770
------------------------------------------------------------------------------- GRMZM2G115451
------------------------------------------------------------------------------- LOC_Os11g07440
------------------------------------------------------------------------------- Bradi4g24470
---------------------------------------------MVGVSTQGPETPLD-------------------- TnS000892793t06
--------------------------------------------------MPFDMNSFQ-------------------- Pp3c4_10940
------------------------------------------------------MNSPQ-------------------- Pp3c12_12810
--------------------------------------------------MPTNMTSFA-------------------- Pp3c3_25210
------------------------------------------------------MNIFG-------------------- Pp3c10_23170
--------------------------------------------------MPLGFDASA-------------------- Sm89558
------------------------------------------------------------------------------- kfl00164_0180_v1.1
--------------------------------------------------MPSTTTTMI-------------------- TnS000602483t02
---------------------------------------------------------ME-------------------- TnS000105403t01
----------------------------------------------------MVPRSLG-------------------- TnS000982973t03
------------------------------------------------------------------------------- Sm111393
```

β (cytosolic)

N-terminal sequences

```
--------------------------------------------MPPTPDKTSP-------------------------- Azfi_s0064.g035495
--------------------------------------------MAPS-QLTTT-------------------------- Azfi_s0040.g026676
-------------------------------------------------------------------------------- Sm151264
--------------------------------MQAATD--MFSVTPLSIDLLPEPSPDS--------------------- Azfi_s0393.g067806
------------------------------MPAAAATTNKTFNLGPLAVDLQSEYPST---------------------- Azfi_s0003.g008094
---------------------------------------MDIHSILNH----DVLQQGF--------------------- Azfi_s0059.g034661
---------------------------------------MPSNAVMNHKTQSDSLENGHGCADIINGSTDKPFKHNIETK Azfi_s0001.g000547
---------------------------------------MDRHHLLPK----SSLDSDF--------------------- Azfi_s0096.g043780
-------------------------------------------------------------------------------- GSVIVT01037429001
MHESLGEPLPDETE------PNPAAPPKSSTAG--ADQKSPPEPVPVAGVGGEGVPSASPENAGSLAPLPDP-------- Eucgr.D02386.1
MSTTLRDLLVAERENEASPPPFPGPSTRYSVSGDISDEEDSTNTSPLFSSEEEQSPENLKKVTAPAAYLLDLKLNENLDK Potri.002G173600
----MPEKKPTSSD--------SASTLDAKKLNLLPAVKYLLKELDLEHVDLDNLPQMVEAESYSYPDVTET-------- GSVIVT01027176001
---------------------------------------------------MVQCTQPP--------------------- GRMZM2G477236
---------------------------------------------------MVQCTQPP--------------------- Sb04g002180
---------------------------------------------------MVLWAEPI--------------------- Bradi3g02560
---------------------------------------------------MVLYADPP--------------------- LOC_Os02g03320
-------------------------------------------------------------------------------- At5G22510
-------------------------------------------------------------------------------- Eucgr.J00457.1
-------------------------------------------------------------------------------- Potri.008G024100
-------------------------------------------------------------------------------- Potri.010G236100
-------------------------------------------------------------------------------- GSVIVT01034753001
-------------------------------------------------------------------------------- Eucgr.G02704.1
-------------------------------------------------------------------------------- GSVIVT01031374001
-------------------------------------------------------------------------------- Potri.004G186500
-------------------------------------------------------------------------------- Solyc11g007270
-------------------------------------------------------------------------------- GRMZM2G040843
-------------------------------------------------------------------------------- Sb04g021550
-------------------------------------------------------------------------------- Bradi3g44770
-------------------------------------------------------------------------------- LOC_Os02g32730
-------------------------------------------------------------------------------- Bradi5g09200
-------------------------------------------------------------------------------- LOC_Os04g33490
-------------------------------------------------------------------------------- Solyc11g067050
-------------------------------------------------------------------------------- TnS000875687t08
-------------------------------------------------------------------------------- Sm267827
-------------------------------------------------------------------------------- Azfi_s0345.g065806
-------------------------------------------------------------------------------- Pp3c6_27130
-------------------------------------------------------------------------------- Pp3c16_15060
-------------------------------------------------------------------------------- Pp3c17_13250
-------------------------------------------------------------------------------- Sm443960
-------------------------------------------------------------------------------- kfl00563_0120_v1.1
```

α1 (plastidic)

N-terminal sequences

```
------------------GSLKSVDAHP-----------ALA----EIEDLDFSRILDKPPR----------------- Potri.004G167500
------------------GSLRNAETLC-----------DMA----EIEEMDFSRIFDRPPR----------------- Potri.009G129000
------------------GNVRGSDLLC-----------TLA----ESEECDFSKLMERP-R----------------- Eucgr.G01751.1
------------------GNIKNVDSLS-----------TLD----DIDDIDFAKLLEKP-R----------------- At4G34860
------------------GTIKNIDSSS-----------TVA----ETEDIDFSKLSERP-R----------------- GSVIVT01024105001
------------------ASIRNIDSCS-----------TVT----ELDDIDFSRLP-RP-R----------------- Solyc01g111100
------------------GSMGNHDTTN-----------SIF----EIDDSDILKLMDRP-R----------------- Eucgr.F01661.1
------------------ASVKNFEAAG-----------SIF----EID-SEFLRLSDKP-R----------------- Potri.005G239400
------------------GNVKNLETAS-----------TTV----QIDDSDFLRLLDRP-R----------------- GSVIVT01009904001
------------------GNARHAEAAP-----------SLF----EIE-EDLARLLERP-R----------------- Solyc04g081440
------------------VGLRAVGSHC-----------SLS----EMDDLDLTRALDKPR------------------ At1G35580
------------------LVLRVEGSHC-----------SLS----EMDDFDLTRALEKPR------------------ At4G09510
-------------------GMRKVSSHC-----------SIN----DMFDEDFSRLLEKPR------------------ Eucgr.F02588.1
-------------------GLKNVSSHC-----------SIS----EMADYDLSRLLDKPR------------------ GSVIVT01034488001
------------------GGLRNVSSVC-----------SIS----EMDDFDLSRLLDKPK------------------ Potri.013G110800
------------------VGLMNGSSVW-----------SIS----EMDDIDFSRLSDKPK------------------ Potri.019G082000
-----------------------------------------------MDDFDLSKLLDKPR------------------ Solyc11g020610
-------------------GLRNVGSNC-----------SIS----EIDDYDLSKLLNKPR------------------ Solyc06g065210
--------------------MEGVNSSS-----------SIS----DLD--ELARLLDRPR------------------ At1G22650
------------------GGMRRSASHT-----------SLS----ESDDFELTRLLS-KP------------------ GRMZM2G118737
------------------GGMRRSASHT-----------SLS----ESDDFELTRLLS-KP------------------ Sb04g022350
------------------GGMRRSASHT-----------SLS----ESDDFELTRLLS-KP------------------ GRMZM2G170842
------------------GGMRRSASHT-----------SLS----ESDDFDLSRLLN-KP------------------ Bradi3g45530
------------------GGMRRSASHT-----------SLS----ESDDFDLSRLLN-KP------------------ LOC_Os02g34560
------------------APMRKASSQA-----------SLA----DPDDFDLTRLLNHKP------------------ GRMZM2G136139
------------------AAMRKASSQA-----------SLA----DPDDFDLTRLLNHKP------------------ Sb04g029440
------------------AGMRKASSHA-----------SMAAAAADPDDFDLTRMLNHRP------------------ LOC_Os04g35280
------------------AAAATTTNKAP----------SMAD---PEDDFDLSRLLNHRP------------------ Bradi5g10357
------------------------------------------------------------------------------- At1G72000
--------------------MKRVSSHVS----------LAS---EAEINLDLSRLIIDKP------------------ GRMZM2G007277
--------------------MKRVSSHVS----------MAS---EAEINLDLSRLIIDRPQ----------------- Sb05g004770
--------------------MKRVSSHVS----------LAS---EAEINLDLSRLVIDKP------------------ GRMZM2G115451
--------------------MKRVSSHVS----------IAS---EAEINLDLSRLLIDKP------------------ LOC_Os11g07440
--------------------MKRVSSHVS----------IAS---EAEINLDLSRLLIDKP------------------ Bradi4g24470
------------------AAMKASDHHAA----------IEN------GSVDFSKLNERPR------------------ TnS000892793t06
------------------RHLSLETSSESLK------RTNDSYTSGDNGSTEFERPMESRP------------------ Pp3c4_10940
------------------RPLTLETSWESLR------RTIDSNP-VDNGSIDFERPMEVRP------------------ Pp3c12_12810
------------------RTLTLETN-------------NDSYASGDNGNVEFGRLMDLKP------------------ Pp3c3_25210
------------------KALSGETN-------------NDIYDSGENGSIDYGRLADFRP------------------ Pp3c10_23170
------------------ADPSDTAS-----------RNGDAHLCDLISDIDFSKLVALKP------------------ Sm89558
------------------------------------------------------------------------------- kfl00164_0180_v1.1
------------------DSLALDAHDEAAAAAALGLGLRNSMMMMMESRPRPLNVARRRP------------------ TnS000602483t02
------------------NSQSTDSG-------------TDQPKACSFFLYEGERPEQVRV------------------ TnS000105403t01
------------------RDAPTSSK-------------LQSASACDIFSSFLSLNLSLRS------------------ TnS000982973t03
------------------------------------------------------------------------------- Sm111393
```

β (cytosolic)

N-terminal sequences

```
--------------------IAAPLSPDVDFFFPMDIPPHALGIASKS-EKGLSRSKSRSSK------------------ Azfi_s0064.g035495
--------------------LGHLANDENEHHDGQEASPPTFHLALAEFDRDEEVAVAASSS------------------ Azfi_s0040.g026676
-------------------------------------------------------------------------------- Sm151264
------------------IILHPQIAKESSDASEHQQYPLFQQQQQQQSQSQSHSQARNGPP------------------ Azfi_s0393.g067806
---------------------PAARSNESDQTGAAGQVPRVPRPVADNHNLNSHYSFLASS------------------- Azfi_s0003.g008094
--------------------LNINDASSSI---------IPDQNGQTSDPNSNKQPVESS-------------------- Azfi_s0059.g034661
PRLTTSGSCEEKTLVEALEGFNINTCSNAI---------NSSKDQNENDNSSKDKNHESSEV------------------ Azfi_s0001.g000547
-----------------IKVFNNNNNININ---------NNDNDDKNGDENGEENGLINEVN------------------ Azfi_s0096.g043780
--------------------MAEDQSVVT------------------NENFDSSEANDDK-------------------- GSVIVT01037429001
------------APAVATSAATAAS-PGPRDTEAALKSRTES---GEIETPEQNRDSSDSAKD----------------- Eucgr.D02386.1
EELKKSSEFITLEPATIVDKSFEESEPSYSSVKEVLKTAEKSPFSSDLKCKQLSEVSSDLKIESVTLTDLTKEPSASLEK Potri.002G173600
------------SEKPLDSPAEASGEKNEQSTENVSGGSGRS-------VPTLKESHS---------------------- GSVIVT01027176001
------------------PQLKLPESKIT----------EPT----DDENQDLPPKPEKRTR------------------ GRMZM2G477236
------------------PQLKLPESKIT----------ELT----DDENHDSPPKLEKRTR------------------ Sb04g002180
------------------PPIVVPGSKIA----------ELT----NDSKHDTS-NLDQKTR------------------ Bradi3g02560
------------------PPIKPPEIKIK----------ELA----NKSTYESS-MMEQKTR------------------ LOC_Os02g03320
-------------MAASETVLRVPLGSVSQSCYLASFFVNSTPNLSFKPVS--RNRKTVRCTNSHEVSSVPKHS--FHSS At5G22510
-----------MAMASPAGCLNTLSGVPHSLHYRLSFLNYVSPVFAFSRDANYGQIRDSKHMHWYKTSTMLKHQPRVHSI Eucgr.J00457.1
-------------MATSDAVLQVLSGAGPRSFSSDLCFNNLDLAFRSK-HIKYVKKRASRHMKMLECSSVQQNCIGKHWF Potri.008G024100
-------------MATTEAILQVLSGAGPCVFSSDPCFRSSDLTFSSKLHIKRVKKRASRCMKMFECSNVLQNGIGNHWF Potri.010G236100
-----------MAMGTSEAVLQVFSGAVPCLFGSDPCFSKSDSMSPFKSHIKSVKKRGSR--YMLKCSYMIRSHIMTHRL GSVIVT01034753001
-------------MATAEGVLQVFSGVAPRNLCSDPSSNALGLAYSFNSRLVSTKRSASKKIKIPVYASIQSSTSETRGV Eucgr.G02704.1
-------------MGTSEAVLPSLSTAVPHLSHSKPCLNSLNSMLHLKSGINSRRKRALGYMRLLNCSRMLRNCRRVYSI GSVIVT01031374001
-------------MATSKTVLQVLSGGLPCPHRFDLSFGGLNSVLSICSDVKRRKNRGLVYKKLNNGMRLLGKCRS---- Potri.004G186500
-------------MATSEAFLQVLGGSLPSLFGSDDSFRKLGSSHTSRSFIRIRKKRGPICVNFLNCSHISYRAIRVDCF Solyc11g007270
-------------MGIAVVALHTM----PGAFTTHSPASILSLRPDFT--RINSRTNSVPNARTLQGVLRIPRLR----- GRMZM2G040843
-------------MGIAEVALHTM----PGAFTTHSPASILSLRAVAR--RRNKNTDSVPNTRALQGLLRIPRLR----- Sb04g021550
-------------MGIAEVALHTM----PGTFTGHSPASSLFLRTDAG-GRRKRNTNLFY--RKFKGTPKFPGLR----- Bradi3g44770
-------------MGIAEVALHSM----PGAFAAHSPASNLPLAADAARGRRRRSANSLHSSRALQGPVRFPGLRA---- LOC_Os02g32730
-------------MGIAGRVTAPL----PLRLGFPGVAPPRSAAHPRK-GGWGRKRN-PPCAANSLHPSNNNPRA----- Bradi5g09200
-------------MEIA-RVVAP-----PLRL----------AWCPAS-RRWGRRKGRPPCPSSLPGP------------ LOC_Os04g33490
-------------MGASEAALQLLSGELSCQVRTSSILAKSNSLLCYERCFKARNYGDWRYKQINSIKKLQDCSSLHAFH Solyc11g067050
--------------MMQALLLPAVPVGVCLPLGQINGFARLSMTTMRMRRRRRRRKCACVRSCLDGGAGAG--------- TnS000875687t08
----MHALQPRHSCGCGHTAIATQGSSAIGHWNWKRSRVRYEFRKKVYGVVRLRHCIARASSTASGSSSGSEEDH----- Sm267827
------------------------------------------MKEEFFSMKAKASVNEDIPSSHNGSSEES--------- Azfi_s0345.g065806
-------------MSSTFQLVDVLAAPRCR-----HLSSRIHLPKHVVSASLRPCLRGLSSLEER-TWTCKLREKALRT- Pp3c6_27130
-------------MNSTLRLVDVSAPLGCRQCAYFHRNVGAGEASCRSDGSISASFCGLSWRQQRGLNAWTLRSSCARV- Pp3c16_15060
-------------MKSTLRLVEPLAASGCHG------AASVSSAQRKVGDSWGYSPKRVAFHKQGRSYTCIKCEQNAGD- Pp3c17_13250
---------------MSPGSFPGVEAALSSPSRLKTWNPGSQSHGRVCRGSIIVSGLALSHPRRSDSIRFLRRGKWRRS- Sm443960
-------------------------------------------------------------------------------- kfl00563_0120_v1.1
```

α1 (plastidic)

N-terminal sequences

```
---------------------PLNMERQRSCDERS-LNEL-FGVPLLSPRPSSR--------------------AESNFR Potri.004G167500
---------------------PLNMDRQRSCDERS-LSELSTGLPIPSPRPSSR--------------------VENNFR Potri.009G129000
---------------------PLNMERKRSLDERS-LNELSTALSPHLSLRNS----------------------ESSSR Eucgr.G01751.1
---------------------PLNIDRLRSLDERS-LTELTGSPQLRN--------------------------ADNASR At4G34860
---------------------PLTMERQRSYDERSFLSELSVGMSPRLSIRN----------------------IDSYSR GSVIVT01024105001
---------------------NLNIERQGSYDEKS-LTETQLGFSPHPPSR-----------------------AENFFR Solyc01g111100
---------------------PVNVERKRSFDEKS-YSEMSITLSPRPSYR-----------------------MADSFH Eucgr.F01661.1
---------------------PVNVERKRSFDERS-FSEN-------------------------------------SFR Potri.005G239400
---------------------PISIERNRSFEEKS-FNELSSTLSPLLFHRNV---------------------EKNSFH GSVIVT01009904001
---------------------QVNIERKRSFDERS-FSEMSMTHSPPRQVYKN---------------------SENSSR Solyc04g081440
----------------------LKIERKRSFDERS-MSELSTGYSRH--------------------------------- At1G35580
---------------------QLKIERKRSFDERS-MSELSTGYVRQ--------------------------------- At4G09510
----------------------LNIERQRSFDERS-LSELSIGLTRVG-------------------------------- Eucgr.F02588.1
----------------------LNIERQRSFDERS-MSELSIGLARH--------------------------------- GSVIVT01034488001
----------------------LNIERQRSFDERS-LSELSIGLARG--------------------------------- Potri.013G110800
----------------------LNIERKRSFDERS-LSELSIGLARG--------------------------------- Potri.019G082000
----------------------INIERQRSFDERS-LSELSIGLSRG--------------------------------- Solyc11g020610
----------------------LNIERKRSFDERS-LSELSIGLSRG--------------------------------- Solyc06g065210
----------------------VNIERKRSFDERS-FSEMGI-------------------------------------- At1G22650
---------------------RINVERQRSFDDRS-LSDVSHSGG----------------------------------Y GRMZM2G118737
---------------------RINVERQRSFDDRS-LSDVSHSGG----------------------------------Y Sb04g022350
---------------------RINVERQRSFDDHS-LSDVSHSGG----------------------------------Y GRMZM2G170842
---------------------RINVERQRSFDDRS-LSDVSYSGG--------------------------------HAR Bradi3g45530
---------------------RINVERQRSFDDRS-LSDVSYSGGG-------------------------------HGG LOC_Os02g34560
---------------------RINVDRQRSFDDRS-LGEISLAGAG--TASRGG--------------------WGYGGG GRMZM2G136139
---------------------RINVERQRSFDDRS-LGELSLAGAGAGTGSRGG--------------------WGYYGV Sb04g029440
---------------------RINVDRQRSFDDRS-LAELSISGTA--SRGGGG--------------------GGYP-A LOC_Os04g35280
---------------------RINVERQRSFDDRS-LGDLYLSAMD-----------------------------GRSGG Bradi5g10357
---------------------------------MS-LS------------------------------------------ At1G72000
---------------------RFTLERKRSFDEQS-WSELSH-------------------------------------R GRMZM2G007277
---------------------RFTLERKRSFDEQS-WSELSHSHSH---------------------------------R Sb05g004770
---------------------RFTLERKRSFDEQS-WSELSH-------------------------------------R GRMZM2G115451
---------------------RLTLERKRSFDEQS-WSELSH-------------------------------------R LOC_Os11g07440
---------------------RFTLERKRSFDEQS-WSELSH-------------------------------------R Bradi4g24470
---------------------PLNIERQRSFDERS-LSEISAMSPHVHPR-----------------------------T TnS000892793t06
----------------------RQIETHHSLDERSLNDIISSGLSS--------------------------------PR Pp3c4_10940
----------------------RQIENHHSLDERSLNDIISSGLSS--------------------------------PR Pp3c12_12810
----------------------RQIETQLSLDERSINDIISSGLSS--------------------------------PR Pp3c3_25210
----------------------RKIDTYNSLDERSLNDIISSGLSS--------------------------------PR Pp3c10_23170
----------------------RPIETQRSLDERTLSDLVSPGIS---------------------------------PL Sm89558
-------------------------------------------------------------------------------- kfl00164_0180_v1.1
----------------------SMTSSSSSLDLMMEDDAEAGPVPT--------------------------------VK TnS000602483t02
----------------------EQRLSPDEKSLTELFCSNGTMISP--------------------------------RP TnS000105403t01
----------------------VAFAFGCVLGSWQQKEEEEEEVDY--------------------------------YL TnS000982973t03
```

β (cytosolic)

N-terminal sequences

```
-------------------------------------------------------------------------------- Sm111393
---------------------AVPRINSRPITVTDFLEALHIDTP-----------------------------FVRTPS Azfi_s0064.g035495
---------------------SCSSLG---LVLTPRRLRLHVRR-------------------------------LNSES Azfi_s0040.g026676
-------------------------------------------------------------------------------- Sm151264
---------------------PPPPPSRKSSVFESFSAAAAPARSEDVAAVVFFRSEAEERPLSDFAPSPRPCRTLETVR Azfi_s0393.g067806
--------------------------DHNHHGHHNYSSNNNSNNS----------NSNEQRSSS----SPR----LVPVR Azfi_s0003.g008094
------------------------INVTKPRLLTQLSTHRSCEEKTLVNESHN------------------VGGLSSRSR Azfi_s0059.g034661
---------------------SVKALETKPRLLP-LNTYRSCEEKTLLEA---------------------FGGLSTRSR Azfi_s0001.g000547
---------------------KVVWDSCTSGTPSSANTQRSYEVEELIVVD------------------------HGYSR Azfi_s0096.g043780
--------------------------CTRTLDAAK--------------------------------------------- GSVIVT01037429001
-------------------GDAEAMEKPSGVVEGEEEGS--PEKPSRKASFG-------------------------HDI Eucgr.D02386.1
VIAK-------SVEKCEAKGEATVSEESTVITSREVVGGRIPEKDEATVTVQSN-----------------------VCV Potri.002G173600
--------------------VGASLERYDQIR--EDKGVKKTDEPEKPLVFR-------------------------SEV GSVIVT01027176001
---------------------MHHIERHRSCVVTLSDIELNGLQPRHL-------------------------------- GRMZM2G477236
---------------------MHHIERHRSCVVTLSDIELNGLQPRRL-------------------------------- Sb04g002180
---------------------MRTIERHRSCEV--SQLILSEVEHDG--------------------------------- Bradi3g02560
---------------------LHAIERHRSCEV--SQAILSEVENRHQ-------------------------------- LOC_Os02g03320
NSV---------LKGKKFVSTICKCQKHD------VEESIRSTLLPS------------D---------GLSSELKSDLD At5G22510
PSVG--CTPWGYSELIRSHSLICKCQRTESNSGSSVESGTKSEVSLNGM----------N---------GQSTVEESEAV Eucgr.J00457.1
KRSGDGDLSVNAT-IKRLQLLRCKCQKAERVSGVTTEGGNGTWFVDSAKTLN-LNGAVN-----------TPGVLELGDT Potri.008G024100
KGLGDRDRSVNAT-INRLQLLRCKGPQAERVSGVT-EGGNGTWFVDGANTLN-QNGAVTG---------EHTDCFGAWDA Potri.010G236100
HGVG-GGLYGNTS-IHRSQLQSCKCQRADSVSGIASEAGNGTWFVDNAKKRNPINGVMD-----------TPNVLEFQDV GSVIVT01034753001
EGFG-GSSYNNRR-IDRLKFSSCRCQRAEGVGGTT----TGNWFVDSADDLNPLNTMVN-----------GPHVLNFDDV Eucgr.G02704.1
QGID-GFSHGKTK-ISRLESVSCKGQQAESVSGITAEDGHGTIIA--------------------------PKIKEFEMV GSVIVT01031374001
RGVGAVTSRGKVKCIDRWESMRCKCQKAESFGGATANEWSPVSLP----------------------------VNGVHGA Potri.004G186500
QSTR-QCVHGDIG-HSNLRSVNCKCQQADSASSFASEKGNGSWTIDNDQSFDTVHGN-------------TPSVMQFETV Solyc11g007270
-------------------SVRRLCQRIDD--RVTE--GNGPWVKDAMNN--------------------ASQVLGDISV GRMZM2G040843
-------------------SVRRLCQRIDDIARVTE--GNGTWVKEAMNN--------------------AGQVLGDISV Sb04g021550
-------------------SVERQCQRIDDLAKVIEA-GNGTWDKDVVNK--------------------ASQVLGDVSV Bradi3g44770
-------------------AVECQCQRIDDLARVTE--GNGAWVKDAVDK--------------------ASHALGDVRV LOC_Os02g32730
-------------------------RHHDPSLKVPG-----------------------------------GDVDG-MGV Bradi5g09200
--------------------------RHDHAVKRPG-----------------------------------GEAAGGRSV LOC_Os04g33490
GLHS---VFCGEKLLSQSNLLICNCQQPERVSETIIKGGNGKSMHTVSPK--------------------IPNLAPDEQN Solyc11g067050
---------------------AMDFMAAAATAATER------------------------------------NVLIEKMV TnS000875687t08
---------------------PLPAPDGEAIAGKRKKEGSDLTLVVS-----------------------VGENLRPGHV Sm267827
-------------------------------------------------------------------------------V Azfi_s0345.g065806
--------------TKRERYTSASDGHTC-IRCEQN-LAEQGRLLEF-INAH------------------NVTSGLSVEA Pp3c6_27130
--------------VKR-RWR---DRQTCGIHCELKNVAEQECLLD------------------------NESILANRKV Pp3c16_15060
--------------SSRVVYNNQAHLEREALQEKVVVSSLQGATPEDSICVD------------------TKSSSRREDV Pp3c17_13250
-------------------PLKSRLYILAENRVFTQDGANPVPDLDQQRQ--------------------RQVYVPAAGI Sm443960
--------------------------------------------------------------------------MSEPEI kfl00563_0120_v1.1
```

α1 (plastidic)

N-terminal sequences

```
LIDHLDGLYSP-----------GRRS---GFNTPRSQYGFE--------THPAVAEAWDALRRSLVVFRGQPVGTIAALD Potri.004G167500
LIDHLNCLPSP-----------GRRS---GFNTPLSQFGVE--------THPTVAEAWEALRRSLVYFRGEPVGTIAALD Potri.009G129000
LTDPFGSFLSP-----------DRRS---GYNTPRADNGFD--------THPMVAEAWEALRRSLVYFRGQPVGTIAALD Eucgr.G01751.1
APDHADYVISPSF---------GRRS---GFNTPRSQPGFE--------SHPMVGEAWDALRRSMVYFRGQPVGTIAAVD At4G34860
NIDHLDTVFSP-----------CRRS---GFNTPRSAMDFE--------PHPMFAEAWEGLRRSLVFFRGKPVGTIAALD GSVIVT01024105001
ALEHFDCIFSP-----------SKRS---EFTTPRSPFGQG--------PHPMVAEAWDSLRRTLVHFRGQPVGTIAALD Solyc01g111100
MLDHHEYVYSPG-----------RRS---GFHTPRSESYYE--------TLPMIADAWEALRRSVVHFRGQPVGTIAALD Eucgr.F01661.1
IIDHLENLSPAG-----------RRS---GFNTPRS-CGFE--------SHPMVVDAWESLRRTLVYFRSQPVGTIAALD Potri.005G239400
IFDLLDHTFSP------------VRS---SLNTPRSNHCFE--------PHPVFTDAWEALRRSLVYFRGQPVGTIAAID GSVIVT01009904001
VFDNMVGVYSPG-----------RWS---GIHTPRSTFGYE--------PHPIIGEAWEALRRSIVNFRDQPVGTIAAID Solyc04g081440
-DGIHD---SPRG-----------RS-VLDTPLSSARNSFE--------PHPMMAEAWEALRRSMVFFRGQPVGTLAAVD At1G35580
-DSILEMAHSPGS-----------RS-MVDTPLS-VRNSFE--------PHPMVAEAWEALRRSMVFFRGQPVGTIAAYD At4G09510
-LDNLDSTYSPGR-----------RS-GFDTPASSARNSFE--------PHPMVADAWEALRKSLVYFRGQPVGTIAAVD Eucgr.F02588.1
-LEHLDSMYSPGG-----------RS-GFDTPASSARNSFE--------PHPMVNEAWEALRRSLVFFRGQPVGTIAAYD GSVIVT01034488001
-IDTFETTYSPGG-----------RS-GFNTPASSTRNSFE--------PHPMVADAWEALRRSLVYFRGQPVGTIAAYD Potri.013G110800
-IDNFETTNSPGG-----------RS-GFNTPASSARNSFE--------PHPMVADAWEALRRSLVFFRGQPVGTIAAYD Potri.019G082000
-LDNYENAYSPG------------RS-GLDTPASSARNSFE--------PHPMVAEAWDALRRSMVHFRGQPVGTIAAVD Solyc11g020610
-LDHYESAS-PG------------QS-VLDTPVSSARNSFE--------PHPMVAEAWDALRRTMVHFRGQPVGTIAAID Solyc06g065210
----FDNVNSPG---------------GWETPVSSARNSFE--------PHPMVAEAWDALRRSLVYFRGQPVGTIAAYD At1G22650
GRGGFDGMYSPGG---------GLRS-LVGTPASSGLHSFE--------PHPIVGDAWEALRRSLVLFRGQPLGTVAAVD GRMZM2G118737
GRGGFDGMYSPGG---------GLRS-LVGTPASSALHSFE--------PHPIVGDAWEALRRSLVFFRGQPLGTVAAVD Sb04g022350
GRGGFDGMYSPGG---------GLRS-LVGTPASSALHSFE--------PHPIVGDAWEALRRSLVFFRGQPLGTVAAVD GRMZM2G170842
GGGGFDGMYSPGG---------GLRS-LVGTPASSALHSFE--------PHPIVGDAWEALRRSLVFFRGQPLGTIAAYD Bradi3g45530
TRGGFDGMYSPGG---------GLRS-LVGTPASSALHSFE--------PHPIVGDAWEALRRSLVFFRGQPLGTIAAFD LOC_Os02g34560
GMESYESMYSPGG---------GLRS-YCGTPASSTRLSFE--------PHPLIGEAWDALRRSMVSFRGQPLGTIAAVD GRMZM2G136139
GVESYESMYSPGG---------GLRS-YCGTPASSTRLSFE--------PHPLIGEAWDALRRSIVSFRDQPIGTIAAVD Sb04g029440
MMESYESMYSPGG---------GLRS-LCGTPASSTRLSFD--------PHPLVFDAWDALRRSLVCFRGQPLGTIAAVD LOC_Os04g35280
YMDSYDTMYSPGG---------GLRS-LSGTPASSTRHSFE--------PYPLVFEAWEALRRSLVCFRGQPLGTIAAVD Bradi5g10357
---LYDSAHSLDG-----------KS-GWDTPVFSMKDSMD--------RNPMVTEAWEALCQSQVYFRGKPVGTIAAYD At1G72000
QNDGFDSVLQSPA---------FPSG-VFDSPFSVGTHFGG--------PHPLVNEAWEALRKSVVYFREQPVGTIAAVD GRMZM2G007277
NNDGFDSVLQSPA---------FPSAGGFDSPFSIGTHFGGG------GPHPLVNEAWEALRKSVVYFREQPVGTIAAVD Sb05g004770
QNDGFDSVLQSPA---------FPSG-GFDSPFSVGTHFGG--------PHPLVNEAWEALRKSVVYFREQPVGTIAAVD GRMZM2G115451
QNDGFDSIMHSPA---------FPSG--FDSPFSLGT-LGD--------PHPLVNEAWEALRKSVVYFRGQPVGTIAAVD LOC_Os11g07440
PNDGFDSVMHSPA---------FPTG--LDSPFSMGTHFGEPS-----GPHPLVNEAWEALRKSVVYFRGQPVGTIAAVD Bradi4g24470
SETHFEGMLSPG-----------RRS-VFDTPKS-SQENFE--------PHPMVYDAWETLRKSLVYFRGKPVGTIAATD TnS000892793t06
PPRQLETVKSS-------ECLEALLSP-SIRSSAGTPREHH-----AFEPHPMIADAWEALRRSMVFFRSKPVGTIAALD Pp3c4_10940
PPRQLETVKSS-------ECLEALLSP-SIRSSAGTPREYH-----AFEPHPMIADAWETLRKSMVFFRSKPVGTIAALD Pp3c12_12810
PPRQLETAKSS-------ECLEALLSP-SIRSSAGTPREYH-----AFEPHPMIADAWERLRLSMVFYRSRPVGTIAALD Pp3c3_25210
PPRQLETVKSS-------ECLEALLSP-SIRSSAGTPREYH-----AFEPHPMIADAWERLRLSMVYFRDRPVGTIAALD Pp3c10_23170
PPSRHEIG-------------ENFSMN-WFRSASNTPRS-------LLDPNPAMIDAWEQLRRSLVYFRDKPIGTIAAND Sm89558
----------------------------------------------------MVQEAEDLLHKSLIYFRGKPIGTIAAIQ kfl00164_0180_v1.1
SSDRLDTMAAVNMAMVRTPSMLSLRSG-AAGGSHGTPGSSASGFDTPFAPHPMVADAWDRLRRSLVFFRGKPLGTIAALD TnS000602483t02
PVRGLDTVKST----------DCLES--MVQTGPSTPSSTR----ESIERHPIISEAWDALRRSLVYFRGKPVGTIAALD TnS000105403t01
```

β (cytosolic)

N-terminal sequences

```
NIQHTVHYLIG----------DLYQGRAIRIASASTPSSTR----GFIEPHPIIADAWDALRRSLVYFRGKPVGTIAALD TnS000982973t03
----------------------MIAG----PASAATPRSPL----HPYEPNPMIAEAWDSLRKSLVYFRGKPVGTIAALD Sm111393
VKS-IASLVDPEE--------AAPRSPSSDIFMSQL--PYE--------PNLTFSEAWICLRESIVKFAGEPVGTIAALD Azfi_s0064.g035495
VHDGLVTMSPDIV--------STAKSTGPPNDAEDE--EFE--------RNATFLDAWDCLRRSIVYFRGDPVGTIAAMD Azfi_s0040.g026676
-----------------------------------------------------MIGEAWEHLRRSIVYFRGNPVGTIAAND Sm151264
SSECLEALLTSP----------SMR-SGIDTPRSDR-IGFE--------PHPLYHEAWDALRKSLVYYRSKPVGTIAASD Azfi_s0393.g067806
SSERLDGMLFSSS--------ASFRSSGADTPRSEASHLCD--------PNPIFHEAWELLQRSIVLFKGEPVGTIAAND Azfi_s0003.g008094
GNHFSDILDIQHN--------GLMPSPSFNRSIASTPRSGATF------LHPMLFDAWEALKRSLVYFRGMPVGTIAALD Azfi_s0059.g034661
N---YDLINMDG-----------MLSPNITRSITNTPRS-ATFHSSHLELHPMLFDAWDALRRSLVYFRGMPVGTIAALD Azfi_s0001.g000547
G------IAISQ-----------FSHLNIHSTSTLTPRRLATILRS--PRCPIFIEAWDSLRRSLVYFRGAPVGTIAALD Azfi_s0096.g043780
-------------------------------TPHPRPTSFD--------QNLMFIEAWEHLRRSVVYYKGQAVGTMAALD GSVIVT01037429001
SDNVKAT-IRPCPS-------LSANLNNYDSVVEDASQRAG--------GSPVMEEAWERLNKSYIFYKGKPVGTLAAMD Eucgr.D02386.1
SDNAKSTALKQCPS-------VGVKLDVLDLNISQRLNGGGLLMS--PESCTMVDEAWERLNKSYVVFKGKPVGTLAAMD Potri.002G173600
NEEKVVCTLKQPLN-------VETNIGNLDQNTSPGSRLSGHQVTG-TEGASLVDEAWDRLQKSFVYFRGKPVGTLAAID GSVIVT01027176001
-HQPIEISPGG-------------SQCSLHEETPTDTNASH--------RHAIADAAWEALKRSMVYFRGQPIGTVAAID GRMZM2G477236
-LQTIEKSPGGG------------SQCSLHEETPTDTNASH--------RHAIADAAWEALKRSIVYFRGQPIGTVAAID Sb04g002180
---------TPR------------SSCSPSVESTIETHALH--------RHTIADAAWEALKQSIVYFRGQPIGTVAAID Bradi3g02560
-HQTLEPIKSPI------------SGCSPSVESTTDTNTVH--------RHTVADAAWEALKKSIVHFRGQPIGTVAAID LOC_Os02g03320
EMPLPVNGSVSSNG--------------------NAQSVGT---------KSIEDEAWDLLRQSVVFYCGSPIGTIAAND At5G22510
QKLKNGNEDLASSGM----------CGQPEATREKVQKISS---------KSMEDEAWELMRDSIAYYCNNPIGTIAAND Eucgr.J00457.1
QQLMREKEVLTSNGSANKEEESLATNGAVGTGRDASRKVSV---------DPTEEEAWELLRDSVVHYCGSPIGTIAAND Potri.008G024100
QQLTREKEGFASKAALNQEKESLATNGAVGTGRDASPKVSV---------DPIEEEAWELLRNSMVYYCGSPIGTIAAND Potri.010G236100
QELKPEMEGSIS-------------NGAVETARDTFVKVRV---------DSIEDEAWDLLRESMVYYCGSPIGTIAAKD GSVIVT01034753001
KQLKEEKTDSMS-------------NGAP-IASSTIHRANV---------DPIEEEAWDLLRDSVVYYCGSPIGTIAAND Eucgr.G02704.1
EPMRHEKGGFASNG----------KFAAGGTINDTLGKASI---------DSIEDEAWNLLRESIVFYCGYPIGTIAAND GSVIVT01031374001
TNIFERGSFALK------------------------GNEET---------QSIEEEAWDLLRASVVCYCGNPIGTIAAND Potri.004G186500
RELKVGEENFQSNG----------SLPPNGLVEDTLNRIAG---------NSIEDEAWELLRESMVYYCGSPVGTIAAKD Solyc11g007270
LGQAVSGNGGLN--------------GSAAKTPPQR--RKS---------SSVEDEAWELLQESMVYYCGSPVGTIAAND GRMZM2G040843
PGQAVGGNGSLN--------------GSVAKPPPQR--RKS---------SSVEDEAWELLQESMVYYCGSPVGTIAAND Sb04g021550
PGQVLGGNINLN--------------GNATKPLPQR--QKV---------SSVEDEAWDLLRDSIVNYCGIPVGTIAAND Bradi3g44770
PGQAVGGNGSVN--------------GSAAKPPPQR--RKA---------SSVEDEAWELLRESVVYYCGSPVGTIAAND LOC_Os02g32730
NGGAAKPSLAP-------------------TPQKR-RRAP---------CDVEEEAWGLLRESVVRYCGSPVGTIAACD Bradi5g09200
NGAAPAPAPAPA--------------EAPAKAPQRRQRRGP---------HDVEDEAWGLLRESVVRYCGSPVGTIAACD LOC_Os04g33490
MKQENGARPFSEG-------------FKTAASVNSRPRTNT---------ESIEDEAWHFLRAAMVYYCGSPVGTIAAND Solyc11g067050
AEQQHHHHHTATR-----------------EREMEMEKE---------SAMEKEAWELLQAAVVNYCGSPIGTIAAND TnS000875687t08
YVRHGGAVAPLTVGE----------IRKAALADKQSFSKRE---------SPLEHEAWELLRSAMVYYCGTPVGTIAAND Sm267827
YERKHG-----------------------------SFSK--------------------------------------- Azfi_s0345.g065806
LLNGIGQKGSSAPS--------------------SFNAPVE---------NALEKEAWDLLREAVVTYCGEPVGTIAAKD Pp3c6_27130
LLHG-GQNGSSD------------------------GAVAE---------SNLEREAWDLLRDAVVTYCGEPVGTIAAND Pp3c16_15060
LVEELSQRPESVDSN---------TNDGAGSNGNGYKALDE---------SPLEAEAWRLLKKAVVSYCGQPVGTIAAND Pp3c17_13250
LPLNLDVENFKGNS-----------------SDLTFKEARKE---------SPFEQEAWRLLRASIVHYQGCPVGTIAAND Sm443960
VLKAEEIARLTG-------------------------KTK---------KGIKKGLKKLVERSASSHGHHNAAKLVTTD kfl00563_0120_v1.1
```

α1 (plastidic)

N-terminal sequences

```
NTGE---QLNYDQVFVRDFVPSALAFLMNG--EPEIVKNFILKTLRLQS-WEKKIDRFHLGEGVMPA------SFKVLHD Potri.004G167500
NSEE---QVNYDQVFVRDFVPSALAFLMNG--EPEIVKNFILKTLRLQS-WEKKIDRFQLGEGVMPA------SFKVLHD Potri.009G129000
SSEE---NLNYDQVFVRDFFPSALAFLMNG--ESEIVKNFILKTLRLQS-WEKKIDRFQLGEGVMPA------SFKVLHD Eucgr.G01751.1
NSEE---KLNYDQVFVRDFVPSALAFLMNG--EPDIVKNFLLKTLRLQS-WEKKIDRFQLGEGVMPA------SFKVFHD At4G34860
NSDE---ELNYDQVFVRDFVPSALAFLMNG--EPEIVRNFLVKTLRLQS-WEKKVDRFQLGEGVMPA------SFKVLHD GSVIVT01024105001
NSDE---KLNYDQVFVRDFVPSALAFLMNR--EPEIVKNFLLKTLRLQS-WEKKIDRFQLGEGVMPA------SFKVLHD Solyc01g111100
HSVE---ELNYDQVFVRDFVPSALAFLMNG--QTEIVKNFLLRTLHLQS-REKMVDQFKLGEGVMPT------SFKVIHD Eucgr.F01661.1
HSVE---ELNYDQVFVRDFVPSALAFLMNG--EHEVVRNFLLKTLHLQS-REKMVDQFKLGAGVMPA------SFKVLHH Potri.005G239400
HSSD---ELNYDQVFVRDFVPSALAFLMNG--EPEIVKNFILKTLRLQS-WEKKVDQFKLGEGVMPA------SFKVFHD GSVIVT01009904001
NSAE---ELNYDQVFVRDFVPSALAFLMNG--EPDIVKNFLLKTLRLQS-REKKIDQFKLGDGVMPA------SFKVSHD Solyc04g081440
NTTD--EVLNYDQVFVRDFVPSALAFLMNG--EPDIVKHFLLKTLQLQG-WEKRVDRFKLGEGVMPA------SFKVLHD At1G35580
HASE--EVLNYDQVFVRDFVPSALAFLMNG--EPDIVKNFLLKTLQLQG-WEKRVDRFKLGEGVMPA------SFKVLHD At4G09510
HASE--EVLNYDQVFVRDFVPSALAFLMNG--EPDIVKNFLLKTLHLQG-WEKRIDRFKLGEGAMPA------SFKVLHD Eucgr.F02588.1
HASE--EVLNYDQVFVRDFVPSALAFLMNG--EPEIVKNFLLKTLHLQG-WEKRIDRFKLGEGAMPA------SFKVLHD GSVIVT01034488001
HASE--EVLNYDQVFVRDFVPSALAFLMNG--EPDIVKHFLLKTLYLQG-WEKRIDRFKLGEGAMPA------SFKVLHD Potri.013G110800
HASE--EVLNYDQVFVRDFVPSALAFLMNG--EPEIVKQFLLKTLHLQG-WEKRIDRFKLGEGAMPA------SFKVLHD Potri.019G082000
HAAE--EVLNYDQVFVRDFVPSALAFLMNG--EPDIVKNFLLKTLQLQG-WEKRVDRFKLGEGVMPA------SFKVLHD Solyc11g020610
HASE--EVLNYDQVFVRDFVPSALAFLMNG--EPDIVKNFLLKTLQLQG-WEKRVDRFKLGEGVMPA------SFKVLHD Solyc06g065210
HATE--EVLNYDQVFVRDFVPSALAFLMNG--EPDIVKNFLLKTIQIQG-REKRIDRFKLGEGAMPA------SFKVIHD At1G22650
HASE--EVLNYDQVFVRDFVPSALAFLMNG--EPDIVKNFLLKTLLLQG-WEKKVDRFKLGEGAMPA------SFKVMHD GRMZM2G118737
HASE--EVLNYDQVFVRDFVPSALAFLMNG--EPDIVKNFLLKTLLLQG-WEKKVDRFKLGEGAMPA------SFKVMHD Sb04g022350
HASE--EVLNYDQVFVRDFVPSALAFLMNG--EPDIVKNFLLKTLLLQG-WEKKVDRFKLGEGAMPA------SFKVMHD GRMZM2G170842
HASE--EVLNYDQVFVRDFVPSAMAFLMNG--EPEIVKNFLLKTVLLQG-WEKKVDRFKLGEGAMPA------SFKVLHD Bradi3g45530
HASE--EVLNYDQVFVRDFVPSALAFLMNG--EPEIVRHFLLKTLLLQG-WEKKVDRFKLGEGAMPA------SFKVLHD LOC_Os02g34560
HSSG--EVLNYDQVFVRDFVPSALAFLMNG--EPEIVRNFLLKTLLLQG-WEKRIDRFKLGEGAMPA------SFKVLKD GRMZM2G136139
HSAAA-EVLNYDQVFVRDFVPSALAFLMNG--EPEIVRNFLLKTLLLQG-WEKRIDRFKLGEGAMPA------SFKVLKD Sb04g029440
HSSD--EVLNYDQVFVRDFVPSALAFLMNG--EPEIVKNFLLKTLLLQG-WEKRIDRFKLGEGAMPA------SFKVLKD LOC_Os04g35280
HSAG--EVLNYDQVFVRDFVPSALAFLMNG--EPEIVKNFLLKTLLLQG-WEKRIDRFKLGEGAMPA------SFKVLKD Bradi5g10357
HASE--EVLNYDQVFVRDFVPSALAFLMNG--EPEIVKNFLLKTLHIQG-QDKMIDKFKLGDGAMPA------SFKVLHN At1G72000
HASE--EVLNYDQVFVRDFVPSALAFLMNN--ETDIVKNFLLKTLHLQS-SEKMVDRFKLGAGAMPA------SFKVDRN GRMZM2G007277
HASE--EVLNYDQVFVRDFVPSALAFLMNN--ETDIVKNFLLKTLHLQS-SEKMVDRFKLGAGAMPA------SFKVDRN Sb05g004770
HASE--EVLNYDQVFVRDFVPSALAFLMNN--ETDIVKNFLLKTLHLQS-SEKMVDRFKLGAGAMPA------SFKVDRN GRMZM2G115451
HASE--EVLNYDQVFVRDFFPSALAFLMNN--ETDIVKNFLLKTLHLQS-SEKMVDRFKLGAGAMPA------SFKVDRN LOC_Os11g07440
HASE--EVLNYDQVFVRDFVPSALAFLMNN--EPEIVKNFLLKTLHLQS-SEKMVDRFKLGAGAMPA------SFKVDRN Bradi4g24470
HSEE---ALNYNQVFVRDFIPSALAFLING--EYEIVKSFLLKTLQLQG-WEKQIDRFKLGEGVMPA------SFKVLHD TnS000892793t06
P-TE--DSLNYNQVFVRDFVPSALAFLMNG--EPEIVKNFLLKTLRLQS-IEKRIDCFTLGEGVMPA------SFKVLHD Pp3c4_10940
P-TE--DSLNYNQVFVRDFVPSALAFLMNG--EPEIVKNFLLKTLRLQS-IEKRIDCFTLGEGVMPA------SFKVLHD Pp3c12_12810
P-EE--DSLNYNQVFVRDFVPSALAFLMNG--EPEIVKNFLLRTLQLQS-VEKRIDCFTLGEGVMPA------SFKVLHD Pp3c3_25210
P-TE--DSLNYNQVFVRDFVPSALAFLMNG--EPEIVKNFLLKTLRLQS-IEKRIDCFTLGEGVMPA------SFKVLHD Pp3c10_23170
P-VE--ESLNYNQVFVRDFVPSALAFIMNG--EPEIAKNFLMKTLRLQA-WEKRIDCFTLGEGVMPA------SFKVLHD Sm89558
DGTE--EALNYNQVFVRDFVPSALAFLMKG--EPEIVRNFLMKTLRLQA-WEKRIDCFTLGEGVMPA------SFKVLHD kfl00164_0180_v1.1
S-SE--EPLNYNQVFVRDFVPSALAFLMNG--EPDIVKNFLLKTLHLQG-WEKRIDCFTLGEGVMPA------SFKVLHD TnS000602483t02
PSED---ALNYNQVFVRDFFPSALAFLMNG--EPEIVKNFLLKTLRLQA-WEKRIDCFTLGEGVMPA------SFKVLRD TnS000105403t01
PSDD--DALNYNQVFVRDFFPSALAFLMNG--EPYIVRNFLLKTLRLQA-WEKRIDCFTLGEGVMPA------SFKVLRD TnS000982973t03
PNEE---ALNYNQVFVRDFVPSALAFLMNG--EAEVVKNFLLKALRLQA-WEKRVDCFTLGEGVMPA------SFKVMQD Sm111393
PSEE---TLNYNQVFVRDFVPSALAFLMNN--ETEIVKNFLLKAIRLQSNCQKTIDNFTLGKGAMPA------SFKVHID Azfi_s0064.g035495
```

```
PSEN---ALNYDQVFVRDFVPSGLAFLMNN--EAGIVKSFLLRAIRLQSNSEKRIDNFTLGKGAMPA------SFKVEHN Azfi_s0040.g026676
SAEE---VLNYNQVFVRDFVPSALAFLMNG--ESDIVKNFLLKALRLQA-WEKRIDNFTLGQGAMPA------SFKVLHD Sm151264
HSEE---ALNYDQVFVRDFVPSALAFLMNG--EPEIVKNFLLKALRLQSNWEKRIDNFTLGKGAMPA------SFKVLHD Azfi_s0393.g067806
HTEE---ALNYDQVFVRDFVPSALAFLMNN--EAGIVKNFLLKAVRLQSNSEKRIDNFTLGKGAMPA------SFKVLHD Azfi_s0003.g008094
PTED---SLNYNQVFVRDFFPSALAFLMNG--EPEIVKNFLIKTLRLQA-LTKRIDNFTLGDGAMPA------SFKVLHD Azfi_s0059.g034661
PTED---SLNYNQVFVRDFFPSALAFLMNG--ESEIVKNFLIKTLRLQA-LTKRIDNFTLGAGVMPA------SFKVLHD Azfi_s0001.g000547
LTKD---TLNYNQVFVRDFFPSGLAFLMNG--EPEIVKNFLIKTLRLQA-LTKRIDNFTLGAGMMPV------SFKVLRD Azfi_s0096.g043780
NASG---ALNYDQVFVRDFVPSALAHLMKG--ELEIVKNFLLRTLHLQL-SVKGIDRFALGQGLMSASFKFLHSFKVLHN GSVIVT01037429001
PNAD---MLNYNQVFVRDFVPSGLACLMKKEMEPEIVKNFLLKTLHLQG-WEKRIDNFTLGEGVMPA------SFKVGFD Eucgr.D02386.1
TSAD---ALNYNQVFVRDFVPTGLACLMKEPPEPEIVRNFLLKTLHLQG-LEKRVDNFTLGEGVLPA------SFKVLYD Potri.002G173600
PSAE---PLNYNQVFVRDFVPSGLACLMKNPPEPEIVKNFLLQTLHLQG-CQKKIDNYTLGEGVMPA------SFKVLHD GSVIVT01027176001
KSQG--AALNYDQVFMRDFIPSALAFLMKG--EHLIVKNFLVETARLQS-REKMVDLFKLGQGVMPA------SFKVHHR GRMZM2G477236
KSQG--AALNYDQVFMRDFIPSALAFLMKG--EHLIVKNFLVETARLQS-REKMVDLFKLGQGVMPA------SFKVHHR Sb04g002180
RSQ---AELNYDQVFMRDFVPSALAFLMKG--EPLIVKNFLIETARLQS-REKMVDLFKLGQGVMPA------SFKVHHS Bradi3g02560
KSQ---GALNYDQVFMRDFVPSALAFLMKG--EPTIVKNFLLETARLQL-REKMVDLFKLGQGVMPA------SFKVHHC LOC_Os02g03320
PNST--SVLNYDQVFIRDFIPSGIAFLLKG--EYDIVRNFILYTLQLQS-WEKTMDCHSPGQGLMPC------SFKVKTV At5G22510
PSCP--SILNYDQVFIRDFVPSGIAFLLKG--EYDIVRNFILHTLQLQS-WEKTMDSYSPGQGLMPA------SFKVRTV Eucgr.J00457.1
PTSS--SVLNYDQVFIRDFIPSGIAFLLKG--EYDIVRNFLLHTLQLQS-WEKTMDCHSPGQGLMPA------SFKVRTF Potri.008G024100
PTSS--SVLNYDQVFIRDFIPSGIAFLLKG--EYDIVRNFLLHTLQLQS-WEKTMDCHSPGQGLMPA------SFKVRTV Potri.010G236100
PTSS--NVLNYDQVFIRDFIPSGIAFLLKG--EYDIVRNFILHTLQLQS-WEKTMDCHSPGQGLMPA------SFKVRTV GSVIVT01034753001
PTSS--NVLNYDQVFIRDFIPSGIAFLLKG--EYDIVRNFILHTLQLQS-WEKTMDCHSPGQGLMPA------SFKVRTV Eucgr.G02704.1
PSNS--SSLNYDQVFIRDFIPSGIAFLLKG--EYDIVRSFILHTLQLQS-WEKTMDCHSPGQGLMPA------SFKVRTV GSVIVT01031374001
PNST--SILNYDQVFIRDFIPSGIAFLLKG--EYDIVRNFILYTLQLQS-WEKTMDCYSPGQGLMPA------SFKVRTV Potri.004G186500
PTSSTADVLNYDQVFIRDFIPSGIAFLLKG--EYEIVRNFILHTLQLQS-WEKTMDCHSPGQGLMPA------SFKVRTV Solyc11g007270
PNDS--DPVNYDQVFIRDFIPSGIAFLLKG--EYEIVRNFILHTLQLQS-WEKTMDCHSPGQGLMPA------SFKVRTI GRMZM2G040843
PNDS--DPVNYDQVFIRDFIPSGIAFLLKG--EYEIVRNFILHTLQLQS-WEKTMDCHSPGQGLMPA------SFKVRTI Sb04g021550
PNDS--NPANYDQVFIRDFIPSGVAFLLKG--EYEIVRNFILHTLQLQS-WEKTMDCHSPGQGLMPA------SFKVRTI Bradi3g44770
PNDA--NPMNYDQVFIRDFIPSGIAFLLKG--EYEIVRNFILHTLQLQS-WEKTMDCHSPGQGLMPA------SFKVRTI LOC_Os02g32730
PNDA--CPLNYDQVFIRDFVPSGIAFLLKG--EYDIVRNFILHTLQLQS-WEKTMDCHSPGQGLMPA------SFKVRVI Bradi5g09200
PNDA--SPLNYDQVFIRDFVPSGIAFLLKG--DYEIVRNFILHTLQLQS-WEKTMDCHSPGQGLMPA------SFKVRVV LOC_Os04g33490
PSEA--TMLNYDQVFIRDFIPSGIAFLLKG--EYDIVRNFILHTLQLQS-WEKTMDCYSPGQGLMPA------SFKVRTI Solyc11g067050
PADA--SILNYDQVFIRDFIPSAIAFMLKG--EHDIVRNFLLHTLQLQS-WEKTMDCHSPGQGLMPA------SFKVRTV TnS000875687t08
PTDG--HPLNYDQVFIRDFIPSAIAFLLKG--ETDIVRNFLLHTLQLQS-WEKTVDCYNPGQGLMPA------SFKVRTV Sm267827
-------------------VLQSIAFLLKG--EFEIVRHFILYTLQLQS-WEKTVDCYSPGQGLMPA------SFKVGLI Azfi_s0345.g065806
PTDP--NPLNYDQVFIRDFIPSAVAFLLKG--ETEIVRNFLLHTLQLQS-WEKTVDCYSPGQGLMPA------SFKVRTV Pp3c6_27130
PTDP--HPLNYDQVFIRDFIPSAIAFLLKG--ETEIVRNFLLHTLQLQS-WEKTVDCYCPGQGLMPA------SFKVRTV Pp3c16_15060
PTDP--YPLNYDQVFIRDFIPSAIAFLLKG--EHEIVRNFIHHTLQLQS-WEKTVDCYTPGQGLMPA------SFKVQTV Pp3c17_13250
PTDA--SALNYDQVFIRDFVPAGIAFLLKG--EPAIVRNFLLCTLRLQS-WEKTVDFYSPGQGLMPA------SFKVQSV Sm443960
WTDK-----------AATRSSARKQLMIEE--AWRLLRGSVVN----------------PGEGLMPA------SFKVQVR kfl00563_0120_v1.1
```

```
PVRN------SETLMADFGESAIGRVAPVDSGFWWIFLLRAYTKSTGDTSLAEMPECQKGMRLILSLCLSEGFDTFPTLL Potri.004G167500
PVTH------NETLMADFGESAIGRVAPVDSGFWWIFLLRAYTKSTGDTSLAEKPECQKGMRLILSLCLSEGFDTFPTLL Potri.009G129000
PVRN------NDTLIADFGESAIGRVAPVDSGFWWIILLRAYTKSTGDTSLAELSECQKGMRLILSLCLSEGFDTFPTLL Eucgr.G01751.1
PVRN------HETLIADFGESAIGRVAPVDSGFWWIILLRAYTKSTGDSSLADMPECQKGIRLILSLCLSEGFDTFPTLL At4G34860
PVRN------SDTLIADFGESAIGRVAPVDSGFWWIILLRAYTKSTGDSTLAELPECQKGMRLILTLCLSEGFDTFPTLL GSVIVT01024105001
PVRN------TETLIADFGESAIGRVAPIDSGFWWIILLRAYTKSTGDTSLSELPECQKGMRLILSLCLSEGFDTFPTLL Solyc01g111100
PARN------HETLTADFGESAIGRVAPVDSGFWWIILLRAYTKSTGDSSLAELPECQRGMRLILSLCLSEGFDTFPTLL Eucgr.F01661.1
PDRN------IETLMADFGESAIGRVAPVDSGFWWIILLRAYTKSTGDSSLAEMPECQRGMRLILNLCLSEGFDTFPTLL Potri.005G239400
PVRN------YETLIADFGESAIGRVAPVDSGFWWIILLRAYTKSTGDSSLAEMPECQRGMRLILSLCLSEGFDTYPTLL GSVIVT01009904001
PVRN------YETITADFGESAIGRVAPVDSGFWWIILLRAYTKSTGDTSLAEMPECQRGIRLILELCLSEGFDTFPTLL Solyc04g081440
PIRE------TDNIVADFGESAIGRVAPVDSGFWWIILLRAYTKSTGDLTLSETPECQKGMKLILSLCLAEGFDTFPTLL At1G35580
PVRK------TDTIIADFGESAIGRVAPVDSGFWWIILLRAYTKSTGDLTLSETPECQRGMRLILSLCLSEGFDTFPTLL At4G09510
PIRK------TDTLMADFGESAIGRVAPVDSGFWWIILLRAYTKSTGDLSLAETEECQKGMRLICSLCLSEGFDTFPTLL Eucgr.F02588.1
PIRK------TDTLIADFGESAIGRVAPVDSGFWWIILLRAYTKSTGDLSLAETPECQKGMKLILTLCLSEGFDTFPTLL GSVIVT01034488001
PIRK------TDSLVADFGESAIGRVAPVDSGFWWIILLRAYTKSTGDLSLAERPECQKGMKLILTLCLSEGFDTFPTLL Potri.013G110800
PIRK------TDSLVADFGESAIGRVAPVDSGFWWIILLRAYTKSTGDLSLAETPECQKGMRLILTLCLSEGFDTFPTLL Potri.019G082000
PVRK------TDTIMADFGESAIGRVAPVDSGFWWIILLRAYTKSTGDVSLAETPECQRGMRLILSLCLSEGFDTFPTLL Solyc11g020610
PVRK------TDTIVADFGESAIGRVAPVDSGFWWIILLRAYTKSTGDLSLSETTECQKGMRLILSLCLSEGFDTFPTLL Solyc06g065210
PIKE------TDSINADFGESAIGRVAPVDSGFWWIILLRAYTKSTGDTSLAETSECQKGMRLILSLCLSEGFDTFPTLL At1G22650
AKKG------VETLHADFGESAIGRVAPVDSGFWWIILLRAYTKTTGDLTLAETPECQKGMRLILSLCLSEGFDTFPTLL GRMZM2G118737
AKKG------VETLHADFGESAIGRVAPVDSGFWWIILLRAYTKTTGDMTLAETPECQKGMRLILSLCLSEGFDTFPTLL Sb04g022350
AKKG------VETLHADFGESAIGRVAPVDSGFWWIILLRAYTKSTGDLTLAETPECQKGMRLILSLCLSEGFDTFPTLL GRMZM2G170842
DKKG------TDTLHADFGESAIGRVAPVDSGFWWIILLRAYTKSTGDLTLAEKPECQKAMRLILSLCLSEGFDTFPTLL Bradi3g45530
SKKG------VDTLHADFGESAIGRVAPVDSGFWWIILLRAYTKSTGDLTLAETPECQKGMRLILSLCLSEGFDTFPTLL LOC_Os02g34560
PKRG------VDKLVADFGESAIGRVAPVDSGFWWIIILRAYTKSTGDMTLAETPMCQKGIRLIMNQCLAEGFDTFPTLL GRMZM2G136139
PKRG------VDKLVADFGESAIGRVAPVDSGFWWIIILRAYTKSTGDMTLAETPMCQKGIRLIMNQCLAEGFDTFPTLL Sb04g029440
AKRGG-----AERLVADFGESAIGRVAPVDSGFWWIILLRAYTKSTGDLSLAETAECQRGIRLIMNQCLAEGFDTFPTLL LOC_Os04g35280
PKRG------VDTLSADFGESAIGRVAPADSGFWWIILLRAYTKSTGDLTLAETPECQKGIRLIMNQCLAEGFDTFPTLL Bradi5g10357
PIKK------TDTIIADFGESAIGRVAPVDSGFWWIILLRAYTKSTGDHSLAERPECQKGMRLILSLCLSEGFDTFPTLL At1G72000
KNRN------TETLVADFGESAIGRVAPVDSGFWWIILLRAYTKYTGDVSLSESPECQKCMRLILNLCLSEGFDTFPTLL GRMZM2G007277
KNRN------TETLVADFGESAIGRVAPVDSGFWWIILLRAYTKYTGDVSLSESPDCQKCMRLILNLCLSEGFDTFPTLL Sb05g004770
KNRN------TETLVADFGESAIGRVAPVDSGFWWIILLRAYTKYTGDVSLSESPECQKCMRLILNLCLSEGFDTFPTLL GRMZM2G115451
RNRN------TETLVADFGESAIGRVAPVDSGFWWIILLRAYTKYTADTSLAESPECQNCMRLILNLCLSEGFDTFPTLL LOC_Os11g07440
KSRN------TETLVADFGESAIGRVAPVDSGFWWIILLRAYTKYTGDVSLSESPDCQKCMRLILNLCLSEGFDTFPTLL Bradi4g24470
PVRN------TDTMIADFGESAIGRVAPVDSGFWWIILLRAYTRSTGDHTLAEMSDCQRGMRLILALCLAEGFDTFPTLL TnS000892793t06
PVRK------TDTMIADFGESAIGRVAPVDSGFWWIILLRAYTKSTGDHTLADMPDCQRGMRLILTLCLADGFDTFPTLL Pp3c4_10940
PARK------TDTMIADFGESAIGRVAPVDSGFWWIILLRAYTKSTGDHSLADMPDCQRGMRLILTLCLADGFDTFPTLL Pp3c12_12810
PVRK------TDTMIADFGESAIGRVAPVDSGFWWIILLRAYTKSTGDYTLADMPDCQRGMRLILYLCLADGFDTFPTLL Pp3c3_25210
PVRK------TDTMIADFGESAIGRVAPVDSGFWWIILLRAYTKSTGDHTLADMADCQRGMRLILTLCLADGFDTFPTLL Pp3c10_23170
PSR-------TDTMIADFGESAIGRVAPVDSGFWWIILLRAYVKATGDHNLSDDPDCQRGIRLILTLCLSEGFDTFPTLL Sm89558
PVRN------TDTMIADFGESAIGRVAPVDSGFWWIILLRAYGRATGDHTLADHPDIQRGIILILQLCLADGFDTFPTLL kfl00164_0180_v1.1
PERN------SDTLIADFGESAIGRVAPVDSGFWWIILLRAYTKHTGDHTLVDMPDIQRGIRLIMSLCLSEGFDTFPTLL TnS000602483t02
PTRD------TDTMIADFGESAIGRVAPVDSGFWWIILLRAYTKSTGDHSLADMADCQRGIRLILTLCLAEGFDTFPTLL TnS000105403t01
PTRD------TDTMIADFGESAIGRVAPVDSGFWWIILLRAYTKSTGDFSLADMADCQRGIRLILTLCLAEGFDTFPTLL TnS000982973t03
PVRG------TETMLADFGEAAIGRVAPVDSGFWWIILLRAYTKSTGDFTLAEMPDCQRGMRLILSLCLAEGFDTFPTLL Sm111393
PKRN------QEKITADFGESAIGRVAPVDSGFWWIILLRAYCKCVGDSSLAERPDCQQGMGLILNLCLAEGFDTFPTLL Azfi_s0064.g035495
```

```
PIRA------QDTIVADFGESAIGRVAPVDSGFWWIFLLRAYTKATGDWSLAESPECQRGMELILNLCLSEGFDTFPTLL Azfi_s0040.g026676
PVRR------TDTMVADFGESAIGRVAPVDSGFWWIILLRAYTRSTGDHSLADMPDCQRGIKLILTLCLAEGFDTFPTLL Sm151264
PVKN------NDSIMADFGESAIGRVAPVDSGFWWIILLRAYTKSTGDQSLAETADCQRGIRLVLSLCLSEGFDTFPTLL Azfi_s0393.g067806
TERK------QERLMADFGESAIGRVAPVDSGFWWIILLRAYTKSTADHTLADMPDCQRGIRLVLNLCLSEGFDTFPTLL Azfi_s0003.g008094
VNRK------TDTMVADFGESAIGRVAPVDSGFWWIILLRAYTKSTGNHALADMPDFQRGMRLILSLCLSEGFDTFPTLL Azfi_s0059.g034661
ANRN------TDTMIADFGESAIGRVAPVDSGFWWIILLRAYTKSTGDHTLADMPDFQRGMRLILSLCLSEGFDTFPTLL Azfi_s0001.g000547
KDRN------TETMVGDFGGSAIGRVAPVDSGFWWIILLRAYTKSTGDNSLAQRPDFQHGIRLILSLCLAEGFNTFPTLL Azfi_s0096.g043780
PVRG------VDTLIADFGETAIGRVAGVDSGFWWIILLHAYTRATGDYSLSHRPECQNGMKLILSVCLAEGFDTFPTLL GSVIVT01037429001
LQDK------KERLVADFGGSAIGRVAPVDSGFWWIILLRSYTKCTHDYSLADLPVVQKGMKLILNLCLSDGFDTFPTLL Eucgr.D02386.1
SDLE------KETLLVDFGASAIGRVAPVDSGFWWIILLRSYIKRTRDYALLDRPEVQNGMKLILKLCLSDGFDTFPTLL Potri.002G173600
PKTQ------KETLVADFGGSAIGRVAPVDSVFWWIILLRSYTKCTGDNSFSELPQVQGGIKSILKLCLCDGFNNFPTLL GSVIVT01027176001
NPTQK-----TESLLADFGETAIGRVAPVDSGLWWIILLRAYTKWTGDNSLAESTNCQRAMHLILRLCLSEGCDTSPALL GRMZM2G477236
NPTQK-----TESLLADFGETAIGRVAPVDSGLWWIILLRAYTKWTGDNSLAESPNCQRAMHLILRLCLSEGCDTSPALL Sb04g002180
HPTKK-----TESLLADFGEIAIGRVAPVDSGLWWIFLLRAYTKWTRDNSLAESPHCQRAMRLILKLWLSEGFDTSPALL Bradi3g02560
NSKHK-----TESLLADFGETAIGRVAPVDSGLWWIILLHAYTIWTRDNSLAESPECQRAMRLILKLCLSEGFDTSPALL LOC_Os02g03320
PLDGDD-SMTEEVLDPDFGEAAIGRVAPVDSGLWWIILLRAYGKCTGDLSVQERVDVQTGIKMILKLCLADGFDMFPTLL At5G22510
PLNGDN-SATEEILDPDFGEAAIGRVAPVDSGLWWIILLRAYGKCSGDLSVQERVDVQTGIKMILKLCLADGFDMFPTLL Eucgr.J00457.1
PLDGDD-SATEEVLDPDFGEAAIGRVAPVDSGLWWIILLRAYGKCSGDLSVQERIDVQTGIKMILRLCLADGFDMFPTLL Potri.008G024100
RLDGDDDFATEEVLDPDFGEAAIGRVAPVDSGLWWIILLRAYGKCSGDLSLQERIDVQTGIKMILRLCLADGFDMFPTLL Potri.010G236100
PLDGDD-SATEEVLDPDFGEAAIGRVAPVDSGLWWIILLRAYGKCSGDLSVQERIDVQTGIKMILRLCLADGFDMFPTLL GSVIVT01034753001
PLDGDD-SATEEVLDPDFGEAAIGRVAPVDSGLWWIILLRAYGKISGDLSVQERIDVQTGIKMILKLCLADGFDMFPTLL Eucgr.G02704.1
PLDGDD-SATEDVLDPDFGEAAIGRVAPVDSGLWWIILLRAYGKCSGDLSVQERFDVQTGIKMILKLCLADGFDMFPTLL GSVIVT01031374001
PLDSED-SATEEVLDADFGEAAIGRVAPVDSGLWWIILLRAYGKCSGDLSVQERVDVQTGMKMILRLCLADGFDMFPTLL Potri.004G186500
PLDGDD-SATEEVLDPDFGEAAIGRVAPVDSGLWWIILLRAYGKSSGDLSVQERIDVQTGIKMILRLCLADGFDMFPTLL Solyc11g007270
PLDGDE-DATEEVLDPDFGEAAIGRVAPVDSGLWWIILLRAYGKCSGDLSVQERIDVQTGMKMILKLCLADGFDMFPTLL GRMZM2G040843
PLDGDE-DATEEVLDPDFGEAAIGRVAPVDSGLWWIILLRAYGKCSGDLSVQERIDVQTGMKMILKLCLADGFDMFPTLL Sb04g021550
PLDGDD-DATEEVLDPDFGEAAIGRVAPVDSGLWWIILLRAYGKCSGDLSVQERIDVQTGIKMILKLCLTDGFDMFPTLL Bradi3g44770
PLDGDE-DATEEVLDPDFGEAAIGRVAPVDSGLWWIILLRAYGKCSGDLTVQERIDVQTGIKMILKLCLADGFDMFPTLL LOC_Os02g32730
PLD-DN-GTTEEVLDPDFGEAAIGRVAPVDSGLWWIILLRAYGKCSGDMSFHERIDVQTGIKLILKLCLADGFDMFPTLL Bradi5g09200
PLDGDD-DVTEEVLDPDFGEAAIGRVAPVDSGLWWIILLRAYGKCSGDLSVQERIDVQTGIKMILKLCLADGFDMFPTLL LOC_Os04g33490
PLDNDE-SATEDVLDPDFGEAAIGRVAPVDSGLWWIILLRAYGKCSGDLSLQERVDVQTGMKMILRLCLADGFDMFPTLL Solyc11g067050
PLEADE-SATEDVLDPDFGESAIGRVAPVDSGLWWIILLRAYGKYTGDSSLQERTDVQTGMKMILKLCLADGFDMFPTLL TnS000875687t08
PLEGDPANGTEEVLDPDFGEAAIGRVAPVDSGLWWIILLRAYGKSTGDYTLQERVDVQTGMKMILKLCLADGFDMFPTLL Sm267827
PRDGD-ENVMEETLDPDFGEAAIGRVAPVDSGLWWIILLRAYGKCTGDLSLQERVDVQTGIKMILKLCLADGFDMFPTLL Azfi_s0345.g065806
HIDGNEENGTEEILDPDFGEAAIGRVAPVDSGLWWIILLRAYGKCTGDKSVQERVDVQTGIKMILKVCLADGFDMFPTLL Pp3c6_27130
YLDGDETKGTEEILDPDFGEAAIGRVAPVDSGLWWIILLRAYGKCTGDISLQERVDVQTGIKMILKVCLADGFDMFPTLL Pp3c16_15060
FLDREGVKETEEILQPDFGEAAIGRVAPVDSGLWWIILLRAYGKCTGDLTLQERVDVQTGIKMILKVCLADGFDMFPSLL Pp3c17_13250
AAEEDT---CEEILDPDFGEAAIGRVAPVDSGLWWIILLRAYGKSTGDLSLQERMDVQTGIRMILKLCLSDGFDMFPTLL Sm443960
MCSDGK---EHERLDADFGESAIGRVAPVDSGLWWIILLRAYLRVTKDTAVQTSPAVQKGIRLILKLMLRDGFDMFPTIL kfl00563_0120_v1.1
```

```
CADGCCMIDRRM--------------------------------------------GVYGYPIEIQALFFMALRCALLL Potri.004G167500
CADGCCMVDRRM--------------------------------------------GVYGYPIEIQALFFMALRCALLL Potri.009G129000
CADGCCMIDRRM--------------------------------------------GVYGYPIEIQALFFMALRCALLL Eucgr.G01751.1
CADGCCMIDRRM--------------------------------------------GVYGYPIEIQALFFMALRCALLL At4G34860
CADGCCMIDRRMDIVNTMMQINQPHDYLDQVIVKLK--------------------GVYGYPIEIQALFFMALRCALLL GSVIVT01024105001
CADGCSMIDRRM--------------------------------------------GVYGYPIEIQALFFMALRCALLL Solyc01g111100
CADGCCMIDRRM--------------------------------------------GVYGYPIEIQALFFMALRCALVL Eucgr.F01661.1
CADGCCMIDRRM--------------------------------------------GVYGYPIEIQALFFMALRCALIL Potri.005G239400
CADGCCMIDRRM--------------------------------------------GVYGYPIEIQALFFMALRCALLL GSVIVT01009904001
CADGCSMIDRRM--------------------------------------------GVYGYPIEIQALFFMALRCALFL Solyc04g081440
CADGCSMIDRRM--------------------------------------------GVYGYPIEIQALFFMALRSALSM At1G35580
CADGCSMVDRRM--------------------------------------------GVYGYPIEIQALFFMALRCALSM At4G09510
CADGCSMVDRRM--------------------------------------------GIYGYPIEIQALFFMALRCALAM Eucgr.F02588.1
CADGCSMVDRRMLWVFNPSFSFSAPFWLYPIVVFSSLAPGIAAQIGNTLRLLTQTQGIYGYPIEIQALFFMALRCALAM GSVIVT01034488001
CADGCSMIDRRM--------------------------------------------GIYGYPIEIQALFFMALRSASSM Potri.013G110800
CADGCSMIDRRM--------------------------------------------GIYGYPIEIQALFFMALRSACSL Potri.019G082000
CADGCSMIDRRM--------------------------------------------GIYGYPIEIQALFFMALRSALAM Solyc11g020610
CADGCSMIDRRM--------------------------------------------GVYGYPIEIQALFFMALRSALAM Solyc06g065210
CADGCSMIDRRM--------------------------------------------GVYGYPIEIQALFFMALRSAMSM At1G22650
CADGCCMIDRRM--------------------------------------------GVYGYPIEIQALFFMALRCALQM GRMZM2G118737
CADGCCMIDRRM--------------------------------------------GVYGYPIEIQALFFMALRCALQM Sb04g022350
CADGCCMIDRRM--------------------------------------------GVYGYPIEIQALFFMALRCALQM GRMZM2G170842
CADGCCMIDRRM--------------------------------------------GVYGYPIEIQSLFFMALRCALLM Bradi3g45530
CADGCCMIDRRM--------------------------------------------GVYGYPIEIQALFFMALRCALQL LOC_Os02g34560
CADGCCMIDRRM--------------------------------------------GVYGYPIEIQALFFMALRCALLM GRMZM2G136139
CADGCCMIDRRM--------------------------------------------GVYGYPIEIQALFFMALRCALVM Sb04g029440
CADGCCMIDRRM--------------------------------------------GVYGYPIEIQALFFMALRCALLM LOC_Os04g35280
CADGCCMIDRRM--------------------------------------------GVYGYPIEIQALFFMALRCALLL Bradi5g10357
CADGCSMVDRRM--------------------------------------------GIYGYPIEIQALFFMALRSALSM At1G72000
CTDGCSMIDRRM--------------------------------------------GIYGYPIEIQALFYMALRCALQM GRMZM2G007277
CTDGCSMIDRRM--------------------------------------------GIYGYPIEIQALFYMALRCALQM Sb05g004770
CTDGCSMIDRRM--------------------------------------------GIYGYPIEIQALFYMALRCALQM GRMZM2G115451
CTDGCSMIDRRM--------------------------------------------GIYGYPIEIQALFYMALRCALQM LOC_Os11g07440
CTDGCSMIDRRM--------------------------------------------GIYGYPIEIQALFYMALRCALQM Bradi4g24470
CADGCCMVDRRM--------------------------------------------GIYGYPIEIQALFFMALRCARQL TnS000892793t06
CADGCCMVDRRM--------------------------------------------GIYGYPIEIQSLFFMALRSAKSL Pp3c4_10940
CADGCCMVDRRM--------------------------------------------GIYGYPIEIQSLFLMALRSAKSL Pp3c12_12810
CADGCCMVDRRM--------------------------------------------GIYGYPIEIQSLFFMALRSAKSL Pp3c3_25210
CADGCCMVDRRM--------------------------------------------GIYGYPIEIQSLFFMALRCAKVL Pp3c10_23170
CADGCCMIDRRM--------------------------------------------GIYGYPIEIQALFFMALRCAKFL Sm89558
CADGCCMIDRRM--------------------------------------------GIYGYPIEIQALFYHALRCAKQM kfl00164_0180_v1.1
CADGCCMIDRRM--------------------------------------------GIYGYPIEIQALFFFALRCAKLL TnS000602483t02
CADGCCMVDRRM--------------------------------------------GIYGYPIEIQSLFFMALRCARKL TnS000105403t01
CADGCCMVDRRM--------------------------------------------GIYGHPIEIQALFFMALRCARKL TnS000982973t03
CADGCCMIDRRM--------------------------------------------GIYGYPIEIQSLFFMALRCAKTL Sm111393
CADGCSMVDRRMG--------------------------------------------IYGYPIEIQSLFFMALKCARSM Azfi_s0064.g035495
```

```
CADGCSMVDRRMVSKSSSLIACPRAW-------------------------------GIYGYPIEIQSLFYMALRCARIM Azfi_s0040.g026676
CADGCCMVDRRMG---------------------------------------------IYGYPIEIQALFFMALRCAKTM Sm151264
CADGCCMIDRRMG---------------------------------------------IYGYPIEIQALFYMALRCAKKL Azfi_s0393.g067806
CADGCCMIDRRMG---------------------------------------------IYGYPIEIQALFYMALRCAKIM Azfi_s0003.g008094
CADGCCMVDRRM---------------------------------------------GIYGYPVEIQTLFFLALRCARVL Azfi_s0059.g034661
CADGCCMVDRRM---------------------------------------------GIYGYPVEIQTLFFLALRCARVL Azfi_s0001.g000547
CADGCCMVDRRM---------------------------------------------GIYGYHVEIQTLFFFALRCARSL Azfi_s0096.g043780
CADGCGMADRRM---------------------------------------------GVYGYPIEIQALFFMALRCAVHL GSVIVT01037429001
CADGCSMIDRRM---------------------------------------------GVYGYPIEIQALFYFALRCAQQL Eucgr.D02386.1
CADGCSMIDRRM---------------------------------------------GIYGYPIEIQALFYFALRCAKQM Potri.002G173600
CADGCCMVDRSM---------------------------------------------GINGYPIEIQSLFYFALRCARQM GSVIVT01027176001
CADGCSMIDRRM---------------------------------------------GIYGYPIEIQALFFMAMRCALSL GRMZM2G477236
CADGCSMIDRRM---------------------------------------------GIYGYPIEIQALFFMAMRCALSL Sb04g002180
CADGCSMIDRRM---------------------------------------------GIYGYPIEIQALFFMALRCALSL Bradi3g02560
CADGCSMIDRRM---------------------------------------------GIYGYPIDIQALFFMALRCAVTL LOC_Os02g03320
VTDGSCMIDRRM---------------------------------------------GIHGHPLEIQALFYSALVCAREM At5G22510
VTDGSCMIDRRM---------------------------------------------GIHGHPLEIQSLFYSALLCAREM Eucgr.J00457.1
VTDGSCMIDRRM---------------------------------------------GIHGHPLEIQALFYSALLCAKEM Potri.008G024100
VTDGSCMIDRRM---------------------------------------------GIHGHPLEIQALFYSALLCAREM Potri.010G236100
VTDGSCMIDRRM---------------------------------------------GIHGHPLEIQALFYSALLCAREM GSVIVT01034753001
VTDGSCMIDRRM---------------------------------------------GIHGHPLEIQALFYSALMSAREM Eucgr.G02704.1
VTDGSCMIDRRM---------------------------------------------GIHGHPLEIQALFYSALLCAREM GSVIVT01031374001
VTDGSCMIDRRM---------------------------------------------GIHGHPLEIEALFYSALLCAREM Potri.004G186500
VTDGSCMIDRRM---------------------------------------------GIHGHPLEIQALFHSALLCAREM Solyc11g007270
VTDGSCMIDRRM---------------------------------------------GIHGHPLEIQALFYSALLCAREM GRMZM2G040843
VTDGSCMIDRRM---------------------------------------------GIHGHPLEIQALFYSALLCAREM Sb04g021550
VTDGSCMIDRRM---------------------------------------------GIHGHPLEIQALFYSALLSAREM Bradi3g44770
VTDGSCMIDRRM---------------------------------------------GIHGHPLEIQALFYSALLCAREM LOC_Os02g32730
VTDGSCMIDRRM---------------------------------------------GIHGHPLEIQALFYSALLSAREL Bradi5g09200
VTDGSCMIDRRM---------------------------------------------GIHGHPLEIQALFYSALLCAREM LOC_Os04g33490
VTDGSCMIDRRM---------------------------------------------GIHGHPLEIQALYYSALLGAREM Solyc11g067050
VTDGSCMIDRRM---------------------------------------------GIHGHPLEIQALFYSALRCAREM TnS000875687t08
VTDGSCMIDRRM---------------------------------------------GIHGHPLEIQALFYSALRCAREM Sm267827
VTDGSCMIDRRM---------------------------------------------GIHGHPLEIQALFYSALRCAREM Azfi_s0345.g065806
VTDGSCMIDRRM---------------------------------------------GIHGHPLEIQALFYHALRCAREI Pp3c6_27130
VTDGSCMIDRRM---------------------------------------------GIHGHPLEIQALFYQALRCAREI Pp3c16_15060
VTDGSMMIDRRM---------------------------------------------GTHGHPLEIQALFYHALRCAKEM Pp3c17_13250
VTDGSCMIDRRM---------------------------------------------GIHGHPLEIQALFYSALQCAKEM Sm443960
VPDGSCMIDRRM---------------------------------------------GVDGHPLEIQSLYYAALRCAREM kfl00563_0120_v1.1
```

L-KQDE--EGKEFVERITKRLHALSFHMRSYYWIDLKQLNDIYRYKTEEYSHTAVNKFNVIPDSLPEWIFDFMPVHGGYF Potri.004G167500
L-KQDE--EGNEFVERITKRLHALSFHMRSYYWIDLKQLNDIYRYKTEEYSHTAVNKFNVIPDSLPEWIFDFMPVRGGYF Potri.009G129000
L-KQDV--EGKEFVERIVKRLHALTYHMRGYFWIDLKHLNDIYRYKTEEYSHTAVNKFNVIPDSLPEWIFDFMPTRGGYF Eucgr.G01751.1
L-KHDG--EGKEMVEQIVKRLHALSYHMRSYFWLDLKQLNDIYRYKTEEYSHTAVNKFNVIPDSLPEWVFDFMPPHGGFF At4G34860
L-KQDD--QGKEFIERIVKRLHALSYHMRSYFWLDMKQLNDIYRYKTEEYSHTAVNKFNVIPDSIPEWIFDFMPTYGGYF GSVIVT01024105001
L-KHDA--EGKEFVERIVKRLHALSYHMRNYFWLDLKQLNDIYRYKTEEYSHTAVNKFNVMPDSLPEWVFDFMPVSGGYF Solyc01g111100
L-KQEG--EGKDLVERIVQRLHALSYHMRNYFWLDLKQLNDIYRYKTEEYSHTAVNKFNVMPDSLPDWVFDFMPSRGGYF Eucgr.F01661.1
L-KQDD--EGKEFVDRVATRLHALSYHMRNYFWLDMKQLNDIYRYKTEEYSHTAVNKFNVMPDSLPDWVFDFMPTRGGYF Potri.005G239400
L-KQDD--KGKEFVELISKRLHALSYHMQSYFWLDIKQLNDIYRYKTEEYSHTAVNKFNVMPDSLPDWVFDFMPSRGGYF GSVIVT01009904001
L-KHDE--ENQECCDAIIKRLHALSFHMRSYYWLDIKQLNDIYRYKTEEYSHTAVNKFNVMPDSLPEWVFDFMPTRGGYF Solyc04g081440
L-KPDG--DGREVIERIVKRLHALSFHMRNYFWLDHQNLNDIYRFKTEEYSHTAVNKFNVMPDSIPEWVFDFMPLRGGYF At1G35580
L-KPDE--EGRDFIERIVKRLHALSFHMRSYFWLDFQQLNDIYRYKTEEYSHTAVNKFNVMPDSIPDWVFDFMPLRGGYF At4G09510
L-KHDS--EGKECIERIVKRLHALSYHMRSYFWLDFQQLNDIYRYKTEEYSHTAVNKFNVIPDSIPDWVFDFMPTRGGYF Eucgr.F02588.1
L-KQDS--EGKECIERIVKRLHALSYHMRSYFWLDFQQLNDIYRYKTEEYSHTAVNKFNVIPDSIPEWVFDFMPTRGGYF GSVIVT01034488001
L-KHDQ--EGNEFIERIVKRLHALSYHMRSYFWLDFQQLNDIYRYKTEEYSHTAVNKFNVIPDSIPDWVFDFMPIRGGYF Potri.013G110800
L-KHDE--EGKECIERIVKRLHALSYHMRSYFWLDFQQLNDIYRYKTEEYSHTAVNKFNVIPDSIPDWVFDFMPTRGGYF Potri.019G082000
L-KHDT--EGGEFVERIVKRLHALSYHMRSYFWLDFQQLNDIYRYKTEEYSHTAVNKFNVIPDSIPDWVFEFVPKRGGYF Solyc11g020610
L-KHDT--EGKEFIERIVKRLHALSYHMRSYFWLDFQQLNDIYRYKTEEYSHTAVNKFNVIPDSIPEWVFDFVPTRGGYF Solyc06g065210
L-KHDA--EGKEFMERIVKRLHALSFHMRSYFWLDFQQLNDIYRYKTEEYSHTAVNKFNVIPDSIPEWVFDFMPLRGGYF At1G22650
L-KHDN--EGKEFVEKIATRLHALSYHMRSYFWLDFQQLNDIYRYKTEEYSHTAVNKFNVIPDSIPDWLFDFMPCQGGFF GRMZM2G118737
L-KHDN--EGKEFVEKIATRLHALSYHMRSYFWLDFQQLNDIYRYKTEEYSHTAVNKFNVIPDSIPDWLFDFMPCQGGFF Sb04g022350
L-KHDN--EGKEFVEKIATRLHALSYHMRSYFWLDFQQLNDIYRYKTEEYSHTAVNKFNVIPDSIPDWLFDFMPCQGGFF GRMZM2G170842
L-KHDA--EGKDFVERIATRLHALSYHMRSYFWLDFQQLNDIYRYKTEEYSHTAVNKFNVIPDSIPDWLFDFMPCQGGFF Bradi3g45530
L-KHDN--EGKEFVERIATRLHALSYHMRSYYWLDFQQLNDIYRYKTEEYSHTAVNKFNVIPDSIPDWLFDFMPCQGGFF LOC_Os02g34560
L-KPDA--EGKEIMERIVTRLTALSYHMRSYFWLDFQQLNDIYRFKTEEYSHTAVNKFNVNPESIPDWLFDFMPTRGGYF GRMZM2G136139
L-KPDA--EGKEIMERIVTRLAALSYHMRSYFWLDFQQLNDIYRFKTEEYSHTAVNKFNVNPESIPDWLFDFMPTRGGYF Sb04g029440
L-KPDAP-EGKETMDRVATRLHALTYHMRSYFWLDFQQLNDVYRYRTEEYSHTAVNKFNVIPESIPDWVFDFMPSRGGYF LOC_Os04g35280
L-KPEGE-GNKDTVERIVTRLHALSYHMRAYFWLDFQQLNVIYRFKTEEYSHTAVNKFNVIPESIPDWLLDFMPSKGGYF Bradi5g10357
L-KHDS--EGKEFMEKIVKRLHALSFHMRSYFWLDFQQLNDIYRYKTEEYSHTAVNKFNVIPDSIPDWIFDFMPLRGGYF At1G72000
L-KPEG--EGKDFIEKIGQRLHALTYHMRNYFWLDFHQLNNIYRYKTEEYSHTAVNKFNVIPDSIPDWVFGFMPCRGGYF GRMZM2G007277
L-KPEG--EGKDFIEKIGQRLHALTYHMRNYFWLDFHQLNNIYRYKTEEYSHTAVNKFNVIPDSIPDWVFDFMPCRGGYF Sb05g004770
L-KPEG--EGKDFIEKIGQRLHALTYHMRNYFWLDFHQLNNIYRYKTEEYSHTAVNKFNVIPDSIPDWVFDFMPCRGGYF GRMZM2G115451
L-KPDG--EGKDFIEKIGQRLHALTYHMRNYFWLDFPHLNNIYRYKTEEYSHTAVNKFNVIPDSIPDWVFDFMPCRGGYF LOC_Os11g07440
L-KPDG--EGKEFIEKIGQRLHALTYHMRNYFWLDFPHLNNIYRYKTEEYSHTAVNKFNVIPDSIPDWVFDFMPCRGGYF Bradi4g24470
L-KPEG--AGKEFIERIDKRLHALSYHMRSYFWLDFQQLNDIYRYKTEEYSHTAVNKFNVIPDSLPDWVFDFMPMKGGYF TnS000892793t06
I-KAEG--DGKEFLERIDKRLHALSFHIREYFWLDHQQLNNIYRFKTEEYSHTAVNKFNVIPDSIPDWIFDFLPLKGGFF Pp3c4_10940
I-KAEG--EGKEFLERIDKRLHALSFHMREYFWLDHQQLNNIYRFKTEEYSHTAVNKFNVIPDSIPDWIFDFLPLKGGFF Pp3c12_12810
I-KADG--DGKEFLEKIDKRLHALSYHMREYFWLDHQQLNNIYRFKTEEYSHTAVNKFNVIPDSIPDWIFDFLPLKGGFF Pp3c3_25210
I-KPDG--DGKEFLERIDKRLHALSFHMREYFWLDHQQLNNIYRFKTEEYSHTAVNKFNVIPESIPDWIFDFLPLKGGFF Pp3c10_23170
L-KPEA--GGKEFIERIDKRLHALRYHLRSYFWLDFQQLNNIYRYKTEEYSHTAVNKFNVIPDSIPDWVFDFMPMKGGYF Sm89558
L-KPEG--EGKDFLERVDKRLYALTFHMRNYFWLDHSSLNNIYRYKTEEYSETAVNKFNVIPDSIPDWVMDFMPRKGGYF kfl00164_0180_v1.1
L-KPEG--DGKEFLERIDKRLHALSYHIRTYFWLDHEQLNNIYRYKTEEYSHTAVNKFNVIPDSIPDWVFDFMPMKGGYF TnS000602483t02
L-KPDA--GGKEFLERISKRLHALSFHMRSYFWLDFQQLNNIYRYKTEEYSHTAVNKFNVIPDSIPDWVFDFIPTRGGYF TnS000105403t01
L-KPDA--GGKEFLERISKRLHALSFHMRSYFWLDFQQLNNIYRYKTEEYSHTAVNKFNVIPDSIPDWVLDFMPTRGGYF TnS000982973t03
L-KPEL--GGKEFIERIEKRLVALSYHIRTYFWLDFQNLNNIYRYKTEEYSHTAVNKFNVIPDSIPDWVFDFMPMKGGYF Sm111393
L-KQDAS--TKELIERIDKRLRALRYHMRSYYWLDFEQLNKVYRYKTEEYSQTAVNKFNVIPDSIPDWVFDFVPTNGGYF Azfi_s0064.g035495

```
L-KPEGDNRTKELIEQLDKRLQALSFHIRSYFWLDFEQLNNVYRYKTEEYSHTAVNKFNVIPESIPDWVFDFMPTKGGYF Azfi_s0040.g026676
L-KQEAPG-NKELLERIDKRLNALSYHMRNYFWLDHHQLNSIYRYKTEEYSHTAVNKFNVIPESIPDWVFDFMPMRGGYF Sm151264
L-KAEGDG--KELAERIDKRLHALNFHMRSYFWLDFEQLNNVYRYKTEEYSHTAVNKFNVIPDSIPDWVFDFMPMKGGYF Azfi_s0393.g067806
L-KPDGEG--KELLERVDKRLHALSFHIRSYFWLDFQQLNDVYRYKTEEYSHTAVNKFNVIPDSIPEWVFDFMPCKGGYF Azfi_s0003.g008094
L-KPEEG--GKEFIERIDKRLHALSFHMREYYWLDFEQLNNIYRYKTEEYSHTAVNKFNVIPDSIPDWVFDFMPTKGGYF Azfi_s0059.g034661
L-KPEGG--GKEFIERIDKRLHALSFHMREYYWLDFEQLNNIYRYKTEEYSHTAVNKFNVIPDSIPEWVFDFMPMKGGYF Azfi_s0001.g000547
LPKHDGD--CKEFKARIDKRLDALRFHMREYFWLDFPKLNKIYRYKTEEYSHTAVNKFNVIPDSLPDWVFDFIPMKGGYF Azfi_s0096.g043780
LQEDDG---KEFIMRIEKRLQALTYHMRSYFWLDFQQLNNIYRYKTEEYSHTAVNKFNVIPDSIPDWVFDFMPMKGGYF GSVIVT01037429001
L-KPER--DGKELIERIDKRIMALSFHFRKYYWMDHTQLNIIYRYKTEEYSDTAVNKFNVIPESIPDWVFDFMPLRGGYF Eucgr.D02386.1
L-KPEL--DGKEFIERIEKRITALSYHIQTYYWLDFTQLNNIYRYKTEEYSHTAVNKFNVIPESIPDWVFDFMPLRGGYL Potri.002G173600
L-KPEH--GGKEFFKRIDARITALSFHVQTYYWLDITQLNNIYRYKTEEYSHTAVNKFNIIPDSIPEWLFDFMPLRGGYF GSVIVT01027176001
L-KQES---DADFVNHITKRIQALSYHLHSYYWLDFQRLNDIYRYKTEEYSQTALNKFNVMPESIPDWIFDFMPSRGGYF GRMZM2G477236
L-KQDS---DADFVNHITKRIQALSYHLHSYYWLDFQRLNDIYRYKTEEYSQTALNKFNVIPESIPDWIFDFMPSRGGYF Sb04g002180
L-KDS----NDDFVCQITKRIKALSYHLHSYYWLDFQRLNDIYRYKTEEYSQTALNKFNVIPESIPDWIFDFMPSRGGYF Bradi3g02560
L-KEDH---NDDFVYQISRRIKALSYHLHSYYWLDFQRLNEIYRYKTEEYSETALNKFNVIPESIPDWIFDFMPSRGGYF LOC_Os02g03320
LTPEDG---SADLIRALNNRLVALNFHIREYYWLDLKKINEIYRYQTEEYSYDAVNKFNIYPDQIPSWLVDFMPNRGGYL At5G22510
LTPEDA---SADLIQALNNRLIALSFHIREYYWIDMRKLNEIYRYTTEEYSYDAVNKFNIYPDQIPPWLAGWMPNKGGYL Eucgr.J00457.1
LAPEDG---SADLLRALNNRLVALSFHIREYYWIDLRKLNEIYRYKTEEYSYDAVNKFNIYPDQVSPWLVEWMPNQGGYL Potri.008G024100
LAPEDG---SADLIRALNNRLVALSFHIREYYWIDLRKLNEIYRYKTEEYSYDAVNKFNIYPDQISPWLVEWMPNQGGYL Potri.010G236100
LAPEDG---SADLIRALNNRLVALSFHIREYYWIDMKKLNEIYRYKTEEYSYDAVNKFNIYPDQISPWLVEWMPNKGGYL GSVIVT01034753001
LAPEDG---SADLIRALNNRLVALSFHIREYYWIDLRKLNEIYRYKTEEYSYDAVNKFNIYPDQIPPWLVEWIPNRGGYL Eucgr.G02704.1
LAPEDG---SSALIRALNNRVVALSFHIREYYWIDMRKLNEIYRYKTEEYSYDAVNKFNIYPDQIPPWLVEWMPSKGGYL GSVIVT01031374001
LAPEDG---SADLIRALNNRLVALSFHIREYYWIDLKKLNEIYRYTTEEYSYDAVNKFNIYPDQIPPWLVEFMPNKGGYL Potri.004G186500
LTPEDG---SADLIRALNNRLVALSFHIREYYWIDMKKLNEIYRYQTEEYSYDAVNKFNIYPDQISPWLVDWMPSKGGYL Solyc11g007270
LTQEDG---SADLIRALNNRLIALSFHIREYYWLDMQKLNEIYRYKTEEYSYDAVNKFNIYPDQISPWLVEWIPPKGGYF GRMZM2G040843
LAQEDG---SADLIRALNNRLIALSFHIREYYWLDMQKLNEIYRYKTEEYSYDAVNKFNIYPDQISPWLVEWIPPKGGYF Sb04g021550
LTPEDG---SADLIRALNNRLIALSFHIREYYWVDMQKLNEIYRYKTEEYSYDAVNKFNIYPDQVSPWLVEWIPPKGGYF Bradi3g44770
LTPEDG---SADLIRALNNRLIALSFHIREYYWVDMQKLNEIYRYKTEEYSYDAVNKFNIYPDQVSPWLVEWIPPKGGYF LOC_Os02g32730
LTPEDG---SADLIRALNSRLMALSFHIREYYWLDKRKLNEIYRYKTEEYSYDAVNKFNIYPDQIPSWLVEWIPPKGGYF Bradi5g09200
LTPEDG---SADLIRALNSRLIALSFHIREYYWLDKRKLNEIYRYKTEEYSYDAVNKFNIYPDQIPPWLVEWIPPKGGYF LOC_Os04g33490
LAPEEA---STDLVRALNNRLLALSFHIREYYWIDVKKLNEIYRYKTEEYSYDAINKFNIYPDQIPPWLVEWMPSEGGYL Solyc11g067050
LAPEDG---SADLIRALTNRLIALSFHIREYYWIDMRKLNEIYRYKTEEYSYDAVNKFNIYPDQISPWLVEWMPDRGGYF TnS000875687t08
LISEDS---ALDLIRTLTSRLSALSFHIREYYWVDMGKLNEIYRYKTEEYSHEAVNKFNIYPDHLSPWLVDWIPNKGGYL Sm267827
LTPEDG---SLDLIRAMINRLIALSFHIREYYWVDTKKLNEIYRYKTEEYSYDAVNKFNIYPDQIPPWLVEWMPDRGGYL Azfi_s0345.g065806
LLPEEG---AQDLIRQINSRLAALSFHIQEYYWLDIVKLNEIYRYKTEEYSTEAVNKFNIYPEQVSQWLLDWLPESGGYF Pp3c6_27130
LVPEDG---AGDLIRQINARLAALSFHIQEYYWLDISKVNEIYRYKTEEYSTEAVNKFNIYPEQVSQWLLDWMPETGGYF Pp3c16_15060
LHPE-----AHDLIRSVNSRLAALSFHIQEYYWLDIRKLNEIYRYRTEEYSSDAVNKFNIYPDQISRWLLDWLPEQGGYF Pp3c17_13250
LIPDEK---SHQLLTAVNSRLSALSFHIREYYWLDIAKLNEIYRYKTEEYSHEAVNKFNIYPEQIPDWLADWMPDHGGYF Sm443960
LTPED----NQGLLNAITARLSALSFHLRQYYWLDFERLNEIYRYQTEEYSEDAANKFNVYPDSLPTWLMDWMPEKGGYF kfl00563_0120_v1.1
```

```
IGNVSPAKMDFRWFCLGNCIAILSSLATPEQSTAIMDLIESRWEELVGEMPLKVIYPAIESHEWRIVTGCDPKNTRWSYH Potri.004G167500
IGNVSPARMDFRWFCLGNCIAILSSLATPEQSTAIMDLIESRWEELVGEMPLKVIYPAIESHEWRIVTGCDPKNTRWSYH Potri.009G129000
VGNVSPARMDFRWFCLGNCIAILSSLATPEQSTAIMDLIESRWEELVGEMPLKVCYPAIENHEWKIVTGCDPKNTRWSYH Eucgr.G01751.1
IGNVSPARMDFRWFALGNCIAILSSLATPEQSTAIMDLIESRWEELVGEMPLKVCYPAIESHEWRIVTGCDPKNTRWSYH At4G34860
IGNVSPARMDFRWFCLGNCVAILSSLATPEQSTAIMDLIESRWEELVGDMPLKVCYPAIEGHEWRIVTGCDPKNTRWSYH GSVIVT01024105001
LGNVGPSNMDFRWFCLGNCIAILSSLATPEQATKIMDLIDSRWHELVGEMPLKVCYPAIEGHEWRIVTGCDPKNTRWSYH Solyc01g111100
IGNVSPARMDFRWFCLGNCVAILSSLATPEQSAAIMDLIEERWEELVGEMPLKICYPAIESHEWTIVTGCDPKNTRWSYH Eucgr.F01661.1
IGNVSPARMDFRWFCLGNCVAILSSLATPEQASAIMDLIESRWEELVGEMPLKICYPALESHEWRTVTGCDPKNTRWSYH Potri.005G239400
IGNVSPAKMDFRWFCLGNCVAILSSLATPEQSSAIMDLIESRWQELVGEMPLKICYPAFESHEWRIVTGCDPKNTRWSYH GSVIVT01009904001
IGNVSPAHMDFRWFCLGNCISILSSLATPEQASAIMDLVESRWQELVGEMPLKICYPAMEGHEWRIVTGCDPKNTSWSYH Solyc04g081440
VGNVGPAHMDFRWFALGNCVSILSSLATPDQSMAIMDLLEHRWAELVGEMPLKICYPCLEGHEWRIVTGCDPKNTRWSYH At1G35580
VGNVSPARMDFRWFSLGNCVSILSSLATPDQSMAIMDLLEHRWEELVGEMPLKICYPCIESHEWRIVTGCDPKNTRWSYH At4G09510
IGNVSPARMDFRWFALGNCVAILASLATPEQSMAIMDLIEARWEELVGEMPLKISYPALEGHEWRIVTGCDPKNTRWSYH Eucgr.F02588.1
IGNVSPARMDFRWFALGNCVAILSSLATPEQSMAIMDLIESRWEELVGEMPLKISYPAFENHEWRIITGCDPKNTRWSYH GSVIVT01034488001
IGNVSPARMDFRWFALGNCIAILSSLATHEQAMAIMDLIEARWEELVGEMPLKIAYPAIESHEWRIVTGCDPKNTRWSYH Potri.013G110800
IGNVSPARMDFRWFALGNCIAILSSLATHEQAMAIMDLIEARWEELVGEMPLKIAYPAIESHEWRIVTGCDPKNTRWSYH Potri.019G082000
VGNVSPARMDFRWFALGNCIAILSSLATPEQASAIMDLIEARWDELVAEMPLKISYPALENHDWRHITGCDPKNTRWSYH Solyc11g020610
IGNVSPARMDFRWFALGNCIAILSSLATPEQASAIMDLIESRWEELVADMPLKICYPAIENHEWRIVTGCDPKNIRWSYH Solyc06g065210
IGNVSPARMDFRWFALGNCVAILASLATPEQSASIMDLIEERWEELVGEMPVKICHPAIESHEWRIVTGCDPKNTRWSYH At1G22650
IGNVSPARMDFRWFALGNMIAILSSLATPEQSVAIMDLIEERWEELIGEMPLKICYPAIENHEWRIVTGCDPKNTRWSYH GRMZM2G118737
IGNVSPARMDFRWFALGNMIAILSSLATPEQSVAIMDLIEERWEELIGDMPLKICYPAIENHEWRIVTGCDPKNTRWSYH Sb04g022350
VGNVSPARMDFRWFALGNMIAILSSLATPEQSNAIMDLIEERWEELIGEMPLKICYPAIENHEWRIVTGCDPKNTRWSYH GRMZM2G170842
VGNVSPARMDFRWFALGNMIAIVSSLATPEQSMAIMDLIEERWEELIGEMPLKICYPAIENHEWRIVTGCDPKNTRWSYH Bradi3g45530
IGNVSPARMDFRWFALGNMIAILSSLATPEQSTAIMDLIEERWEELIGEMPLKICYPAIENHEWRIVTGCDPKNTRWSYH LOC_Os02g34560
VGNVSPARMDFRWFALGNCVAILASLATPDQAAAIMDLIEERWEDLVGEMPVKICYPAIEGHEWQIVTGCDPKNTRWSYH GRMZM2G136139
VGNVSPARMDFRWFALGNCVAILASLATPDQAAAIMDLIEERWEDLVGEMPVKICYPAIEGHEWQIVTGCDPKNTRWSYH Sb04g029440
VGNVSPARMDFRWFALGNFVAILASMATPEQAAAIMDLIEERWEDLIGEMPLKISFPAIESHEWEFVTGCDPKNTRWSYH LOC_Os04g35280
VGNVSPARMDFRWFALGNCVAILASLATPDQAAAIMDLIEERWEDLVGEMPLKICYPAIEGHEWQSVTGCDPKNTRWSYH Bradi5g10357
VGNVSPARMDFRWFALGNCIAILSSLATPEQSMAIMDLIEARWEELVGEMPLKICYPAMESHEWGIVTGCDPKNTRWSYH At1G72000
LGNVSPAMMDFRWFALGNCIAIVSSLATPEQSVAIMDLIEEKWDELVGEMPLKICYPALENHEWRIITGCDPKNTRWSYH GRMZM2G007277
LGNVSPAMMDFRWFALGNCIAIVSSLATPEQSVAIMDLIEEKWDELVGEMPLKICYPALENHEWRIITGCDPKNTRWSYH Sb05g004770
LGNVSPAMMDFRWFALGNCIAIVSSLATPEQSVAIMDLIEEKWDELVGEMPLKICYPALENHEWRIITGCDPKNTRWSYH GRMZM2G115451
LGNVSPAMMDFRWFALGNCIAIISSLATPEQSVAIMDLIEERWEELVGEMPLKICYPAIENHEWRIITGCDPKNTRWSYH LOC_Os11g07440
LGNVSPAMMDFRWFALGNCIAIISSLATPEQSSAIMDLIEERWDELVGEMPLKISYPAIENHEWRIITGCDPKNTRWSYH Bradi4g24470
IGNVSPARMDFRWFALGNCIAILSCLATPEQSVAIMDLIEERWDELVGEMPLKVAYPALENHEWRIITGCDPKNTRWSYH TnS000892793t06
IGNVSPARMDFRWFAIGNFMAILSSLATSEQASAIMDLLEARWTELVGDMPLKVSYPAMEGHEWRIVTGCDPKNTRWSYH Pp3c4_10940
IGNVSPARMDFRWFAIGNFMAILASLATSEQAAAIMDLLEARWPELVGDMPLKVSYPAMEGHEWRVVTGCDPKNTRWSYH Pp3c12_12810
IGNVSPARMDFRWFAIGNFMAILGSLATADQASAIMDLLEARWPELVGDMPLKVSYPAMEGHEWRIITGCDPKNTRWSYH Pp3c3_25210
IGNVSPARMDFRWFAIGNFMAILGSLATFDQASAIMDLLEARWPELVGDMPLKVTYPAMEGHEWRIITGCDPKNTRWSYH Pp3c10_23170
IGNVSPARMDFRWFTIGNCVAILSSLTTPEQATAIMDLIEERWDDLIGEMPLKVTYPALEGHEWKIVTGCDPKNTRWSYH Sm89558
IGNVSPARMDFRWFAIGNCMAIISSLATTEQANMIMDLIEARWDELVGEMPMKATYPALEGHEWRLITGCDPKNTRWSYH kfl00164_0180_v1.1
VGNVSPARLDFRWFSLGNCVAILTSLATPEQASAIMDLFEERWEDLIGDMPLKIAYPALENHEWRIVTGCDPKNTRWSYH TnS000602483t02
VGNVSPARIDFRWFCLGNCIAILSSLATSEQSTRIMELFEERWTELIGEMPLKVSYPALEGHEWQIVTGCDPKNTRWSYH TnS000105403t01
VENVSPARIDFRWFCLGNCIAILSSLATPEQSTKIMDLFEERWTELVGEMPLKVSYPALEGHEWRIVTGCDPKNTRWSYH TnS000982973t03
IGNVSPARMDFRWFLIGNCVAILSSIATPEQASAIMDLVEARWADLVGEMPLKISYPAMDAEEWVIVTGCDPKNTRWSYH Sm111393
IGNVSPARMDFRWFCLGNCVAILSSLATPSQEEGIMDLFEERWEELIGELPLKITYPALENHEWRIETGCDPKNTRWSYH Azfi_s0064.g035495
```

```
IGNVSPARMDFRWFCMGNCTAILASLATPEQEEGIMDLFEERWEDLIGEMPLKISYPALENHEWRIVTGCDPKNTRWSYH Azfi_s0040.g026676
IGNVSPARMDFRWFLLGNCVAILSSMATSEQAEAIMDLIEERWEDLVGEMPLKISYPAIEGHEWRIVTGFDPKNTRWSYH Sm151264
IGNVSPARMDFRWFCLGNCIAILSSLATPEQEEGIMDLLEERWQDLIGEMPLKIAYPALEGHEWKIVTGCDPKNTRWSYH Azfi_s0393.g067806
IGNVSPARMDFRWFCLGNCVAILSSLATPEQEEGIMDLLEERWQDLIGEMPLKIAYPALEGHEWTIVTGCDPKNTRWSYH Azfi_s0003.g008094
IGNVSPACMDFRWFCLGNCVAILSSLATPTQEAAIMDLFEERWQDLVGEMPLKVAYPALENHEWRIITGCDPKNTRWSYH Azfi_s0059.g034661
IGNVSPARMDFRWFCVGNCVAILSSLATPAQEAAIMDLFEERWEDLVGEMPLKVAYPALENHEWRIVTGCDPKNTRWSYH Azfi_s0001.g000547
IANVGPSRKDFRWFCIGNCIAILSSLATPQQEVAIMDLFEERWEYLIGEMPLKLSYPALENHEWRIITGCDPKNTRWSYH Azfi_s0096.g043780
IANVSPARMDFRWFVLGNCVAILSSLATHNQSMAILDLIEERWGELVGKMPLKLSYPALDIHGWSIETGSDPKNTRWSYH GSVIVT01037429001
IGNVSPARMDFRWFLVGNCIAILSSLATPAQATAIMELIEDRWEDLIGEMPLKITYPALEGHEWKIVTGCDPKNTRWSYH Eucgr.D02386.1
IGNVSPARMDFRWFLVGNCVAILSSLVTPAQATAIMDLVEERWEDLIGEMPLKITYPALEGHEWRLVTGFDPKNTRWSYH Potri.002G173600
MGNVSPGRMDFRWFLAGNCIAILSSLATSEQATAIMDLVEERWEQLIGEVPLKVVYPALEGHYWELVTGCDPKNTPWSYH GSVIVT01027176001
IGNVSPARMDFRWFCLGNFIAILSSLATGEQAEAILDLVEERWQELIGEMPLKICYPAMENQEWQIVTGCDPKNTRWSYH GRMZM2G477236
IGNVSPARMDFRWFCLGNFIAILSSLATGEQAEAILDLVEERWQELIGEMPLKICYPAMENQEWQIVTGCDPKNTRWSYH Sb04g002180
IGNVSPARMDFRWFCLGNFIAILSSLATGEQAEAILDLVEERWQELIGEMPMKICYPAMENQEWQIVTGCDPKNTRWSYH Bradi3g02560
IGNVSPARMDFRWFCLGNFIAILSSLTTGEQAEAILDLVEERWEELIGEMPMKVCYPAMENQEWQIVTGCDPKNTRWSYH LOC_Os02g03320
IGNLQPAHMDFRFFTLGNLWSIVSSLASNDQSHAILDFIEAKWAELVADMPLKICYPAMEGEEWRIITGSDPKNTPWSYH At5G22510
IGNLQPAHMDFRFFSLGNLWSIVSSLATVDQSHAILDLIEAKWADLVADMPFKICYPALEGQEWRIVTGSDPKNTPWSYH Eucgr.J00457.1
IGNLQPAHMDFRFFSLGNIWSVVSGLATRDQSNAILDLIEAKWSDLVADMPLKICYPALEGQEWQIITGSDPKNTPWSYH Potri.008G024100
IGNLQPAHMDFRFFSLGNIWSIVSGLATRDQSNAILDFIEAKWSDLIADMPLKICYPALEGQEWQIITGSDPKNTPWSYH Potri.010G236100
IGNLQPAHMDFRFFSLGNLWSIISSLATMDQSHAILDLVEAKWGDLVADMPLKICYPALEGQEWQIITGSDPKNTPWSYH GSVIVT01034753001
IGNLQPAHMDFRFFSLGNFWSIVSGLATVDQSHAILDLIEAKWADLVSDMPLKICYPALEGQEWQIITGSDPKNTPWSYH Eucgr.G02704.1
IGNLQPAHMDFRFFSLGNLWSIVSSLATTDQSHAMLDLIEAKWSELVADMPFKICYPAFEVLGHTIMEARGQLCSGRTKS GSVIVT01031374001
IGNLQPAHMDFRFFTLGNLWSIVSSLATLDQSHAILDLIEAKWAELVAEMPIKICYPALEGQEWRIVTGSDPKNTAWSYH Potri.004G186500
IGNLQPAHMDFRFFSLGNLWSIVCSLTTDDQSHAILDLIEAKWTDLVADMPFKICYPALEGQEWKIITGCDPKNTPWSYH Solyc11g007270
IGNLQPAHMDFRFFSLGNLWSIVSSLATTHQSHAILDLIESKWSDLVAEMPLKICYPALENQEWKIITGSDPKNTPWSYH GRMZM2G040843
IGNLQPAHMDFRFFSLGNLWSIVSSLATTHQSHAILDLIESKWSDLVAEMPLKICYPALENQEWKIITGSDPKNTPWSYH Sb04g021550
IGNLQPAHMDFRFFALGNLWSIVSSLATTHQSHAILDLIESKWSDLVAEMPLKICYPALENQEWKIITGSDPKNTPWSYH Bradi3g44770
IGNLQPAHMDFRFFSLGNLWSIVSSLATTHQSHAILDLIESKWSDLVAEMPLKICYPALENQEWKIITGSDPKNTPWSYH LOC_Os02g32730
IGNLQPAHMDFRFFSLGNLWSIVSSLATTHQSHAILDLVEAKWSDLVAEMPMKICHPALEGQEWKFITGSDPKNTPWSYH Bradi5g09200
IGNLQPAHMDFRFFSLGNLWSIVSSLATSHQSDAILDLVEAKWSDLVADMPMKICYPALEDQEWKFITGSDPKNTAWSYH LOC_Os04g33490
IGNLQPAHMDFRFFSLGNVWSIVSSLANIDQSHAILDLIEAKWEDLVADMPLKICYPALEGQEWRIITGGDPKNTPWSYH Solyc11g067050
IGNLQPAHMEFRFFSLGNLWSIVCNLATNEQSNAILDLIEAKWNDLIAGMPLKICYPALEGPEWQIITGCDPKNTPWSYH TnS000875687t08
VGNLQPAHMDFRFFSLGNLWAIVSSLATPEQAEGILDLIEARWVDFVGNMPMKICYPALQGEEWRIITGSDPKNTLLWLA Sm267827
IGNLQPAHMDFRFFSLGNLWSIVGSLSTPEQSNAILDLIEAKWTDLIANMPLKICYPALEGEEWRIITGSDPKNT----- Azfi_s0345.g065806
IGNLQPAHMDFRWFTLGNMWSICGGLATPKQSENILRLVESKWQDLVATMPIKICFPALTDEEWRIITGADPKNTAWSYH Pp3c6_27130
IGNLQPAHMDFRWFALGNMWSICSGLATENQAEDILKLVESKWDDLIATMPMKICFPALTEDEWRIITGADPKNTAWSYH Pp3c16_15060
IGNLQPAHMDFRWFTLGNIWSICSALATKEQSEEILTLVEKKWEDLIGTMPLKICYPTLEEDEWRIITGADPKNTAWSYH Pp3c17_13250
IGNLQPAHMDFRFFSLGNLWAIVSSLATPQQSSGILDLIQDRWKHLVGSMPLKICFPAFENEEWRIITGGDPKNTAWSYH Sm443960
IGGVAPAQLDFRVFTLGNCWAILSSLATREQTGAILDLYEQKWVDLIADMPLKICYPAMVGEEWRIVTGCDPKNTAWSYH kfl00563_0120_v1.1
```

```
NGGSWP------------------VLLWLLTAACIKTGRPQIARRAIELAETRLVKDNWPEYYDGKLGRFVGKQARKFQT Potri.004G167500
NGGSWP------------------VLLWLLTAACIKTGRPQIARRAIELAETRLIKDNWPEYYDGKLGRFIGKQARKSQT Potri.009G129000
NGGSWP------------------VLLWLLTAACIKTGRPLIARRAIELAEARLLKDNWPEYYDGKLGCYIGKQARKFQT Eucgr.G01751.1
NGGSWP------------------VLLWLLTAACIKTGRPQIARRAIEVAEARLHKDHWPEYYDGKVGRYVGKQSRKNQT At4G34860
NGGSWP------------------VLLWLLTAACIKTGRPQIARRAIELAESRLVKDSWPEYYDGKLGRFIGKQARKFQT GSVIVT01024105001
NGGSWP------------------VLLWLLTAACIKTGRPQIARRAIELAEQRLSKDGWPEYYDGKLGRFIGKQARKHQT Solyc01g111100
NGGSWP------------------VLLWLLTAACIKTGRPQIARRAIELIESRLSKDHWPEYYDGKLGKYVGKQARKFQT Eucgr.F01661.1
NGGSWP------------------VLLWLLTAACIKTGRPQIARRAIELAESRLSKDHWPEYYDGKLGLYVGKQARKFQT Potri.005G239400
NGGSWP------------------VLIWLLTAACIKTGRPQIARRAIELAESRLLKDNWPEYYDGKLGRYIGKQARKFQT GSVIVT01009904001
NGGTWP------------------VLLWLLTAAAIKTGRPQIARRAIELAESRLLKDSWPEYYDGKLGRFIGKQARKFQT Solyc04g081440
NGGSWP------------------VLLWQLTAACIKTGRPQIARRAVDLIESRLHRDCWPEYYDGKLGRYVGKQARKYQT At1G35580
NGGSWP------------------VLLWTLTAACIKTGRPQIARRAIDLIESRLHRDCWPEYYDGKQGRYVGKQARKYQT At4G09510
NGGSWP------------------VLLWLLTAACIKTGRPQIARRAIDLAETRLLKDSWPEYYDGTLGRYIGKQARKYQT Eucgr.F02588.1
NGGSWP------------------VLLWLLTAACIKTGRPQIARRAIDLAESRLLKDSWPEYYDGKLGRYVGKQARKYQT GSVIVT01034488001
NGGSWP------------------VLLWLLTAACIKTGRPQIARKAIDLAETRLLKDGWPEYYDGKLGRYVGKQARKYQT Potri.013G110800
NGGSWP------------------VLLWLLTAACIKTGRPQIARKAIDLAETRLLKDSWPEYYDGKLGRYIGKQARKYQT Potri.019G082000
NGGSWP------------------VLLWLLTAACIKTGRPQIARRAIDLAESRLSKDSWPEYYDGTVGRYIGKQARKYQT Solyc11g020610
NGGSWP------------------VLLWLLTAACIKTGRPQIARRAIDLAESRLLKDSWPEYYDGKLGRYIGKQARKHQT Solyc06g065210
NGGSWP------------------VLLWLLTAACIKTGRPQIARRAIDLAEARLLKDGWPEYYDGKSGRFIGKQARKFQT At1G22650
NGGSWP------------------VLLWLLTAACIKTGRPQIARRAIDLAERRLLKDGWPEYYDGKLGRYVGKQARKFQT GRMZM2G118737
NGGSWP------------------VLLWLLTAACIKTGRPQIARRAIDLAERRLLKDGWPEYYDGKLGRYVGKQARKFQT Sb04g022350
NGGSWP------------------VLLWLLTAACIKTGRPQIARRAIDLAERRLLKDGWPEYYDGKLGRYVGKQARKFQT GRMZM2G170842
NGGSWP------------------VLLWLLTAASIKTGRPQIARRAIDLAERRLLKDGWPEYYDGKLGKYVGKQARKFQT Bradi3g45530
NGGSWP------------------VLLWLLTAACIKTGRPQIARRAIDLAERRLLKDGWPEYYDGKLGRYVGKQARKFQT LOC_Os02g34560
NGGSWP------------------VLLWLLTAACIKTGRLKIARRAIDLAEARLARDGWPEYYDGKLGRYIGKQARKLQT GRMZM2G136139
NGGSWP------------------VLLWLLTAACIKTGRLKIARRAIDLAEARLARDGWPEYYDGKLGRYIGKQARKFQT Sb04g029440
NGGSWP------------------VLLWLLTAACIKTGRLKIARRAIELAEARLARDGWPEYYDGKLGRYVGKQARKLQT LOC_Os04g35280
NGGSWP------------------VLLWLLTAACIKTGRLKIARRAIDLAEARLAKDSWPEYYDGKLGRYVGKQARKHQT Bradi5g10357
NGGSWP------------------VLLWLLTAASIKTGRPQIARRAIELAEARLLKDGWPEYYDGKSGRFIGKQARKSQT At1G72000
NGGSWP------------------VLLWLLTAACIKTGRPQMAKRAIELAESRLLKDGWPEYYDGKLGRFVGKQARKFQT GRMZM2G007277
NGGSWP------------------VLLWLLTAACIKTGRPQMAKRAIELAESRLLKDGWPEYYDGKLGRFVGKQARKFQT Sb05g004770
NGGSWP------------------VLLWLLTAACIKTGRPQMAKRAIELAESRLLKDGWPEYYDGKLGRFVGKQARKFQT GRMZM2G115451
NGGSWP------------------VLLWLLTAACIKTGRPQMAKRAIELAESRLLKDGWPEYYDGKLGRFIGKQARKFQT LOC_Os11g07440
NGGSWP------------------VLLWLLTAACIKTGRPQMAKRAIELSEARLLKDGWPEYYDGKLGRFIGKQARKFQT Bradi4g24470
NGGSWP------------------VLLWLLTAACIKTGRPHIAKRAIELAERRLSKDGWPEYYDGKLGRYIGKQARKYQT TnS000892793t06
NAGSWP------------------VILWMLTAACIKAGRPQIARRAIEQVETRLAKDGWPEYYDGKLGRYVGKQARKFQT Pp3c4_10940
NAGSWP------------------VILWMLTAACIKTGRPQIARRAIEQVETRLAKDGWPEYYDGKLGRYVGKQARKFQT Pp3c12_12810
NAGSWP------------------VILWMLTAACIKAGRPQIARRAIEQVETRLSADGWPEYYDGKLGRYVGKQARKFQT Pp3c3_25210
NAGSWP------------------VILWMLTAACIKAGRPQIARRAIEQVETRLSADGWPEYYDGKLGRYVGKQARKFQT Pp3c10_23170
NGGSWP------------------VLLWLLTAACIKTGRPQIARRAIEIAEARLSKDGWPEYYDGKTGRYIGKQARKLQT Sm89558
NGGSWP------------------VISWMLTAAAIKVGRPHVAKKAVDLLEEVLGKDGWPEYYDGKSGQFIGKQARKNQT kfl00164_0180_v1.1
NGGSWP------------------VLLWLLTAACIKTGRPQIARKAIELIETRLAQDGWPEYYDGKLGRYIGKQARRHQT TnS000602483t02
NGGSWP------------------VLLWLFTAACVKAGRPQMARRAIELAEVRLSKDGWPEYYDGKLGRYVGKQSRKWQT TnS000105403t01
NGGSWPETVSKTALLILLIFEFGVVLLWLFTAACVKAGRPQMARQAIELAEVRLSKDGWPEYYDGKLGRYIGKQSRKWQT TnS000982973t03
NGGTWP------------------VLLWMLTAASIKTGRPQIARRAIELAEPRLCKEGWPEYYDGKLGRYVGKQARKFQT Sm111393
NGGSWP------------------VLVWLFTAACIKAGRPQMAERAIKLLETRLSKDSWPEYYDGKLGRYVGKQARKYQT Azfi_s0064.g035495
```

```
NGGSWP------------------VLMWLFTAACVKAGRPQMAARAMSRLEVRLGKEGWAEYYDGKSGRYVGKQARKFQT Azfi_s0040.g026676
NGGSWP------------------VLLWLLTAACVKTGRPQMARRAIEQAEQRLSLDGWPEYYDGKLGRYIGKQARKLQT Sm151264
NGGSWP------------------VLIWLFTAACIKTGRPHMARRAIELLESRLSKDHWPEYYDGTTGRYIGKQARKFQT Azfi_s0393.g067806
NGGSWP------------------VLIWLLTAACIKTGRPHIARRAIELLESRLSKDHWPEYYDSKLGRYIGKQARKFQT Azfi_s0003.g008094
NGGSWPV------------------LLWLFTAVCIKTGRPQMARRAIEIAEQRLSNDHWPEYYDGKQGRFIGKQARKMQT Azfi_s0059.g034661
NGGSWPV------------------LLWLFTAACIKTGRPQIARKAIELAEQRLSKDHWPEYYDGKLGRYIGKQARKMQT Azfi_s0001.g000547
NGGSWP-------------------------ATCIKTGRPQLARRAIELAEKRLSKDNWPEYYDGKLGRYVGKQARRLQT Azfi_s0096.g043780
NGGSWP------------------GLLWLVTAACIKTGRPEIARKAIELAEQRLSKDDWQEYYDGKEGCYVGKQSRRLQT GSVIVT01037429001
NGGSWP------------------GKLQLLP------------------------------------------------- Eucgr.D02386.1
NGGSWP------------------MLLWLLSAACIKVGRPQIAKRAIELAEQRLSKDGWPEYYDGKTGRYVGKQARKYQT Potri.002G173600
NGGSWP------------------VLLWLLTAACIKIGRPQIAKRAIDLVEQRLSKDGWPEYYDGKTGRYVGKQARKFQT GSVIVT01027176001
NGGSWP------------------VLLWLLVAVSVKLGRPHLARRAVELMEQRLAKDDFPEYYDGKAGRYVGKQARKFQT GRMZM2G477236
NGGSWP------------------VLLWLLVAVSVKLGRPHLARRAVELMEQRLAKDDFPEYYDGKAGRYVGKQARKFQT Sb04g002180
NAGSWP------------------VLLWLLVAVSVKLGRPHIARRAVELMEKRLAKDEFPEYYDGRAGRYVGKQARKHQT Bradi3g02560
NGGSWP------------------VLLWLLVAVSVKLGRPHIARRAVEVMEKRLVKDEFPEYYDGKAGRYVGKQARKFQT LOC_Os02g03320
NGGAWPTLL------------------WQLTVASIKMGRPEIAEKAVELAERRISLDKWPEYYDTKRARFIGKQARLYQT At5G22510
NGGSWPTLL------------------WQLTVACIKMNRPEIAENAIRMAERRISQDKWPEYYDTKRARFIGKQAHLFQT Eucgr.J00457.1
NAGSWPTLL------------------WQLTVACIKMNRPEIAARAVDIAEKRISRDKWPEYYDTKKARFIGKQARLFQT Potri.008G024100
NAGSWPTLL------------------WQLTAACIKMNRPELAARAVEIAEKRISRDKWPEYYDTKKARFIGKQAHLFQT Potri.010G236100
NAGSWPTLL------------------WQLTVACIKMDRPQIAAKAVEIAERRIARDKWPEYYDTKKARFIGKQACLFQT GSVIVT01034753001
NAGSWPTLL------------------WQLTVACIKMNRADIAAKAVEIVERRISRDRWPEYYDTKRSRFIGKQARLFQT Eucgr.G02704.1
DAKLINIIVNS----------------ELLTVACIKMNRPEIAEKAVKIAEKRISRDKWPEYYDTKQGRFIGKQARLFQT GSVIVT01031374001
NGGSWPTLL------------------WQLTVACIKMNRPEIAERAVQLVERRISRDKWPEYYDTKRARFIGKQAHLFQT Potri.004G186500
NGGSWPTLL------------------WQLAVASIKMNRPEIAAKAVEVAEKRISQDKWPEYYDTKKARFIGKQARLFQT Solyc11g007270
NGGSWPTLL------------------WQLTVACIKMNRPELAAKAIEVAERRIATDKWPEYYDTKRARFIGKQSRLYQT GRMZM2G040843
NGGSWPTLL------------------WQLTVACIKMNRPELAAKAIEVAERRIATDKWPEYYDTKRARFIGKQARLYQT Sb04g021550
NGGSWPTLL------------------WQLTVACIKMNRPEIAAKAVEIAERRIATDKWPEYYDTKRARFIGKQSRLYQT Bradi3g44770
NGGSWPTLL------------------WQLTVASIKMNRPEIAAKAVEVAERRIAIDKWPEYYDTKRARFIGKQSRLYQT LOC_Os02g32730
NGGSWPTLL------------------WQLTVACIKMDRPEIAARAVEVAERRISSDKWPEYYDTKRARFIGKQARLFQT Bradi5g09200
NGGSWPTLL------------------WQLTVACIKVDRSEIAAKAVEVAERRIANDKWPEYYDTKRARFIGKQSRLFQT LOC_Os04g33490
NGGSWPTLL------------------WQLTVACIKMKRPEIAEKAIKIAERRLSRDRWPEYYDTRRGGFIGKQARLFQT Solyc11g067050
NGGSWPTLL------------------WQLTVACIKMKRPEIARQAIAVAEKRLSRDRWPEYYDTKKARFIGKQARLYQT TnS000875687t08
SRWGARRWL-------------------SVPLKLRRNGSPETDGQSIMTQEQ---------------------------- Sm267827
-------------------------------------------------------------------------------- Azfi_s0345.g065806
NGGSWPTLL------------------WQFTVACIKMNRADLAERAVEIAEKRLSRDRWPEYYDTKKGRFIGKQARLYQT Pp3c6_27130
NGGSWPTLL------------------WQFTTACIKMNRPDLAERAIEIAEKRLSRDRWPEYYDTKKGRFIGKQARLYQT Pp3c16_15060
NGGSWPVLL------------------WQFTLACIKMGRSDLAEKAVAIAEKRLSKDWWPEYYDTKSGRFIGKQARLYQT Pp3c17_13250
NG------------------------------ACIKMGRSEVAYEALEIMERRISRDRWPEYYDSRTGKFIGKQSRLFQT Sm443960
NGGSWPTLL------------------WHFTAACIKGGRPELAQRALDQAEKKLAQLGWPEYFDGVKGNYVGKQARKFQT kfl00563_0120_v1.1
```

WSIAGYLVAKMLLEDPSHLGMVALEE-DKQ-MKPPMRRSHSWTF--------------	Potri.004G167500
WSIAGYLVAKMMLEDPSHLGTVALEE-DKQ-MKPPIRRSNSWTF--------------	Potri.009G129000
WSIAGYLVAKMMLEDPSHIGMVSLEE-DRQ-MKPVMRRSNSWTF--------------	Eucgr.G01751.1
WSVAGYLVAKMMLEDPSHVGMVCLEE-DKQ-MKPVMRRSNSWTC--------------	At4G34860
WSVAGYLVAKMMLEDPSHLGMISLEE-DKQ-MKPLIKRSASWTF--------------	GSVIVT01024105001
WSIAGYLVAKMMLEDPSHLGMISLEE-DKQ-LKPVLKRSASF----------------	Solyc01g111100
WSIAGYLVAKMMLEDPSHLGMVSLEE-DRQ-LKPLLKRSASWTC--------------	Eucgr.F01661.1
WSIAGYLVAKMMLEDPSHLGMISLEE-DKQ-MTHLVKRSASWTC--------------	Potri.005G239400
WSIAGYLVAKMMLDDPSHLGMISLEE-DKQ-LKPLFKRSLSWSH--------------	GSVIVT01009904001
WSIAGYLVARMMLEDPSHLGMISLEE-DKQ-MKPTMKRSASWTC--------------	Solyc04g081440
WSIAGYLVAKMLLEDPSHIGMISLEE-DKL-MKPVIKRSASWPQL-------------	At1G35580
WSIAGYLVAKMMLEDPSHIGMISLEE-DKQ-MKPVIKRSASWTC--------------	At4G09510
WSIAGYLVAKMMLEDPSHLGMISLEE-DKQ-MKPVLKRSSSWTC--------------	Eucgr.F02588.1
WSIAGYLVAKMLLEDPSHLGMISLEE-DRQ-MKPLIKRSSSWTC--------------	GSVIVT01034488001
WSIAGYLVAKMMLEDPSHLGMISLEE-DRQ-MKPVLRRSSSWTC--------------	Potri.013G110800
WSIAGYLVAKMMLEDPSHLGMISLEE-DKQ-MNPVLKRSSSWTC--------------	Potri.019G082000
WSIAGYLVAKMLLEDPSHLGMIALEE-DKQ-MKPVIKRSASWTC--------------	Solyc11g020610
WSIAGYLVAKMLLEDPSHLGMISLEE-DKQ-MKPVIKRSSSWTF--------------	Solyc06g065210
WSIAGYLVAKMLLEDPSHLGMISLEE-DKQ-TKPVIKRSYSWT---------------	At1G22650
WSIAGYLVAKMMLEDPSHLGMISLEE-DRAMLKPVLKRSASWTN--------------	GRMZM2G118737
WSIAGYLVAKMMLEDPSHLGMISLEE-DKAMLKPVLKRSASWTN--------------	Sb04g022350
WSITGYLVAKMLLEDPSHLGMIALEE-DKAMLKPVLKRSASWTN--------------	GRMZM2G170842
WSIAGYLVAKMLLEDPSHLGMIALEE-DKAM-KPVLRRSASWTN--------------	Bradi3g45530
WSIAGYLVAKMMLEDPSHLGMISLEE-DKAM-KPVLKRSASWTN--------------	LOC_Os02g34560
WSIAGYLVAKMMVEDPSHLGMISLEE-EKP-TKPVLRRSASWTG--------------	GRMZM2G136139
WSIAGYLVAKMMLEDPSHLGMISLEE-EKP-TKPVLRRSASWTG--------------	Sb04g029440
WSVAGYLVAKMMVEDPSHLGMISLEE-DRAMMKPVLKRSASWTV--------------	LOC_Os04g35280
WSIAGYLVAKMMLEDPSHLGMISLEE-DKA-MNPVLKRSASWTV--------------	Bradi5g10357
WSIAGYLVAKMMMDDPTHVGMISMEE-EKH-MKPPLRRSSSWT---------------	At1G72000
WSIAGYLVARMMLEDPSTLMMISMEE-DRP-VKPTMRRSASWNA--------------	GRMZM2G007277
WSIAGYLVARMMLEDPSTLMMISMEE-DRP-VKPTMRRSASWNA--------------	Sb05g004770
WSIAGYLVARMMLEDPSTLMMISMEE-DRP-VKPTMRRSASWNA--------------	GRMZM2G115451
WSIAGYLVARMMLEDPSTLMMISMEE-DRP-VKPTMRRSASWNA--------------	LOC_Os11g07440
WSIAGYLVARMMLEDPSTLMMISMEE-DRP-VKPTMRRSASWNA--------------	Bradi4g24470
WSVAGYLVAKMMLEDPSHLGAT-----------PTLGGCPNFLGLG------------	TnS000892793t06
WSIAGYLVAKMMLEDPSHLGMIGLEE-DKKM-KPSLTRSASWTA--------------	Pp3c4_10940
WSIAGYLVAKMMLEDPSHLGMIGLEE-DKKM-KPSLTRSASWTA--------------	Pp3c12_12810
WSIAGYLVAKMMLEDPSHLGMIGLEE-DKKIQKPSLTRSASWTA--------------	Pp3c3_25210
WSIAGYLVAKMMLEDPSHLGMIGLEE-DRKIQKPSLTRSASWTA--------------	Pp3c10_23170
WSIAGYLVAKMMLEDPSHLGMVSLEE-DKKT-KPYLTRSNSWTC--------------	Sm89558
WSVTGYLVAKMLLEDPSHLGLISLEE-DKKAAKPQLTRSVSWNGFMNL----------	kfl00164_0180_v1.1
WSVAGYLVAKMMLEDPSHLGMIVLEE-DKKLLKPKLTRSVSWTCLSNPR---------	TnS000602483t02
WSIAGYLVAKMLLEDPSHLGMIALGE-DRKI-EPALTRSASWTV--------------	TnS000105403t01
WSIAGYLVAKMLLEDPSHLGMIALGE-DTQI-KPALTRSASWTL--------------	TnS000982973t03
WSIAGYLVAKMLLEDPSHLGMISLEE-DRKG-KPVITRSASWTC--------------	Sm111393
WSIAGYLVAKMMLEDPSNLGLISLDG-DAKKGNGGMPRSASWTV--------------	Azfi_s0064.g035495

```
WSIAGYLVSKMMLDDPSYLGMVALHDVHPNEKSSFMTRSASWTF--------------                    Azfi_s0040.g026676
WSIAGYLVAKMLLEDPTHLGMVSLDE--DRNMKTLMTRSASWTV--------------                    Sm151264
WSIAGYLVAKMLLEDPSHLGMISIED-DRKTRTASIPRSASSTF--------------                    Azfi_s0393.g067806
WSIAGYLVAKMMLEDPSHLGMISLED-DKKTKSASMPRSASWT---------------                    Azfi_s0003.g008094
WSIAGYLVAKMMLEDPSHLGMIALEE-DKK-MKPTLVRSTSF----------------                    Azfi_s0059.g034661
WSIAGYLVSKMMLEDPSHLGMIALEE-DKK-MKPTLSRSASF----------------                    Azfi_s0001.g000547
WSITGYLVAKMMLENPSHLGMVSLDE-EKK-VQPTLTRSSSF----------------                    Azfi_s0096.g043780
CSIAGYLVSKMLLEEPSHLGIIALEE-DEKIKPTTITRSTTLPTKFRGYP--------                    GSVIVT01037429001
---------------------LSSSS-DKEH---------------------------                    Eucgr.D02386.1
WSIAGYLVAKMMVENPSNLLMISLEE-DKKSARSRLTRSNSTSF--------------                    Potri.002G173600
WSIAGYLVAKMMIENESNLLVISHEE-EKKTNKLRHTRSASCFF--------------                    GSVIVT01027176001
WSVAGYLVAKMLLDDPSHLRIVALEGDSHSR-APFLKRSNSCP---------------                    GRMZM2G477236
WSVAGYLVAKMLLDDPSHLRIVALEDDGHSR-APFLKRSNSCP---------------                    Sb04g002180
WSVAGYLVAKMLLDDPSNLRAVSLDDDGRGIREPVLKRSNSCP---------------                    Bradi3g02560
WSVAGYLVAKMLLDDPSNLRAVSLADDCHIRSAPVLKRSNSFP---------------                    LOC_Os02g03320
WSIAGYLVAKLLLANPAAAKFLTSEEDSDLRNAFSCMLSANPRRT-RGPKKAQQPFIV                    At5G22510
WSIAGYLVSKLLLSNPSAVRILANEEDADLVNAFSCMISSNPRRK-RGRPAVEKRFIV                    Eucgr.J00457.1
WSIAGYLVAKLLLADPSAARMLVTDEDPELVNAFSCMISSNPRRK-RGQKNSKKPFIV                    Potri.008G024100
WSIAGYLVAKLLLADPSAARMLVMDEDPELVSAFSCMISTHPRRN-RGQKNSKKTFMV                    Potri.010G236100
WSIAGYLVAKLLLSDPTAAKILITEEDSELVNAFSCMISANPRRK-RGRKSSTQTFIV                    GSVIVT01034753001
WSIAGYLVAKLLLADPGAAKILITDEDPALVNAFSCMIGANPRRK-RGRKNLKQSYIV                    Eucgr.G02704.1
WSIAGYLVSKLLLANPDAANILVNREDSDLVSAFSSMLSANPRRK-RDWKGLKQKFIV                    GSVIVT01031374001
WSISGYLVAKLFLANPSAAKIFVNEEDPELVNAL---ISANPRRK-RARKIFKQPFIV                    Potri.004G186500
WSIAGYLVAKLLLANPSSAKILISQEDSELLNAFSCAISSNPRRKKRGPKSPQKTYIV                    Solyc11g007270
WSIAGFLVAKLLIEKPDAARILWNDEDAEILNASS-------TNRKRGKKVLKKTYIV                    GRMZM2G040843
WSIAGFLVAKLLIEKPDAARILWNDEDAEILNALS-------TNRKRGKKVLKKTYIV                    Sb04g021550
WSIAGYLVAKQLLDKPDAARILWNDEDAEILNALS-------TNRKRGKKVLKKTYIV                    Bradi3g44770
WSIAGYLVAKQLLDKPDAARILSNDEDSEILNALS-------TNRKRGKKVLKKTFIV                    LOC_Os02g32730
WSIAGFLVAKLLLENPEKSRILWNNEDEEILNALSLMADASNSKRKRGRKVLKRTYIV                    Bradi5g09200
WTIAGFLVAKQLLENPDKSRILWNNEDEEILNAMNRMTDASNLKRRRGRKGLKKTYIV                    LOC_Os04g33490
WTIAGYLVAKLLIANPEAAKMVINVEDTELLSAFSSILSSNPRRK-RSRKGVKQSFII                    Solyc11g067050
WSIAGYLVSKQLLRNPEAAKSLTNEEDMALIDACFCLLEANPKRK-RSRKNVTKSFIV                    TnS000875687t08
---RGYLVAKMLLDKPDAVKILTCEEDLALLEAMSCSLDANPRIK-RKRKLKSTRIIV                    Sm267827
----------------------------------------------------------                    Azfi_s0345.g065806
WSIAGFLTAKLMLKNPNAASWLTCDEDE--TYAIF--REANP-QK-RKFKASPKIFVV                    Pp3c6_27130
WSIAGYLTSKLLSKNPDAANWLTCEEDD--HYAIL--LEANPNLK-RKFKASPNIFVV                    Pp3c16_15060
WSIAGYLTSKLLLKNPDAVKWLTCEDDE--DAASISCVDMNPRRN-PDLKPQGDRHIV                    Pp3c17_13250
WSIAGYLVAKQLLANPEAAAYLTCEEDPGLLDAFSCRISS---------KQPKKA---                    Sm443960
WTIAGFLVARKLLEDPSMVDLFACDEDDTIVAACSCMLDGDVRKS----DFWSDSD--                    kfl00563_0120_v1.1
```

Supplemental Figure S2 Multiple sequence alignment of CIN proteins from α1and β family in plant species tested. The CLUSTAL W software was used. Blue dotted line: N-terminal sequences

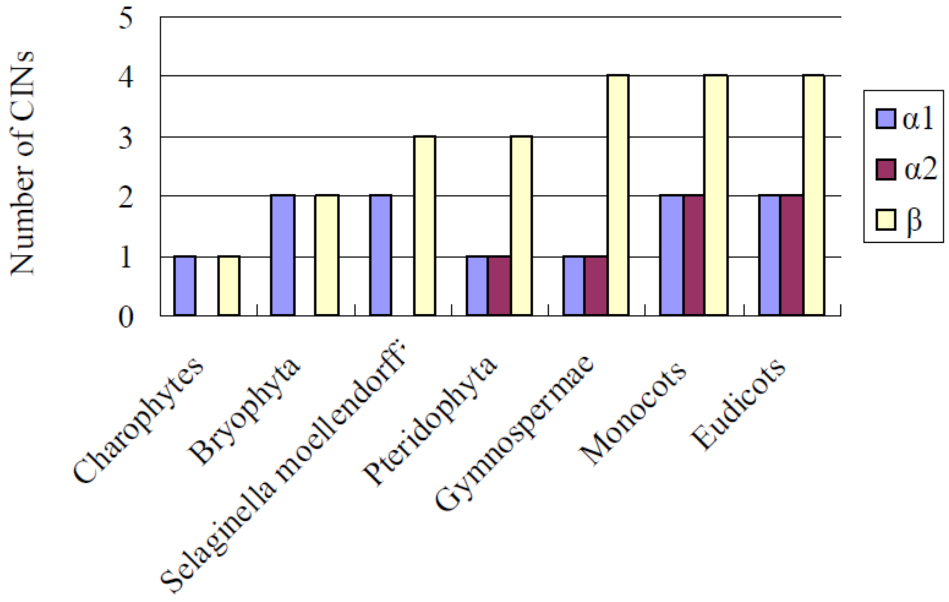

Supplemental Figure S3 Variation of numbers of CINs in different plant species. No α2 subfamily members were identified in *Selaginella moellendorff*, a basal vascular plant, however, one member of α2 CIN appeared in Pteridophyta.
