# Supplementary material for: Evolution of cytosolic and organellar invertases empowered the colonization and thriving of land plants
Source: Plant Physiol. 2023 Jul 11;193(2):1227–43. doi: 10.1093/plphys/kiad401 (PMC10661998; doi:10.1093/plphys/kiad401)
Supplement: kiad401_Supplementary_Data [file kiad401_supplementary_data.zip › Supplemental Table S4.pdf]

Supplemental Table S4 Major MEME (Multiple Em for Motif Elicitation) motifs in predicted CINs*

Name	Width	Motif sequence	Motif sequence conservation
MEME-1	50	SFHIREYYWLDFQQLNEIYRYKTEE<br>YSHTAVNKFNVIPDSIPDWLFDFMP	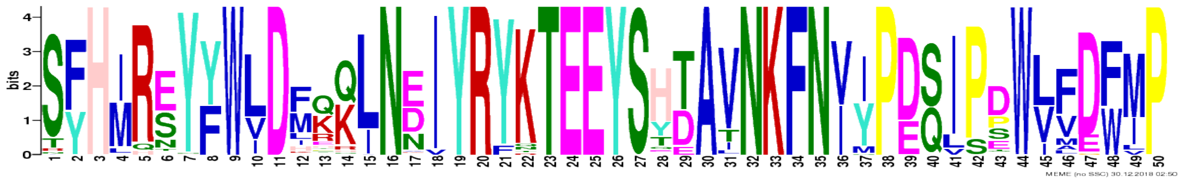
MEME-2	41	ERWEDLVGEMPLKICYPALEGHEW<br>RIITGCDPKNTRWSYHN	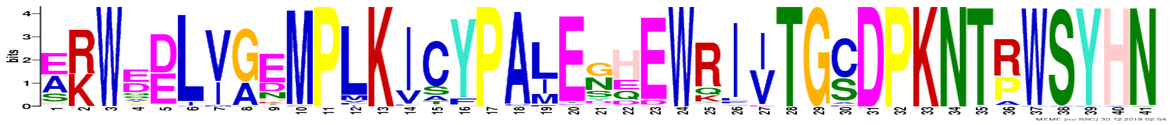
MEME-3	41	CQTGIRLILSLCLSDGFDTFPTLLCA<br>DGCCMIDRRMGIYGY	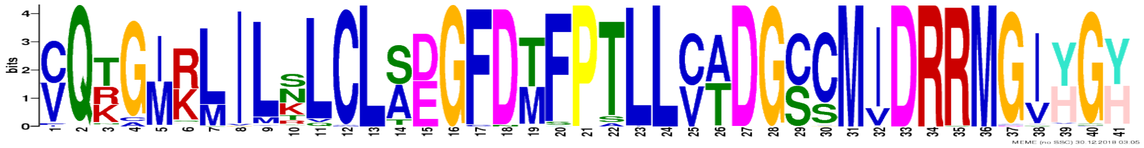
MEME-4	41	WPEYYDGKLGRFIGKQARKFQTW<br>SIAGYLVAKMLLEBPSHL	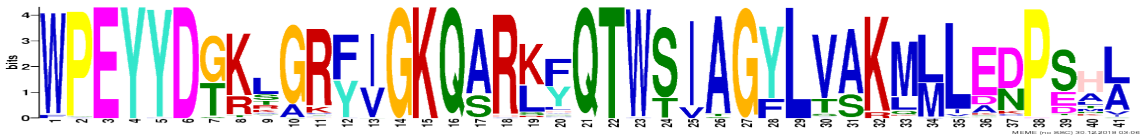
MEME-5	29	ADFGESAIGRVAPVDSGFWWIILLR<br>AYTK	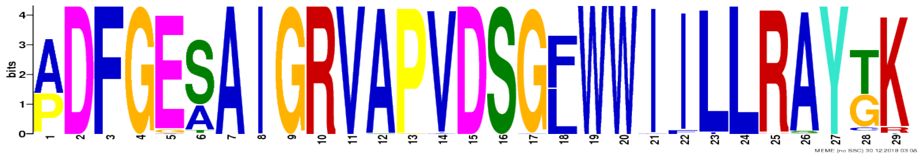

*https://meme-suite.org/meme/tools/meme

MEME-6	21	NYDQVFVRDFVPSALAFLMNG	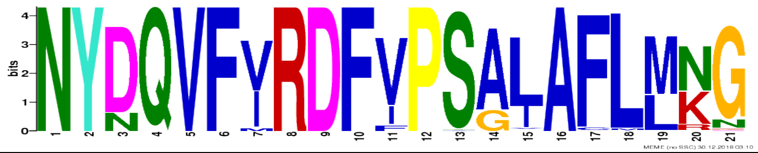
MEME-7	41	GGYFIGNVSPARMDFRWFALGNCWAILSSLATPEQSEAIMD	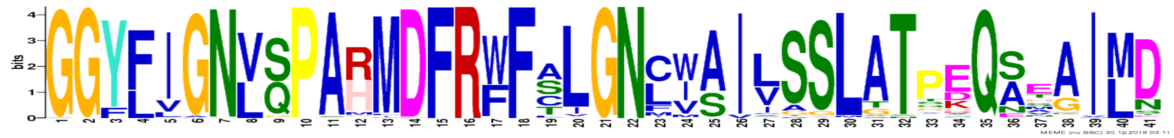
MEME-8	26	EKTVDCFSLGZGLMPASFKVLHDPLR	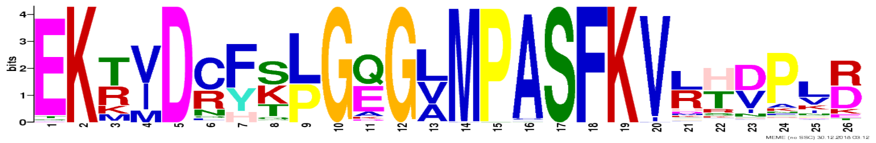
MEME-9	31	EAWELLRRSVVYFRGQPVGTIAANDPSDEEL	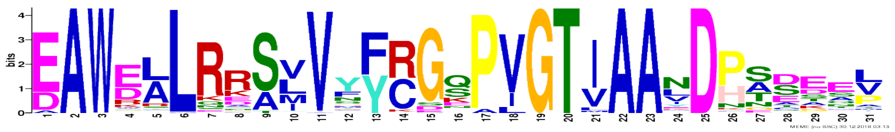
MEME-10	21	GGSWPVLLWLLTAACIKTGRP	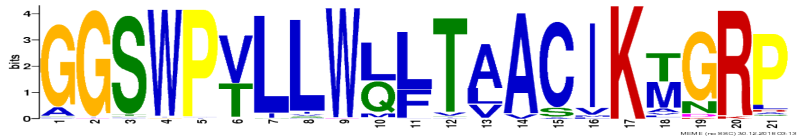
MEME-11	21	PJEIQALFYMALRCAREMLKP	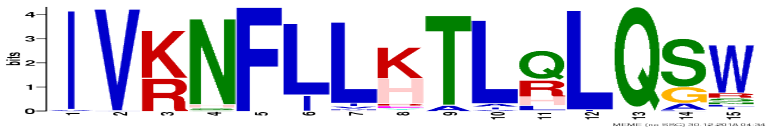

MEME-12	15	IVKNFLLKTLQLQSW	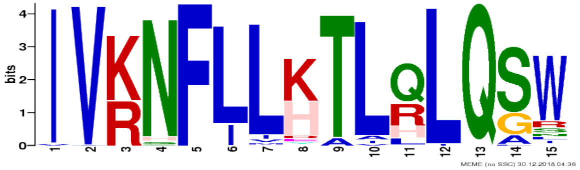
MEME-13	15	ARRAIELAEKRLSKD	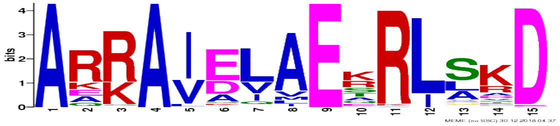
MEME-14	15	GKELIERINKRLHAL	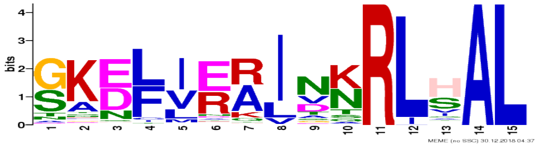
MEME-15	11	TGDLSLAERPD	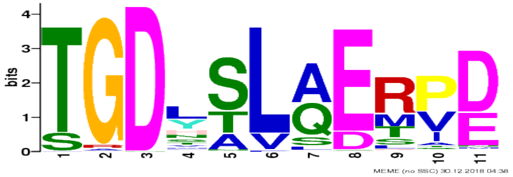
MEME-16	15	GMISLEEDKQMKPVL	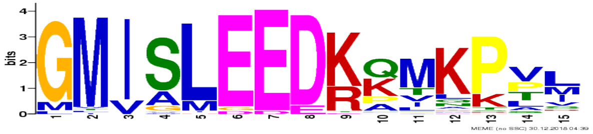
MEME-17	15	NIERQRSFDERSLSE	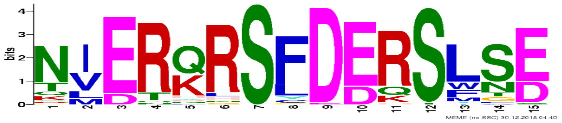

MEME-18	21	CEEDLELLEACSCMLSKNPRR	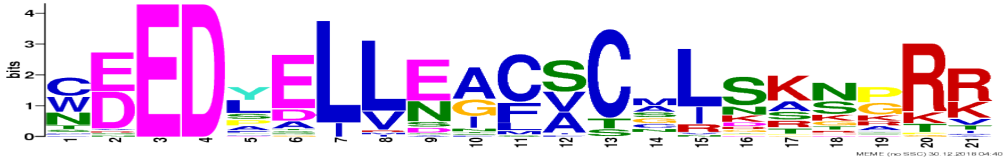
MEME-19	15	ASSARHSFEPHPMVA	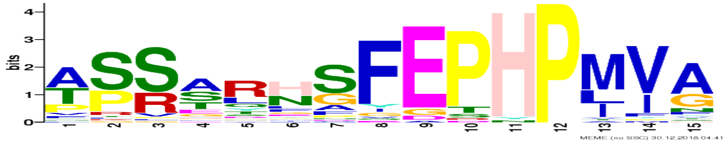
MEME-20	50	VIASVASDVRNFSTSVETRVNDKNF ERIYIQGGLNVKPLVIERIEKDEBV	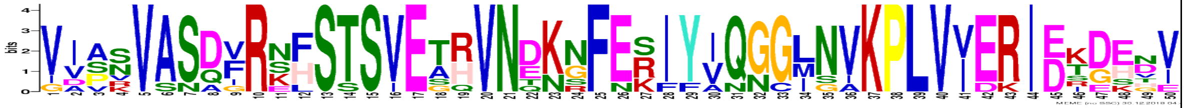
